# Acceptability and safety of one versus three months of rifapentine and isoniazid to prevent tuberculosis in people exposed in the household or workplace in Brazil: The Ultra-Curto randomized controlled trial

**DOI:** 10.1371/journal.pmed.1004758

**Published:** 2026-02-10

**Authors:** Betina Durovni, Marcelo Cordeiro-Santos, Solange Cesar Cavalcante, Renata Spener-Gomes, Jamile Garcia, Silvia Cohn, Valeria Saraceni, Beatriz Kohler, Lawrence H. Moulton, Alexandra Brito da Souza, Abiye Berihun, Mark Marzinke, Richard E. Chaisson, the Ultra Curto Study Team

**Affiliations:** 1 Secretaria Municipal de Saúde do Rio de Janeiro, Rio de Janeiro, Brazil; 2 Fundação de Medicina Tropical “Doutor Heitor Vieira Dourado”, Manaus, Brazil; 3 Pós-Graduação em Medicina Tropical, Universidade do Estado do Amazonas, Manaus, Brazil; 4 Pós-Graduação em Saúde e Inovação, Universidade Nilton Lins, Manaus, Amazonas, Brazil; 5 Faculdade de Medicina, Universidade Federal do Amazonas, Manaus, Brazil; 6 Center for Tuberculosis Research, Division of Infectious Diseases, Johns Hopkins University School of Medicine, Baltimore, Maryland, United States of America; 7 Department of International Health, Johns Hopkins Bloomberg School of Public Health, Baltimore, Maryland, United States of America; 8 Department of Pathology, Johns Hopkins University School of Medicine, Baltimore, Maryland, United States of America; 9 Division of Clinical Pharmacology, Department of Medicine, Johns Hopkins University School of Medicine, Baltimore, Maryland, United States of America; Pasteur Network, FRANCE

## Abstract

**Background:**

Short-course tuberculosis preventive therapy with isoniazid and rifapentine (HP) is widely recommended, but the acceptability and safety of one month of daily HP (1HP) compared to three months of weekly HP (3HP) is uncertain. We compared treatment with these two regimens in people with a positive latent tuberculosis infection test and without HIV infection. We hypothesized that 1HP would have greater treatment completion and fewer targeted safety events than 3HP.

**Methods and findings:**

We conducted a Phase 4 randomized trial of 1HP versus 3HP in adolescents and adults without HIV infection with recent tuberculosis exposure and a positive latent tuberculosis infection test in two sites in Brazil. The primary outcomes were successful completion of >90% of medication as ascertained by self-report, pill counts, and pharmacologic monitoring, and safety. Treatment safety was defined as occurrence of Grade >2 targeted events or discontinuation of treatment for side effects. We randomized 500 individuals to 1HP (249) and 3HP (251); 193 males and 307 females, with a median age of 39 years. Treatment completion was 89.6% for 1HP recipients versus 84.1% for 3HP recipients (site-adjusted risk difference 5.2%, [95% CI: [−0.1%, 11.2%], *p* = 0.10). Targeted >Grade 2 adverse safety events or treatment discontinuation occurred in 16.1% of 1HP recipients and 10.4% of 3HP recipients (site-adjusted risk difference 6.1%, [95%CI: [−0.04%, 12.3%], *p* = 0.05). The proportions who discontinued treatment for any side effect were 7.2% for 1HP and 4.4% for 3HP. The risk difference for the primary safety outcome adjusted for site and baseline demographic and clinical covariates was 3.4% (95% CI [−2.3,9.1%], *p* = 0.24). The trial was not designed to ascertain efficacy.

**Conclusion:**

Both 1HP and 3HP had high rates of treatment success. Participants assigned to 1HP had more targeted safety events, mostly low-grade. Neither regimen was superior to the other. These results will inform global guidelines for tuberculosis preventive therapy. NCT04703075 (clinicaltrials.gov).

## Author summary

**Why was this study done?:**

- Tuberculosis Preventive Therapy (TPT) is an important method for reducing the incidence of tuberculosis that previously required 6–9 months of treatment.
- Two new shorter treatment regimens (1HP and 3HP) have been shown to be efficacious, but 1HP has only been studied in people with HIV infection and the safety of the regimens has not been compared in a head-to-head trial.

**What did the researchers do and find?:**

- We performed a randomized clinical trial comparing treatment success, defined as completing >90% of medication, and safety of these two regimens in 500 adolescents and adults without HIV infection who had positive tests for TB infection after exposure to someone with active TB in their household or workplace.
- We found that both 1HP and 3HP had high rates of treatment success and low rates of adverse safety effects.

**What do these findings mean?:**

- This study of 1HP in people without HIV infection demonstrates that the regimen is safe and has high completion rates.
- Both regimens had similar rates of completion and the 3HP regimen had slightly lower rates of adverse reactions.
- These data show that both regimens can be used by clinicians and public health programs as options for TPT.
- The study was not designed to determine whether either regimen was more effective than the other due to the small sample size.

## Abstract 3

In a multi-center, randomized controlled trial, Betina Durovni and colleagues assess the acceptability and safety of a short-course tuberculosis preventive therapy taken daily or weekly for different durations by persons recently exposed to tuberculosis. short version: In a randomized controlled trial, Betina Durovni and colleagues assess the acceptability and safety of a short-course tuberculosis preventive therapy for different durations.

## Introduction

The World Health Organization has prioritized tuberculosis preventive therapy (TPT) for people with tuberculosis infection (TBI) who are at high risk of developing active disease as a key strategy for controlling the epidemic [1]. Isoniazid for 6–9 months is effective, reduces tuberculosis morbidity and mortality, and has been the mainstay of TPT for decades, but global uptake has been abysmal. Completion rates for isoniazid preventive therapy are poor, with a large proportion of patients unable to complete treatment [2–4]. While uptake is influenced by a variety of factors, a critical element has been the duration of isoniazid treatment, with adherence falling sharply over time in clinical trials and practice as patients tire of taking medicine [3,5,6]. Shorter course regimens have a much higher completion rate and are more acceptable to patients, clinicians, and programs.

Three months of supervised weekly rifapentine and isoniazid (3HP) in people with and without HIV infection is non-inferior to longer courses of isoniazid, with better adherence and less toxicity [7–9]. Self-administration of 3HP has somewhat poorer adherence but is recommended in some guidelines. [10,11]. One month of self-administered daily rifapentine and isoniazid (1HP) was found to be non-inferior to nine months of isoniazid in people with HIV infection [12]. The availability of two innovative short-course TPT regimens offers a transformative opportunity for global tuberculosis control and achieving the End TB targets. However, the safety of the 1HP regimen in people without HIV infection has not been thoroughly evaluated, and the relative acceptability of 1HP versus 3HP is unknown. Studies of TPT using rifampicin and pyrazinamide were found to be safe and effective in people with HIV, for example, but subsequent use in people without HIV was associated with unacceptable hepatotoxicity [7,13–15]. Therefore, we undertook the Ultra Curto trial to compare treatment adherence and safety of 1HP to 3HP in adolescents and adults without HIV infection who had recent household or occupational exposure to tuberculosis and a documented positive test for TBI. Because TPT regimens that are efficacious in one population have historically been efficacious in others, we did not power the trial to assess efficacy.

## Methods

### Trial design and participants

We studied people 15 years and older who were either household contacts of an individual with confirmed rifampicin-susceptible tuberculosis disease and had a positive test for TBI, or who had occupational exposure to patients with tuberculosis and whose test for TBI had converted from negative to positive in the prior two years. Children younger than 15 were not included because dosing information for daily rifapentine in this age group has not been studied. The study was performed in two Brazilian cities: in Rio de Janeiro participants were recruited at a public health clinic serving several communities with high rates of tuberculosis disease, and in Manaus participants were recruited at a public foundation that serves as a state reference center for HIV, providing prevention and treatment for tropical and infectious diseases to residents of Amazonas state. Potentially eligible participants were identified during contact evaluations of newly diagnosed patients with rifampicin-susceptible tuberculosis or from an occupational health program testing healthcare workers exposed to tuberculosis. Individuals with a positive tuberculin skin test (>10 mm induration) or a positive QuantiFERON Gold Plus test (Qiagen, Germantown, MD) were invited to undergo screening after signing informed consent and assent for those <18 years old. Individuals who were not able to read had the consent form read to them and signed with a fingerprint with an independent witness. Active tuberculosis disease was ruled out by symptom review and chest X-ray, and people with HIV infection, blood dyscrasias, elevated liver enzyme, pregnancy, or peripheral neuropathy were excluded. Other exclusion criteria included known intolerance of study medications, weight <40 kg, known contact with a patient with rifampicin- or isoniazid- resistant tuberculosis, previous treatment for active tuberculosis or TBI, or required use of a medication contraindicated with rifapentine or isoniazid. This study is reported as per CONSORT 2025 guideline (S1 Checklist).

### Randomization, masking, treatment, and assessments

Participants were randomized by a STATA-generated schedule, stratified by site, with randomly permuted blocks of random sizes 4, 6, and 8. Treatment assignment employed opaque envelopes maintained at each site (Rio de Janeiro and Manaus). The envelopes were prepared by study staff uninvolved in recruitment or enrollment and opened sequentially according to time of enrollment in each site. Contacts of the same index case were considered a cluster and were assigned to the same treatment arm according to the randomization of the first enrolled contact. Cluster size was limited to 2 participants per index case due to the diminishing returns in terms of power per enrollee within a household. The study was a pragmatic trial measuring adherence and tolerability, so treatment assignment was not blinded. Participants were randomized to receive either rifapentine and isoniazid daily for 4 weeks (1HP) or rifapentine and isoniazid weekly for 12 weeks (3HP). Rifapentine was dispensed at 600 mg daily (1HP) or 900 mg weekly (3HP), and isoniazid was dispensed as 300 mg daily (1HP) or 900 mg weekly (3HP). Participants were instructed to take their medication with food. The first dose of medication was given in the study clinics and the remainder was self-administered. Participants in the 1HP arm were seen for monitoring and follow-up visits at 2, 4, 8, 12, 16, and 24 weeks after enrollment, and those on the 3HP arm were seen at 2, 3, 8, 11, 16, and 24 weeks after enrollment. The difference in scheduling of follow-up visits was to allow for assessments of adherence after 2 and 4 weeks of treatment for 1HP and after 4 weekly doses (week 3) and 12 weekly doses (week 11) for 3HP. At each study visit while taking treatment medications participants had a complete blood count and liver enzyme activity measurements, as well as assessments of adherence and adverse safety events.

Adherence was assessed by patient self-report, pill counts, and testing of whole blood collected as dried blood spots for rifapentine and of urine for isoniazid. Participants were given treatment diaries to record the time and date of each dose of medication taken and instructed to bring these and any remaining pills to clinic visits. Data from the diaries and pill count were entered onto case report forms. At two study visits, participants had blood and urine collected for pharmacologic monitoring. Dried blood spots (DBS) were prepared from venous blood collected in potassium-EDTA tubes from which 25 µl was spotted on Whatman Protein Saver 903 cards. After air-drying, DBS cards were stored at ambient temperature in plastic bags protected from light and subsequently shipped to the Johns Hopkins University Clinical Pharmacology Analytical Laboratory for bioanalysis. Estimated plasma rifapentine concentrations were measured from a 3 mm DBS punch; briefly, rifapentine was extracted from the DBS card with a 9:1 ratio of methanol to 50 mM ammonium formate in water containing 1 mg/mL ascorbic acid using a modified version of a previously described method [16]. Rifapentine was quantified via liquid chromatography-tandem mass spectrometry using an API45S00 (SCIEX, Foster City, CA) operated in positive ionization and selective reaction monitoring modes with a lower limit of quantitation of 50 ng/mL and the assay was validated in accordance with applicable regulatory requirements. Fresh urine was collected in the clinic and subsequently tested for the presence of isoniazid and metabolites using the IsoScreen test (GFC Diagnostics, Chipping Warden, UK), following the manufacturer’s instructions. If test kits were not available, urine was frozen then thawed and tested when kits became available. The test causes urine to change color from yellow to green (low levels) or blue-black (high levels) if isoniazid metabolites are detected [17]. The sensitivity of any positive result has been estimated as 95%–99% at 24 hours and 85% at 48 hours after the last dose of 300 mg of isoniazid, and specificity is 87% at 72 hours. Testing was performed by laboratory technicians who were unaware of treatment assignment, and results were not shared with study clinicians or participants.

During the course of the trial a number of protocol deviations were noted, largely related to timing of study visits and collection of specimens. None of these affected the study integrity or outcomes. The protocol and amendments are included in the S1 and S3 Texts.

### Study outcomes and sample size

**Fig 1:**
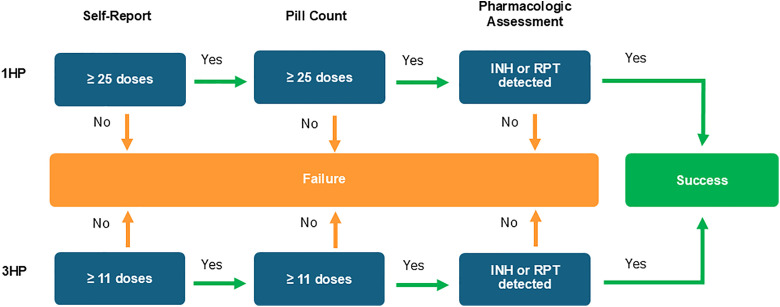
Analytic Strategy and for Treatment Success. Pharmacologic monitoring was performed at 2 visits. A positive result on either test at either visit was considered evidence of adherence. Abbreviations: HP, isoniazid and rifapentine; INH, isoniazid; RPT, rifapentine.

The primary outcomes of the trial were successful completion of treatment and safety. For 1HP, successful completion of treatment was defined as having taken at least 25 doses of self-administered daily medication within 6 weeks of enrollment, confirmed by patient report and pill count, and having rifapentine or isoniazid detected in at least one blood or urine specimen. For 3HP, successful completion was defined as having taken at least 11 doses of self-administered weekly medication within 18 weeks as confirmed by patient report, pill count, and having rifapentine or isoniazid detected in at least one blood or urine specimen. The analytic strategy is shown in Fig 1. The safety outcome was occurrence of Grade 2 or higher targeted safety events, defined as hypersensitivity syndrome, rash, peripheral neuropathy, hepatotoxicity, nausea, vomiting, and drug-related fever using the Division of AIDS Adverse Events Grading Table. Individuals who stopped treatment because of any side effect were also classified as having an unsuccessful safety outcome. A secondary outcome of cost-effectiveness will be reported separately. Tuberculosis incidence was an exploratory outcome and the results are included here.

Based on adherence data from previous trials, we hypothesized that successful treatment with 1HP would be superior to 3HP, with 90% of individuals randomized to 1HP taking >90% of prescribed doses versus 80% of those taking 3HP [9,10,12]. We hypothesized that the safety of 1HP would be superior to 3HP, with a rate of targeted adverse safety events or medication discontinuation of 6% versus 13% for 3HP. A sample size of 500 evaluable participants, 250 individuals per arm, with an average cluster (household contacts of the index case) size of nearly 2, and an outcome coefficient of variation (CV) of 0.15, near the mean bound using the maximum entropy distribution [18], provided 80% power for detecting a significant difference at a two-sided 0.05 significance level. The sample size was not inflated for losses to follow up, as those who failed to complete sufficient doses (25 and 11 for 1HP and 3HP, respectively) were considered to have reached a study endpoint.

In previous large trials of 3HP, rates of drug-related adverse safety events were 7%–8% and in clinical cohort studies discontinuation rates for adverse safety events have been higher, ranging from 12%–18% [9,10,19]. We conservatively assumed an adverse safety event discontinuation rate for 3HP of 13%. In the BRIEF TB trial, which used the same definition of targeted safety events but only analyzed Grade 3 or higher, the rate of targeted safety events was 3% and of serious adverse events was 5% [12]. We assumed that the risk of targeted safety events or discontinuations would be 6% in in people on 1HP in this trial since we also included Grade 2 events. With a CV of 0.1 and a sample size of 250 individuals in each study arm, we had about 80% power for detecting a significant difference at a two-sided 0.05 significance level. We also had 80% power to distinguish between risks of 12% and 5% in the two study arms. Although this was phrased as a hypothesis test, our goal was to estimate the difference in safety risks. With this sample size, and risks of 13% and 6% with 3HP and 1HP, respectively, the 95% CI for the risk difference (RD) had a half-width of 3 percentage points [20].

### Statistical analyses

Analyses used the intention-to-treat population for both primary outcomes of successful completion and safety from the time of the initial study medication dose. No multiplicity correction was employed. The site-adjusted RD was the primary parameter of interest for both primary outcomes. Within-household correlation was accounted for using generalized estimating equations (GEE) with binary response, exchangeable correlation structure, and identity link function to estimate the RD, with a dummy variable included to account for site. For convenience and convergence considerations, variable selection for the adjusted models was done using generalized linear models with a log link function. Subgroup analyses were done for the prespecified variables: site, sex, age, weight. Interaction *p*-values were determined using GEE, but with log link to reduce the risk of non-convergence. Variables included in covariate-adjusted models were first screened for statistical significance and effect size; final models were determined via backward stepwise selection with *p* > 0.2 to remove, and *p* < 0.1 to remain. The authors had control of and verified the data and performed all analyses. The Statistical Analysis Plan is included in the S2 Text.

### Regulatory review

The trial was registered on clinicaltrials.gov (NCT04703075). Ethical approval was provided by the Johns Hopkins Medicine Institutional Review Board, the Brazilian Comissão Nacional de Ética em Pesquisa, and the Committees on Research Ethics of the Rio de Janeiro Municipal Secretariat of Health and the Fundação de Medicina Tropical “Dr. Heitor Vieira Dourado.” The trial was registered at clinicaltrials.gov on 7 January 2021 (NCT04703075). The protocol was amended three times during the trial to clarify language regarding endpoints and missed visits, to make the duration of time allowed to complete treatment proportional in both arms (duration plus 50%), correct typographical errors, to eliminate blood bicarbonate testing, clarify timing of pharmacologic monitoring, and to use convenience sampling for cost-effectiveness interviews.

## Results

**Fig 2:**
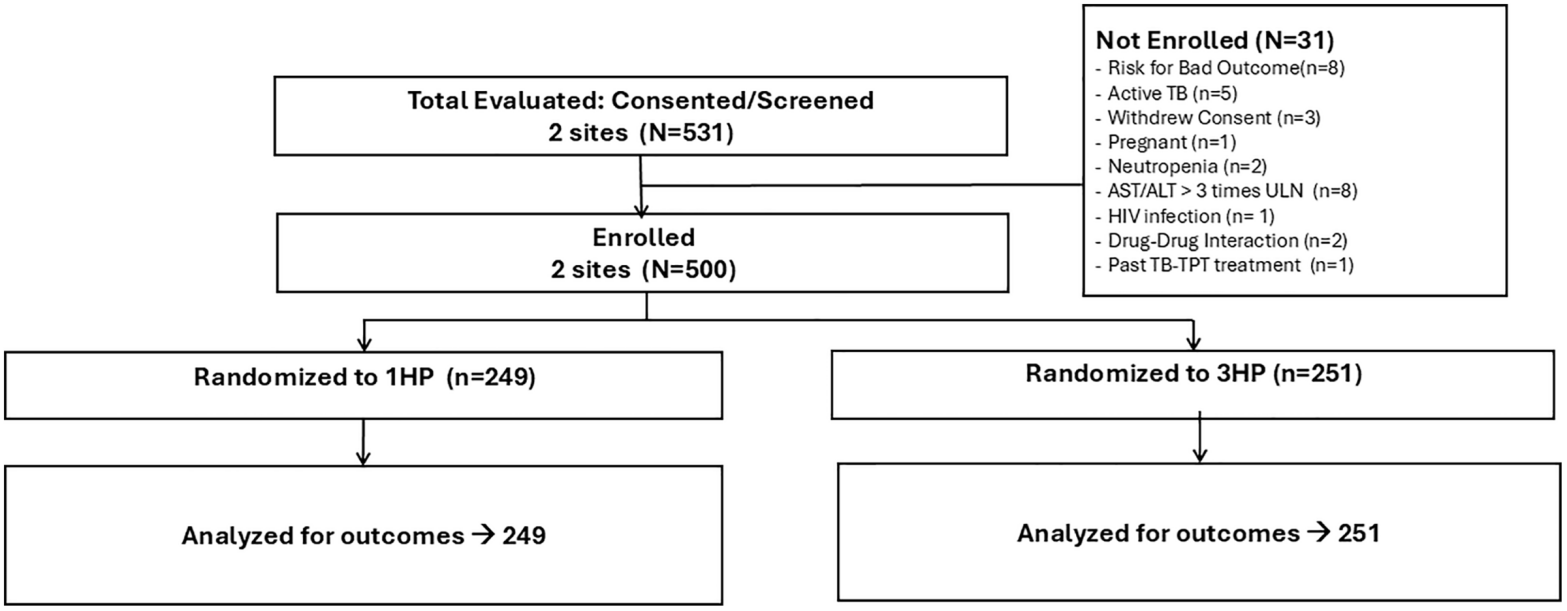
Consort diagram.

Between March 28, 2022, and December 28, 2023, we screened 531 individuals and enrolled 500 individuals at the two trial sites in Rio de Janeiro and Manaus, with 249 assigned to the 1HP arm and 251 assigned to 3HP; all were included in the analysis (Fig 2). Demographic and clinical characteristics of participants in the two study arms were similar (Table 1). The median age was 39 years; 61.4% were female; the median BMI was 27.8; and 95.4% were contacts of a person with active tuberculosis, including 340 in clusters of two who were contacts of the same index patient. TBI was documented by tuberculin skin test in 299 (59.8%) and by interferon-gamma release assay in 201 (40.2%).

**Table 1:** Baseline characteristics of participants enrolled in the trial.

		1HP (*N* = 249)	3HP (*N* = 251)	Total (*N* = 500)
Inclusion criterion	Household contact of person with TB	238 (95.6%)	239 (95.2%)	477 (95.4%)
	Conversion of TST or IGRA (last 2 years)	11 (4.4%)	12 (4.8%)	23 (4.6%)
Sex at birth	Female	167 (67.1%)	140 (55.8%)	307 (61.4%)
	Male	82 (32.9%)	111 (44.2%)	193 (38.6%)
Age (Years)	Median (interquartile range)	40.0 (28.0–54.0)	38.0 (25.0–52.0)	39.0 (25.5–53.0)
Race/skin color^*^	Brown	141 (56.6%)	155 (61.7%)	296 (59.2%)
	White	70 (28.1%)	70 (27.9%)	140 (28.9%)
	Black	38 (15.3%)	23 (9.2%)	61 (12.2%)
	Asian	0 (0.0%)	3 (1.2%)	3 (0.6%)
Education	None	5 (2.0%)	6 (2.4%)	11 (2.2%)
	1–5 years	25 (10.0%)	20 (8.0%)	45 (9.0%)
	6–14 years	77 (30.9%)	63 (25.1%)	140 (28.0%)
	15–18 years	118 (47.4%)	133 (53.0%)	251 (50.2%)
	>19 years	24 (9.6%)	29 (11.5%)	3 (0.6%)
Weight	Median (interquartile range)	71.5 (62.2–82.4)	72.0 (60.7–83.9)	71.5 (61.8–83.3)
Body mass index	Median (interquartile range)	27.8 (24.0–31.6)	27.8 (23.6–31.0)	27.8 (23.8–31.2)
Latent TB infection test	Tuberculin skin test	149 (59.8%)	150 (59.8%)	299 (59.8%)
	Interferon-gamma release assay	100 (40.2%)	101 (40.2%)	201 (40.2%)
Laboratory results	Hematocrit (%)	41.1 (38.9,43.8)	41.7 (38.8,45.1)	41.5 (38.9–44.7)
	Hemoglobin (g/dL)	13.7 (12.9–14.7)	13.7 (12.8–15.1)	13.7 (12.9–14.9)
	Total bilirubin	0.41 (0.30–0.61)	0.43 (0.31–0.62)	0.42 (0.31–0.61)
	Aspartate aminotransferase (SGOT) (U/L)	20 (17–25)	21 (18–26)	20 (17–25)
	Alanine transaminase (ALT) (U/L)	19 (14–30)	21 (15–30)	21 (15–30)
	Alkaline phosphatase	129 (76–217)	135 (77–226)	132 (76–220)

TB, tuberculosis; TST, tuberculin skin test; IGRA, interferon-gamma release assay; 1HP, one month of daily isoniazid and rifapentine; 3HP, three months of weekly isoniazid and rifapentine.

*Descriptions of skin color and race follow the official standardized categories established by the Brazilian Institute of Geography and Statistics for the Brazilian Census.

As shown in Table 2 and S1 Fig, successful completion of treatment in the 1HP arm was 89.6% while in the 3HP arm it was 84.1% (site-adjusted RD 5.2%, 95% confidence interval [CI] [−0.9, 11.2], *p* = 0.10). After adjustment for baseline covariates, the RD for successful completion of treatment was 5.3% (95% [CI −0.3, 10.9], *p* = 0.06). Pharmacologic testing reduced the proportion of participants who completed treatment with 1HP by 1.6% compared to self-report and pill count, while the decrement with 3HP was 4.3% (S1 Fig) The median time between the self-reported last dose and sampling for pharmacological testing was 0.9 days (IQR 0.6–1.1 d) in the 1HP arm and 0.9 days (IQR 0.7–1.8 d) in the 3HP arm.

**Table 2:** Study outcomes: treatment completion and safety (targeted safety events and discontinuation due to safety events).

Treatment outcomes	1HP *N* = 249	3HP *N* = 251	Site-adjusted risk difference^*^ (95%CI), *p*-value	Covariate-adjusted risk difference (95%CI), *p*-value^**^
Successful completion	223 (89.6%)	211 (84.1%)	5.2% (−0.9,11.2), 0.10	5.3% (−0.3, 10.9), 0.06
Safety	40 (16.1%)	26 (10.4%)	6.1% (−0.04,12.3), 0.05	3.4% (−2.3, 9.1), 0.24
Targeted safety event^*^	36 (14.5%)	23 (9.2%)	5.8% (0.07,11.5), 0.05	–
Discontinued treatment due to targeted event	14 (5.6%)	8 (3.2%)	2.4% (−1.0, 5.9), 0.25	–
Discontinued treatment for any adverse event	18 (*7*.2%)	11 (4.4%)	2.9% (−1.3, 7.1), 0.18	–

*Risk difference accounting for within-cluster correlation and adjusted for site.

**Risk difference accounting for within-cluster correlation and adjusted for site and baseline covariates.

Subgroup analyses showed a significantly higher completion rate with 1HP for participants in Manaus, with only a modest difference in Rio de Janeiro (Table 4). Male participants had slightly higher completion rates than females with both regimens. A sensitivity analysis using a stricter definition of adherence requiring pharmacologic evidence of rifapentine or isoniazid use at both testing occasions showed that successful treatment was 82.7% for 1HP and 63.8% for 3HP (RD 19.0%, 95% CI [11.1, 26.9], *p*-value <0.0001).

**Table 4:** Subgroup analyses of study outcomes.

Subgroup analysis adjusted by site		1HP/3HP *N* = 500	1HP *N* = 249		3HP *N* = 251		*p*-value	*p*-value for interaction
**Successful treatment completion**		***n*/*N***	***n*/*N***	**1HP % completion (95% CI)**	***n*/*N***	**3HP % completion (95% CI)**		
Site	Manaus	212/250	110/123	89.8 (84.0, 95.6)	102/127	80.4 (73.0, 87.8)	**0.05**	0.16
	Rio	221/250	112/126	88.8 (83.7, 94.0)	109/124	87.9 (82.0, 93.8)	0.82	
Sex	Female	262/307	147/167	88.1 (83.3, 92.9)	115/140	82.1 (75.5, 88.6)	0.15	0.91
	Male	172/193	76/82	91.3 (85.6, 97.0)	96/111	87.5 (81.8, 93.2)	0.33	
Age	Younger, <40 years	221/253	112/122	91.8 (86.9, 96.7)	109/131	84.0 (77.5, 90.6)	**0.06**	0.30
	Older, ≥40 years	213/247	111/127	87.4 (81.8, 93.0)	102/120	85.2 (78.9, 91.5**)**	0.60	
Weight	Lighter (<7.2 kg)	214/250	113/128	88.1 (82.4, 93.8)	101/122	82.8 (76.1, 89.6)	0.24	0.99
	Heavier (≥72 kg)	220/250	110/121	90.3 (85.2, 95.4)	110/129	85.8 (79.6, 91.9)	0.26	
**Safety**								
Site	Manaus	37/250	21/123	17.9 (10.7, 25.1)	16/127	11.7 (5.6, 17.8)	0.21	0.62
	Rio	29/250	19/126	15.1 (8.5, 21.7)	10/124	8.1 (3.4, 12.8)	**0.09**	
Sex	Female	48/307	32/167	19.2 (13.0, 25.3)	16/140	10.4 (5.0, 15.9)	**0.05**	0.32
	Male	18/193	8/82	9.7 (3.2, 16.1)	10/111	9.0 (3.8, 14.3)	0.88	
Age	Younger, 40 yr	27/253	13/122	10.7 (5.2, 16.2)	14/131	10.7 (5.1, 16.2)	0.99	0.13
	Older, ≥40 years	39/247	27/127	21.7 (14.1, 29.3)	12/120	10.0 (4.4, 15.7)	**0.02**	
Weight	Lighter, < 72 kg	42/250	24/128	18.8 (12.0, 25.6)	18/122	14.5 (7.8, 21.2)	0.39	0.99
	Heavier, ≥72 kg	24/250	16/121	13.2 (6.9, 19.4)	8/129	6.2 (2.0, 10.4)	**0.07**	

For individuals sampled within 24 hours of completing their regimen, the median (IQR) rifapentine concentrations in the 1HP and 3HP regimens were 7,719 ng/mL (IQR: 5,563, 10,300) and 6,910 ng/mL (IQR: 4,281,10,040), respectively; 7 (2.3%) and 20 (4.4%) participant samples had unquantifiable rifapentine concentrations within 24 hours of self-reported last dose taken (S2 Fig).

For the primary safety outcome, a targeted safety event or treatment discontinuation due to side effects occurred in 40 (16.1%) of those assigned to 1HP and 26 (10.4%) of those assigned to 3HP (RD = 6.1%, 95% CI [−0.04, 12.3], *p*-value = 0.05). After adjustment for baseline covariates, the RD for the primary safety outcome was 3.4% (95% CI [−2.3, 9.1], *p* = 0.24). Specific adverse safety events for both arms are shown in Table 3. Targeted adverse safety events in the 1HP arm occurred in 22 participants (8.8%) who did not discontinue treatment and 14 (5.6%) who did discontinue treatment. Another 4 participants (1.6%) in the 1HP arm discontinued treatment due to other side effects. In the 3HP arm, 15 individuals (6.0%) had a targeted adverse safety event but did not discontinue treatment and eight (3.2%) discontinued due to a targeted event. Another three participants (1.2%) in the 3HP arm discontinued for other side effects. The most common Grade 2 or higher adverse safety events were hepatotoxicity (5.2% for 1HP recipients and 4.0% for 3HP recipients) and rash or urticaria (4.8% for 1HP recipients and 0% for 3HP recipients). Grade 3 or higher targeted adverse safety events occurred in 9 1HP recipients (3.6%) and 7 3HP recipients (2.8%). Serious adverse events were reported in one 1HP recipient and two 3HP recipients, none of which were considered treatment-related. During the 6-month follow-up period no episodes of active tuberculosis were reported.

**Table 3:** Participants experiencing Grade 2 or higher targeted adverse safety events.

	1 HP (*n* = 249)	3 HP (*n* = 251)	*p*-value*
Hepatoxicity	13 (5.2%)	10 (4.0%)	0.67
Rash/urticaria	12 (4.8%)	0 (0.0%)	<0.01
Nausea/vomiting	8 (3.2%)	4 (1.6%)	0.38
Peripheral neuropathy	3 (1.2%)	0 (0.0%)	0.25
Hypersensitivity syndrome	2 (0.8%)	7 (2.8%)	0.18
Fever related to medication	0 (0.0%)	2 (0.8%)	0.50

*Two-sided Fisher’s exact *P.*

In subgroup analyses for safety, there was little difference between treatment groups by site, age, weight, or sex. Rates of a targeted safety event or treatment discontinuation due to side effects among those younger than the 40 years were similar (1HP 10.7%, 3HP 10.7%, RD = −0.03%, 95% CI: [−7.6, 7.6], *p*-value = 0.99), but differed among the older participants (1HP 21.7%, 3HP 10.0%, RD = 11.3%, 95% CI [2.4, 20.1], *p*-value 0.02; p-value for interaction = 0.13; Table 4).

## Discussion

In this pragmatic Phase 4 trial of two short-course TPT regimens, we found that both regimens had very high adherence with the four weeks of daily rifapentine and isoniazid having a slightly higher proportion of recipients who completed at least 90% of doses compared to three months of weekly rifapentine and isoniazid, with an adjusted difference of 5.3% (*p*-value 0.07). The higher rate of completion of TPT with 1HP is consistent with findings over many years which have demonstrated that the key factor in treatment completion is the duration of treatment [21]. Adherence to treatment for an asymptomatic condition is notoriously challenging. In studies of 1, 2, 3, and 4 months of TPT, completion of the shorter regimens was consistently superior to longer courses of isoniazid [8,9,12,14,19]. In the Prevent TB Trial, the pace of discontinuing 3HP for any reason was initially higher than that for isoniazid, but because the 9-month course of isoniazid continued for so long, overall rates of discontinuation were substantially higher than with 3HP [9]. It is therefore no surprise that completion of a 1-month regimen should be higher than for a 3-month regimen, despite a slightly higher incidence of adverse safety reactions.

Contrary to our expectations, the 1HP regimen was associated with a higher rate of targeted adverse safety events and treatment discontinuations than 3HP, even though more 1HP recipients were successfully treated. The RD of 6.1% for safety outcomes reflected a difference in both targeted safety events (adverse reactions known to be associated with rifapentine and isoniazid) and treatment discontinuation for any side effect. However, after adjustment for baseline covariates this difference was attenuated, with an adjusted RD of 3.4% (95% CI [−2.25, 9.1]). We found a higher rate of targeted adverse safety events for participants receiving 1HP in this trial than was reported in the BRIEF-TB trial, but we included Grade 2 or higher events, whereas BRIEF-TB only reported Grade 3 or higher events. There has been a growing interest in recording patient-centered outcomes in tuberculosis trials, and lower-grade toxicities may affect patient adherence and satisfaction [22,23]. The incidence of Grade 3 or higher adverse events with 1HP were, in fact, similar to that found for people with HIV in BRIEF-TB (3.6% versus 3.5%). Conversely, the 3HP regimen in this trial was better tolerated than in the Prevent TB trial, though rates of hepatotoxicity were higher (4.0% versus 0.4%). A larger proportion of participants receiving 1HP temporarily discontinued treatment (14/249, 5.6%) compared to 3HP (8/251, 3.2%), and it is possible that in real world settings some of these people may not have restarted treatment. Overall, however, successful treatment completion was excellent in both study arms.

Dramatic expansion of TPT is an essential component of the END TB Strategy and crucial for reducing global tuberculosis incidence and mortality [24,25]. Uptake of preventive therapy has been hampered by several factors, including the long duration of isoniazid therapy, high costs of rifapentine-containing regimens, and lack of generic products [26,27]. The availability of newer generic formulations of rifapentine, including fixed-dose combinations and dispersible tablets, should facilitate broader use of preventive therapy. Higher completion rates for shorter regimens offer an additional benefit, but uptake of 1HP has been limited by the lack of safety and tolerability data in people without HIV infection. Our study demonstrates that rates of treatment-limiting adverse safety reactions with 1HP in people without HIV are low and that completion is better than with 3HP. While the cost of 1HP is greater than 3HP because of the greater total dosage of rifapentine, the cost-effectiveness of both 1HP and 3HP has been demonstrated in populations with a high prevalence of HIV infection [28]; a cost-effectiveness analysis of the Ultra Curto Study is underway.

A strength of this trial is the use of objective pharmacologic assessments to evaluate regimen use. Both the collection of urine and whole blood as DBS are minimally invasive modalities to evaluate isoniazid and rifapentine adherence, respectively; while this approach has been used in HIV prevention studies, it is not regularly employed in tuberculosis studies [17,29]. Within this analysis, pharmacologic assessments distinguished differences in likely adherence between the 1HP and 3HP arms, with a larger reduction in confirmed adherence compared to self-report (1.4% for 1HP versus 4.3% for 3HP). A limitation of this study is reliance on self-report for assessment of overall adherence, although we supplemented this with pharmacologic monitoring at two time-points. Directly observed preventive therapy more reliably measures adherence but is impractical for public health scale-up and clinical practice [30,31]. In addition, this study did not attempt to compare the efficacy of 1HP and 3HP, as both have been shown to be non-inferior to standard of care isoniazid regimens and TPT regimens found to be efficacious in specific patient populations are generally accepted to be efficacious in others.

The World Health Organization has recommended 1HP as an alternative regimen for people other than those with HIV infection based on this principle, despite the absence of safety data [29]. This trial now provides those data and allows clinicians, public health programs, and patients to make informed choices about which regimens to use.

## Supporting information

S1 Fig Analytic Strategy and for Treatment Success with outcomes. Pharmacologic monitoring was performed at 2 visits. A positive result on either test at either visit was considered evidence of adherence. Abbreviations: HP, isoniazid and rifapentine; INH, isoniazid; RPT, rifapentine.
(TIF)

S2 Fig Rifapentine concentration versus time from last dose for recipients of 1HP (panel A) and 3HP (panel B). (TIF)

S1 Checklist Consort checklist. https://dx.doi.org/10.1136/bmj-2024-081123. This checklist is licensed under the Creative Commons Attribution 4.0 International License (CC BY 4.0; https://creativecommons.org/licenses/by/4.0/).
(DOCX)

S1 Text Ultra Curto Study Protocol Version 5.0. (PDF)

S2 Text Ultra Curto Statistical Analysis Plan Version 1.0. (PDF)

S3 Text List of protocol amendments. (DOCX)

## Abbreviations

CI: confidence interval
CV: coefficient of variation
DBS: dried blood spots
GEE: generalized estimating equations
HP: isoniazid and rifapentine
RD: risk difference
TPT: tuberculosis preventive therapy
