# Supplementary figures and images for: Acceptability and safety of one versus three months of rifapentine and isoniazid to prevent tuberculosis in people exposed in the household or workplace in Brazil: The Ultra-Curto randomized controlled trial
Source: PLoS Med. 2026 Feb 10;23(2):e1004758. doi: 10.1371/journal.pmed.1004758 (PMC12890141; doi:10.1371/journal.pmed.1004758)

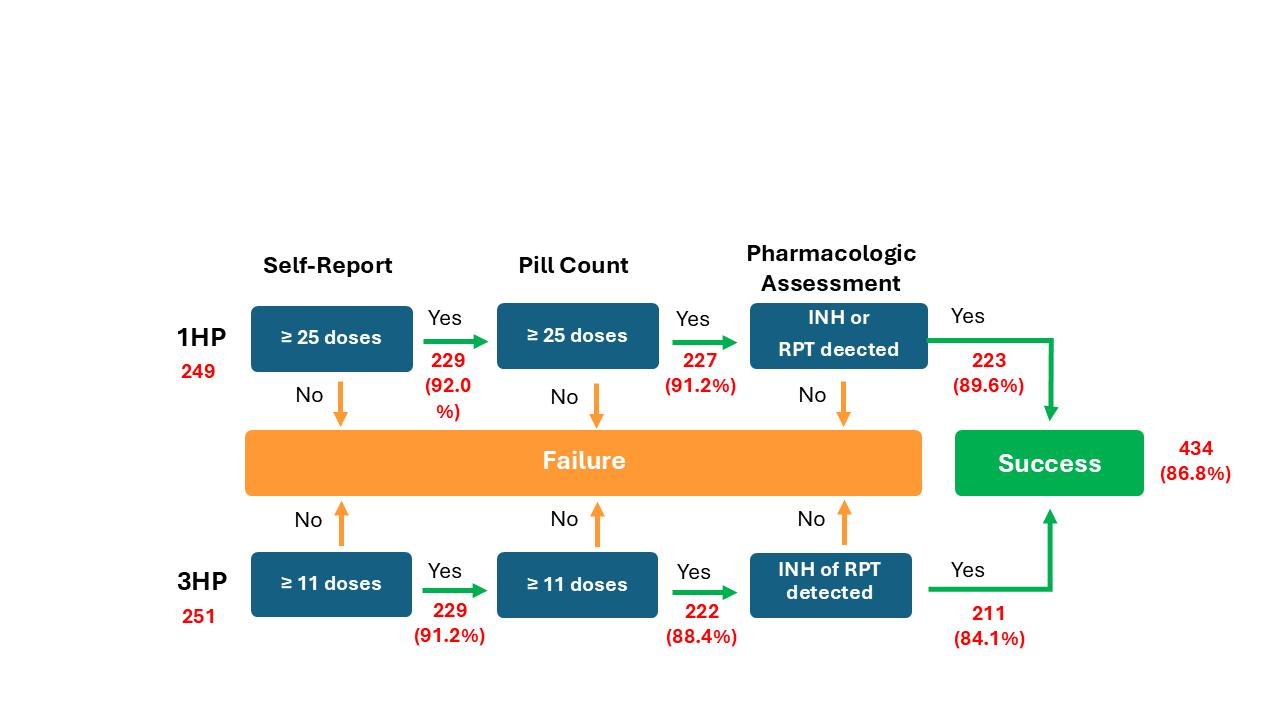

Supplement: S1 Fig — Pharmacologic monitoring was performed at 2 visits. A positive result on either test at either visit was considered evidence of adherence. Abbreviations: HP, isoniazid and rifapentine; INH, isoniazid; RPT, rifapentine. (TIF) [file pmed.1004758.s001.tif]

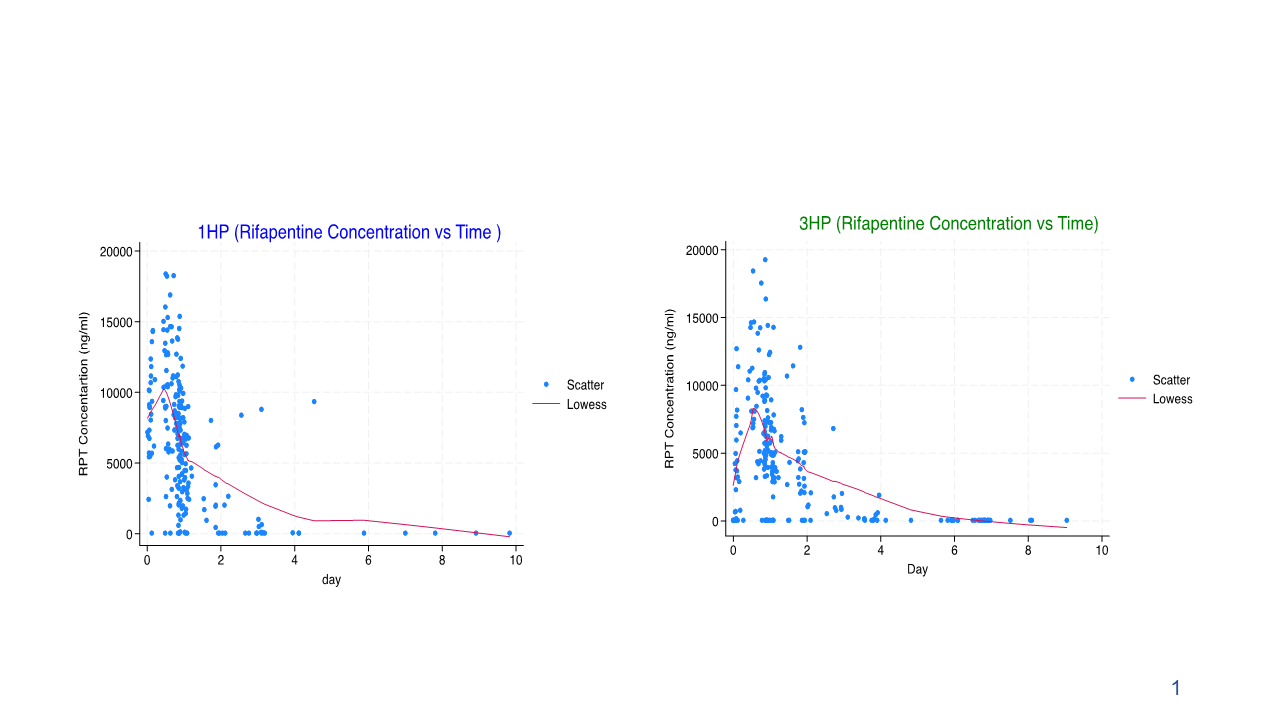

Supplement: S2 Fig — (TIF) [file pmed.1004758.s002.tif]
