# Supplementary material for: Acceptability and safety of one versus three months of rifapentine and isoniazid to prevent tuberculosis in people exposed in the household or workplace in Brazil: The Ultra-Curto randomized controlled trial
Source: PLoS Med. 2026 Feb 10;23(2):e1004758. doi: 10.1371/journal.pmed.1004758 (PMC12890141; doi:10.1371/journal.pmed.1004758)
Supplement: S1 Text — (PDF) [file pmed.1004758.s004.pdf]

1. TITLE PAGE

Full Title:
Treatment Success and Safety of 4 Weeks of Daily Rifapentine and Isoniazid (1HP) vs. 12 weeks of Weekly Rifapentine and Isoniazid (3HP) for Prevention of Tuberculosis in HIV-uninfected Individuals (1v3HP for TPT in HIV-uninfected individuals)

Short Title:
Ultra Curto TPT

Clinical Trial Phase:
Phase IV

Grant number U01AI152961 TB study in Brazil (DMID 20-0020)

**Protocol number:**	**U01AI152961**
**Short study name:**	**Ultra Curto TPT**
**Protocol Version:**	**5.0**
**Date:**	**4 July 2023**
**Sponsor:**	**JHU**
**Funded by:**	**NIH/DMID**

JHU IRB Identifier: 00284317

Pharmaceutical and other Study Product Support Provided by:
Sanofi (Paris, France)

Chair: Richard E. Chaisson, M.D.

DMID Medical Officer/Medical Monitor:

Francisco J. Leyva MD, PhD, ScM

2. SIGNATURE PAGE

I agree to conduct the study in accordance with the current protocol and will not make changes to the protocol without permission from DMID and CONEP.

I agree to personally conduct or supervise this study.

I will ensure that the requirements for obtaining the Informed Consent Form and approval by the National Research Ethics Committee (CONEP) or Research Ethics Committee (CEP) (Resolution 466 of 2012 of the National Brasilian Health Council and complementary resolutions) are attended to.

I agree to report adverse experiences that occur during this study to the sponsor.

I agree to maintain adequate and accurate study records and to make these records available for inspection or monitoring by relevant applicable regulatory bodies or at the discretion of the sponsor.

I also agree to immediately report to the CEP all study modifications and all problems and unforeseen events involving risks to humans or others. In addition, I will not make any changes to the study without the approval of CONEP / CEP and the study sponsor

I agree to ensure that all team members involved in conducting this study are informed of their obligations to fulfill the above commitments and maintain confidentiality.

Signature:

Date: 07/05/2023
Principal investigaror
BETINA DUROVNI

3. TABLE OF CONTENTS

Contents

1. TITLE PAGE ... 1
2. SIGNATURE PAGE ……………………………………………………………..……….. 2
3. TABLE OF CONTENTS ... 3
4. KEY ROLES ... 8
5. LIST OF ABBREVIATIONS ... 10
6. PROTOCOL SUMMARY ... 12
6.1 Study Schema ... 14
6.2 Schedule of Procedures/Evaluations ... 14
6.3 Study Duration ... 15
7. INTRODUCTION ... 18
7.1 Background Information ... 18
7.2 Rationale ... 18
8. OBJECTIVES and Hypotheses ... 18
8.1 Primary Objective ... 18
8.3 Secondary Outcomes ... 19
9. STUDY DESIGN ... 19
9.1 Study Groups ... 19
9.2 Dosing Structure ... 19
9.3 Trial Period ... 20
9.4 Site Descriptions ... 20
10. STUDY POPULATION ... 21
10.1 Inclusion/Exclusion Criteria ... 21
10.1.1 Participant Inclusion Criteria ... 21
10.1.2 Participant Exclusion Criteria ... 21
10.2 Recruitment Process ... 22
10.3 Participant Retention ... 26
11. INTERVENTIONS ... 26
11.1 Biomedical Interventions ... 26
11.1.1 Regimen ... 26
11.1.2 Study Product Formulation and Preparation Rifapentine (source: study 31 v.2.0) ... 27
11.1.3 Study Product Supply and Accountability ... 270
11.1.4 Assessment of Participant Adherence with Study Product(s)/Intervention(s) ……...31
11.1.5 Concomitant Medications and Procedures ... 32

11.1.6 Permitted Medications and Procedures 33
11.1.7 Prohibited Medications and Procedures 33
11.1.8 Precautionary Medications and Procedures 30
11.1.9 Required Medications 31
11.1.10 Rescue Medications 31
12. STUDY PROCEDURES/EVALUATIONS 314
12.1 Clinical Evaluations and Procedures 314
12.1.1 Screening 314
12.1.2 HIV Counseling and Testing 325
12.1.3 Enrollment 336
12.1.4 Follow-up visits 347
12.1.5 Treatment Completion and Successful Treatment 43
12.2 Laboratory Evaluations 403
12.2.1 Specimen Preparation, Handling and Shipping 403
12.2.2 Total Blood Volume 404
12.3 Other Study Procedures and Evaluations 415
12.3.1 Early Termination Visit 415
12.3.2 Management of a participant who becomes pregnant during the study 426
12.3.3 Management of participants who are discontinued from study treatment in the setting of an adverse event or the investigator judges that discontinuation of study treatment is in the participant's best interest 426
12.3.4 Management of a participant who requests premature discontinuation from study treatment 42
6
12.3.5 Management of a participant who is incarcerated after enrollment 437
12.3.6 Post Early Termination Visit (Visit after Early Termination) for Participants Terminating before Completion of their Assigned Study Treatment 47
12.3.7 Early Termination Visit for Participants Stopping Study Participation after Completion of their Assigned Study Treatment 47
12.3.8 Missed Visit 48
12.3.9 Unscheduled Visit 48
12.3.10 Final Study Visit 48
12.3.11 Time-And-Motion (TAM) Studies 48
13. SAFETY ASSESSMENT 49
13.1 Safety Assessment Overview 49
13.2 Adverse Event Procedures and Reporting Requirements 50
13.2.1 Adverse Events 50

13.2.2 Study defined AEs 50
13.3.3 Serious Adverse Events 50
13.3.4 Adverse event (AE) reporting 51
13.3.5 AEs requiring expedited reporting 51
13.3.6 AE Reporting period 51
13.3.7 Recording updated information on SAEs 51
13.3.8 Recurrent SAEs 52
13.3.9 Timeframe for SAE reporting 52
13.3.10 Site investigator assessment and signature 52
14. CLINICAL MANAGEMENT (Source 3HP DTG v5.0) 483
14.1 Clinical Management of Adverse Events 483
14.1.1 Toxicity grading tables 483
14.2 Toxicity management 483
14.2.1 Gastrointestinal 483
14.2.2 Cutaneous 53
14.2.3 Rifamycin hypersensitivity syndrome (RHS) (Source 3HP DTG v5.0) 493
14.2.4 Drug-Associated Fever 54
14.2.5. Liver Toxicity 55
14.2.6 Peripheral Neuropathy 505
14.2.7 Other Toxicities 516
14.3 Pregnancy 516
14.4 Criteria for Discontinuation 516
14.4.2 Premature study discontinuation 527
15. STATISTICAL CONSIDERATIONS 57
15.1 Overview and General Design Issues 57
15.2 Study Outcomes 58
15.2.1 Primary Outcome 58
15.2.2 Secondary Outcome 58
15.3 Study Hypotheses 58
15.4 Sample Size Considerations 59
15.5 Enrollment/Stratification/Randomization/Blinding Procedures/Unblinding Procedures 60
15.6 Maintaining Trial Treatment Randomization Codes 61
15.7 Participant Enrollment and Follow-up 61
15.8 Data and Safety Monitoring 61
15.8.1 Planned Interim Analysis and Stopping Guidelines 61
15.8.2 Interim Efficacy Review 62

15.8.3 Analysis Plan 62
15.8.4 Economic Analysis Plan…….62
16. DATA HANDLING AND RECORDKEEPING 573
16.1 Data Management Responsibilities........ 573
16.2 Essential/Source Documents and Access to Source Data/Documents........ 63
16.3 Quality Control and Quality Assurance........ 584
17. CLINICAL SITE MONITORING........ 584
18. HUMAN SUBJECTS PROTECTIONS 605
18.1 Institutional Review Board/Ethics Committee 605
18.2 Vulnerable Participants........ 605
18.2.1 Pregnant participants and fetuses........ 605
18.2.2 Prisoners........ 605
18.2.3 Children 605
18.2.4 Illiterate participants 606
18.3 Informed Consent 616
18.3.1 Informed Consent Process 616
18.3.2 Assent Process 627
18.3.3 Documenting Informed Consent........ 627
18.3.4 Stored samples 68
18.4 Risks and SARS-CoV-2 Precautions…….68
18.5 Social Impact Events........ 70
18.6 Benefits........ 70
18.7 Compensation 70
18.8 Participant Privacy and Confidentiality........ 71
18.9 Certificates of Confidentiality........ 71
18.10 Critical Event Reporting 71
Critical event reporting is described in Section ______ 71
18.11 Communicable Disease Reporting........ 71
18.12 New Findings........ 71
18.13 Study Discontinuation........ 71
18.14 Post-Trial Access 72
18.15 Community Advisory Board and Other Relevant Stakeholders........ 72
19. ADMINISTRATIVE PROCEDURES 72
19.1 Protocol Registration 72
19.2 Regulatory Oversight 72

19.3 Study Implementation 72
19.4 ClinicalTrials.gov 673
20. PUBLICATION POLICY 673
21. REFERENCES 673
22. APPENDICES 728
I Schedule of events and study duration 729
II Sample study medication & food diary 82
III Rifapentine hypersensitivity reaction worksheet 84
IV List of Precautionary Medications 85
V *DAIDS Table for Grading Adult and Pediatric Adverse Events, Version 2.1* (March 2017) ... 87

4. KEY ROLES

Principal Investigator
Richard E. Chaisson, MD
Division of Infectious Diseases
Johns Hopkins University School of Medicine
1550 Orleans Street, Room 1M.08
Baltimore, MD 21287
Phone: +1-443-955-1755
Email: rchaiss@jhmi.edu

NIH Program Officer
Barbara E. Laughon, PhD
Senior Scientist for TB Drug Development Partnerships
Tuberculosis Research Section, Respiratory Diseases Branch
Division of Microbiology and Infectious Diseases, NIAID, NIH
5601 Fishers Lane, Room 7G78
Bethesda, MD 20892-9826
Tel: 240.627.3302
Fax: 240.627.3114
BLaughon@niaid.nih.gov

NIH Medical Officer
Francisco J. Leyva MD, PhD, ScM
Medical Officer
Respiratory Diseases Branch - DMID
NIAID - NIH - HHS
5601 Fishers Ln., Room 8E27
Rockville MD 20852-9825
francisco.leyva@nih.gov

Site Principal Investigators
Investigators
Marcelo Cordeiro-Santos, MD, Msc, PhD
Head of the Clinical Research Center on Mycobacteria - Fundação de Medicina Tropical Dr. Heitor Vieira Dourado (FMT-HVD)
Associate Professor – Universidade do Estado do Amazonas (UEA), Av. Pedro Teixeira, 25 - Manaus - Amazonas - Brasil - 69040-000
Phone: +55 92 99119 9199
E-mail: marcelocordeiro.br@gmail.com

Solange Cesar Cavalcante, MD, MSc, PhD
Senior Researcher - Fundação Oswaldo Cruz, Rio de Janeiro
Infectious Disease Specialist- Municipal Health Secretariat of Rio de Janeiro
Phone: 5521 996318789
Email:drsocavalcante@yahoo.com.br

Betina Durovni, MD, MPH, PhD
Infectious Disease Specialist- Municipal Health Secretariat of Rio de Janeiro
Senior Researcher - Center for Strategic Studies - Fundação Oswaldo Cruz, Rio de Janeiro
Phone: 5521 999943122
E-mail: bdurovni@gmail.com

Statistician

Larry Moulton, PhD
Global Disease Epidemiology and Control, Johns Hopkins Bloomberg School of Public Health
Wolfe St, Room E5519
Phone: (410) 955-6370
E-mail: larrymoulton@gmail.com

Data Manager

Silvia Cohn, MPH, MSc
Johns Hopkins Center for TB Research
Johns Hopkins University School of Medicine, 1550 Orleans St, 1M.02
Baltimore, MD 21287
Phone: (410) 955-1755
Email: scohn5@jhmi.edu

Pharmacology Laboratory Director

Mark Marzinke, PhD, DABCC
Director, Clinical Pharmacology Analytical Laboratory (CPAL)
Division of Clinical Pharmacology
Johns Hopkins University
4940 Eastern Avenue
Mason F. Lord Center Tower
Baltimore, MD 21224
TEL: 410-550-9704
FAX: 410-502-5598
Email: mmarzin1@jhmi.edu

Coordenador Central de Estudos

Beatriz Kohler, RN, MPH, MBA
Center for Tuberculosis Research
Escola de Medicina da Universidade Johns Hopkins
1550 Orleans Street, Suite 1M.06
Baltimore, MD 21287
Phone: +1 410-614-3812
Email:

Consultant

Susan Swindells, MBBS
Infectious Diseases/Internal Medicine
University of Nebraska Medical Center
988106 Nebraska Medical Center
Omaha, NE 68198-8106
Phone: (402) 559-5392
FAX: (402) 553-5527
Email: sswindells@unmc.edu

Epidemiologists

David Dowdy, MD, PhD
Johns Hopkins University
Bloomberg School of Public Health
615 N. Wolfe Street, Room E6531
Baltimore, MD 21205
Email: ddowdy1@jhmi.edu

Nicole Salazar-Austin, MD, ScM
Johns Hopkins School of Medicine
Bloomberg Children's Center
200 N. Wolfe St.
Baltimore, MD 21287
Nsalaza1@jhmi.edu

5. LIST OF ABBREVIATIONS

AE/AER/EAE	Adverse Event / Adverse Event Report / Expedited Adverse Event
AFB	Acid Fast Bacilli
AIDS	Acquired Immunodeficiency Syndrome
ALT/SGPT	Alanine Aminotransferase
ART/ARV	Antiretroviral Therapy / Antiretroviral
AST/SGOT	Aspartate Aminotransferase
AUC	Area Under the time-concentration Curve
CBC	Complete Blood Count
CDC	(US) Centers for Disease Control and Prevention
Cmax	Maximum concentration
CRF	Case Report Form
CXR	Chest X-ray
DBS	Dried Blood Spot
DOT	Directly Observed Therapy
DR-TB	Drug-Resistant Tuberculosis
DS-TB	Drug-Susceptible Tuberculosis/Drug-Sensitive Tuberculosis
DSMB	Data and Safety Monitoring Board
DST	Drug Susceptibility Testing
EC	Ethics Committee
E/CIA	Chemiluminescence immunoassay
FDA	(US) Food and Drug Administration
H/INH	Isoniazid
HBSAG	Hepatitis B Surface Antigen
HHC	Household contact
HIV	Human Immunodeficiency Virus
HP	Isoniazid and Rifapentine
IoR	Investigator of Record
IPT	Isoniazid Preventive Therapy
IRB	Institutional Review Board
LFT(s)	Liver Function Test(s)
LTBI	Latent Tuberculosis Infection
MDR-TB	Multidrug-Resistant Tuberculosis
MIC	Minimum Inhibitory Concentration
MOP	Manual of Operations
M.tb.	*Mycobacterium tuberculosis*
P/RPT	Rifapentine
PI	Principal Investigator
PK	Pharmacokinetics
RIF	Rifampin/Rifampicin
SAE	Serious Adverse Event
SOC	Standard Of Care
SOE	Schedule of Evaluations
SOP	Standard Operating Procedure

T1/2	Half-life
TB	Tuberculosis
TBTC	Tuberculosis Trials Consortium
TPT	TB Preventive Therapy
ULN	Upper Limit of Normal
WHO	World Health Organization
XDR-TB	Extensively Drug Resistant Tuberculosis

6. PROTOCOL SUMMARY

**Protocol Title:**	Treatment Success and Safety of 4 Weeks of Daily Rifapentine and Isoniazid (1HP) vs. 12 weeks of Weekly Rifapentine and Isoniazid (3HP) for Prevention of Tuberculosis in HIV-uninfected Individuals (1v3HP for TPT in HIV-uninfected individuals): Ultra Curto
**Treatment Indication:**	TB preventive treatment (TPT) in HIV uninfected persons age ≥15 years with LTBI
**Trial Objective:**	To compare treatment success (adherence and completion of treatment) and safety of 1HP with 3HP in HIV-uninfected adults and adolescents at increased risk of TB.
**Trial Design:**	An international, multicenter, randomized, controlled, open-label, two arm, Phase IV clinical trial.<br><br>Arm A (n=250): Experimental arm. Rifapentine 600 mg daily and isoniazid 300 mg daily for 4 weeks.<br><br>Arm B (n=250): Control arm. Rifapentine 900 mg and isoniazid 900 mg weekly for 12 weeks.
**Sample Size**	500
**Patient Population:**	The study will enroll HIV-uninfected individuals who are ≥ 15 years old, and are candidates to receive TB preventive therapy (i.e. LTBI with no evidence of active TB). Key exclusion criteria include HIV infection, abnormal liver enzymes, neutropenia, previous treatment of TB or latent TB infection, or a history of study drug intolerance.
**Study treatment:**	TB preventive treatment:<br>Arm A (n=250): Experimental arm. Rifapentine 600 mg daily and isoniazid 300 mg daily for 4 weeks.<br><br>Arm B (n=250): Control arm. Rifapentine 900 mg weekly and isoniazid 900 mg weekly for 12 weeks.
**Trial sites:**	• Health units from Rio de Janeiro Health Secretariat and NAPDOT study clinic, Rio de Janeiro, Brazil<br>• Fundação de Medicina Tropical (FMT) Dr. Heitor Vieira Dourado; Manaus, Amazonas State, Brazil

Criteria for evaluation:

Primary Objectives:

To compare treatment success (completion of treatment with ≥90% adherence) of 1HP compared with 3HP in HIV-uninfected adults and adolescents at increased risk of TB.

To compare the safety of 1HP vs 3HP in HIV-uninfected adults and adolescents at increased risk of TB.

Primary Outcomes:

(1) Successful completion of TPT with ≥90% adherence documented by self-report, pill count, and pharmacologic monitoring.

(2) Safety of treatment regimens, defined as occurrence of Grade 2 or higher targeted safety events while taking study medications or within 14 days of stopping, or discontinuation of study medications because of side effects.

Targeted safety events are hypersensitivity syndrome, rash, peripheral neuropathy, hepatotoxicity, nausea and vomiting, and drug-related fever.

Secondary Outcome:

(1) Incremental cost-effectiveness of 1HP and 3HP (compared to each other, 6H, and no treatment) using a societal perspective.
(2) Analyze, in each of the health units in the study, the time needed to provide comprehensive care to patients.

6.1 Study Schema

Figure 1

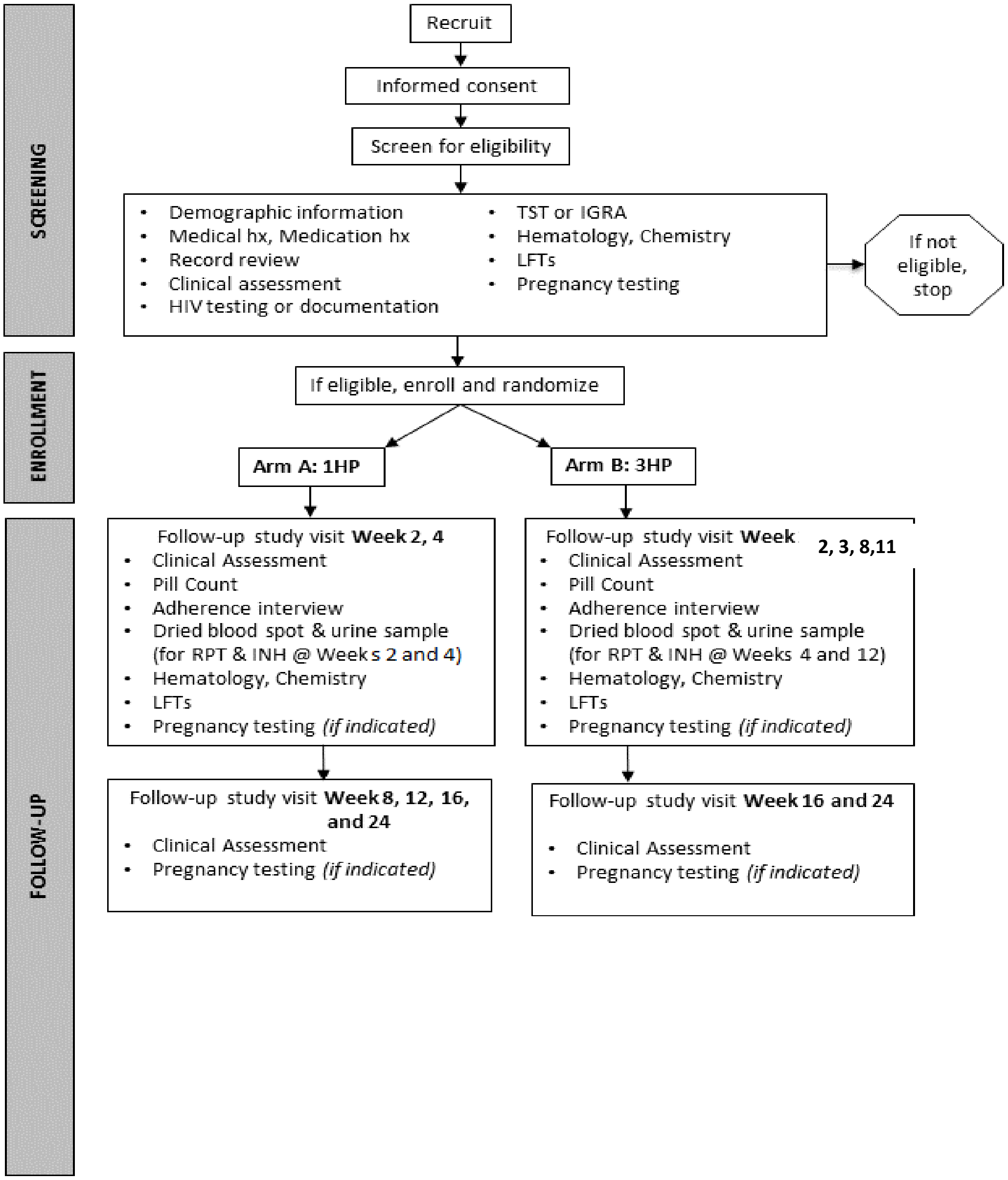

6.2 Schedule of Procedures/Evaluations

See APPENDIX I

6.2.1 Laboratory Evaluations

Screening and follow-up

Hematology
Hemoglobin, hematocrit, red blood cells, white blood cell count, and platelets.

Blood Chemistry
Sodium, potassium, chloride, creatinine, and albumin.

Liver Function Tests
Total bilirubin, AST (SGOT), ALT (SGPT), and alkaline phosphatase.

These tests should be performed at any time during the treatment and post-treatment phase if the participant exhibits signs suggestive of hepatitis (e.g., fatigue, weakness, malaise, anorexia, nausea, vomiting, abdominal pain, pale stools, dark urine, chills, or jaundice).

Pregnancy Test
Serum or urine β-HCG

Dried Blood Spot collection and temporary storage / batch testing
Dried blood spot collection for presence of rifapentine

Urine collection
Urine collection and point of care testing for presence of isoniazid

Sampling for NAT2 metabolizer genotype
Dried blood spot or urine as determined by local capacity

Additional evaluations at Screening only

HBsAg
HIV-1 antibody test
TST or IGRA

6.3 Study duration is in APPENDIX I

7. INTRODUCTION

7.1 Background Information

Tuberculosis (TB) is the leading infectious killer globally and a major cause of illness and suffering. The World Health Organization has prioritized TB preventive therapy (TPT) for people with latent TB infection (LTBI) as a key strategy for controlling the epidemic. Prevention of TB with isoniazid preventive therapy (IPT) is effective and reduces morbidity and mortality, and has been the mainstay of TB prevention for decades. But for an intervention with an excellent evidence of efficacy, global uptake has been abysmal. Completion rates for IPT when it is administered are poor (Gillespie 2008; Durovni 2010), with a large proportion of patients unable to complete treatment (McClintock 2017; Sterling 2011). While uptake is influenced by a variety of factors, a critical element has been the duration of IPT, with adherence falling sharply over time in clinical trials and practice. Shorter course regimens have a much higher completion rate and are more acceptable to patients, clinicians, and programs.

7.2 Rationale

With support from the NIH and the CDC, we have pioneered the development of short-course, rifamycin-based TB preventive therapy (TPT) over the past 15 years. We have evaluated the efficacy of 3 months of weekly rifapentine and isoniazid (3HP) in people with and without HIV infection and showed that it is non-inferior to longer courses of INH, with better adherence and less toxicity. (Schechter 2006; Martinson 2011; Sterling 2011)

This regimen is now recommended as a first-line treatment for latent TB infection by the CDC and the World Health Organization, offering the potential of substantially increased uptake of TPT as part of the END TB Strategy. (WHO 2018)

More recently, supported by NIAID, we have shown that one month of daily rifapentine and isoniazid (1HP) is non-inferior to nine months of INH in people with HIV infection. (Swindells 2019) The availability of two innovative, new short-course TPT regimens offers a transformative opportunity to global TB control and achieving the End TB targets. However, a major question still requiring evidence is the safety of the 1HP regimen in people without HIV infection.

While the efficacy of 1HP can be assumed from its success in HIV-positive people, safety cannot. In the 1990s, several studies supported by NIAID and the CDC showed that a 2-month TBT regimen of rifampin and pyrazinamide (2RZ) was safe and efficacious in HIV-infected individuals.(Halsey 1998; Gordin 2000) However, subsequent research funded by NIAID, and experience in clinical practice, demonstrated that 2RZ was not

safe in HIV-uninfected people, with high rates of hepatotoxicity and a number of deaths.(Schechter 2006; CDC 2001) No explanation for the difference in safety in HIV-infected and –uninfected people has ever been found, but this experience underscores the importance of demonstrating safety and tolerability in all target populations of new regimens for TBT.

While the 3HP regimen has been proved safe and effective in HIV-positive and –negative people, 1HP has only been shown to be safe and efficacious in HIV-positive people. An efficacy study in HIV-negatives would be useful for purposes of replication of the single study demonstrating the non-inferiority of 1HP, but such a trial would be extremely costly and time -consuming. The ACTG BRIEF TB trial took 6 years at a cost of >$30 million with its sample size of 3000. It is likely that a non-inferiority efficacy trial in HIV negatives would require a sample size of at least 4500.

In the history of TB preventive therapy, a regimen that is efficacious in one population has always been efficacious in others. For 3HP, efficacy was found to be quite similar in HIV+ people and HIV-negative, as well as in children. What can differ, however, is toxicity and tolerability. As shown with the 2-month rifampin and pyrazinamide (2RZ) regimen, safety in people with HIV does not ensure safety in HIV-negative people. Thus, a safety and adherence study is both necessary and preferred.
1HP is now recommended by the WHO as a first-line regimen for treating latent TB infection in individuals >12 years of age. (WHO, 2020) To date, this regimen has only been studied in people with HIV infection. Because no formal studies of HIV seronegative people have been conducted, this trial will provide important information on the safety of the regimen in this population.

Because the 1HP regimen was developed exclusively with NIAID funding and sponsorship, the opportunity for the NIH to have full responsibility and credit for introducing a highly novel, short-course preventive therapy regimen that could radically transform TB control is at hand. A trial showing that 1HP is safe, tolerable, and more likely to be completed than 3HP has the potential to fundamentally alter TB control interventions worldwide.

Rationale for self-administered therapy

Directly observed therapy (DOT) has been the standard of care for treatment of active TB in clinical trials and clinical practice for many years. Based on WHO guidance that intermittent treatment using rifamycins should be done under DOT, the original trials of 3HP provided all treatment under supervision of a health care team member. (Schechter, Martinson, Sterling) Subsequently, it became apparent that DOT for TPT was not economically or clinically feasible in most settings outside the US, and a trial comparing self-administered with DOT 3HP was conducted by the TB Trials Consortium. The i-Adhere Study compared treatment completion of 3HP in individuals receiving TPT either by DOT or by self-administration, with or without SMS

reminders. The study was a non-inferiority trial with an NI margin of 15% for treatment completion. Adherence in the self-administered arms was measured by electronic pill caps. In the primary analysis, self-administered 3HP failed to show non-inferiority to DOT, with rates of 87.2% completion for DOT vs. 75% (lower 95% CI 68.9%) for self-administered and 76.4% (lower 95% CI 71.3) for self-administered with SMS reminders. In the pre-planned analysis of participants enrolled in the US, however, non-inferiority of self-administration was demonstrated. On the basis of these results, the CDC now endorses self-administration of 3HP as a treatment option, and the WHO guidelines likewise support self-administration. The BRIEF-TB study used self-administered 1HP and showed a high treatment completion rate (97%) and excellent adherence. Based on global guidelines and the impracticality of using DOT for the millions of patients who require TPT, we will give both 1HP and 3HP by self-administration.

Rationale for including adolescents

Inclusion of children in clinical trials of tuberculosis treatment increasingly has been called for to support the rational use and increased availability of anti-tuberculosis medications for children (Burman 2008, McKenna 2014). Tuberculosis disease characteristics, presentation, diagnosis, and treatment are similar for adults and adolescents. Rifapentine currently is approved as TPT in the United States for use in persons as young as 2 years old (Priftin® package insert 2018). Rifapentine pharmacokinetic results have been found to be similar between adults and adolescents down to age 12 (Marshall 1999). Rifapentine 900 mg once weekly, with isoniazid for 12 weeks has been used to treat children for LTBI (Villarino 2015): the rifapentine-containing regimen was found to be as safe and effective as a 9-month daily isoniazid regimen, among 552 children age 2-17 years old treated with the rifapentine-containing regimen. Daily dosing of rifapentine in children <12 has not been studied, however; the IMPAACT network is currently planning a trial of 1HP in children. Inclusion of adolescents is, therefore, appropriate, but inclusion of children <12 is not.

8. OBJECTIVES and HYPOTHESES

8.1 Primary Objectives

To compare treatment success (completion of treatment with ≥90% adherence) of 1HP with 3HP in HIV-uninfected adults and adolescents at increased risk of TB.

To compare the safety of 1HP with 3HP in HIV-uninfected adults and adolescents at increased risk of TB.

8.2 Primary Outcomes and Hypotheses

(1) Successful completion of TBT with ≥90% adherence documented by self-report, pill count, and pharmacologic monitoring.

Hypothesis: Successful treatment with 1HP will be superior to 3HP, with 90% of individuals randomized to 1HP taking >90% of prescribed doses vs 80% of those on 3HP.

(2) Safety of treatment regimens, defined as occurrence of Grade 2 or higher targeted safety events while taking study medications or within 14 days of stopping, or discontinuation of study medications because of side effects.Targeted safety events are hypersensitivity syndrome, rash, peripheral neuropathy, hepatotoxicity, nausea and vomiting, and drug-related fever. Hypothesis: We hypothesize that the safety of 1HP will be superior to 3HP, with a rate of targeted adverse events or medication discontinuation of 6% vs. 13% for 3HP.

8.3 Secondary Outcome and Hypothesis

(1) To compare the cost-effectiveness of 1HP and 3HP using a societal approach, modeling the incremental cost-effectiveness of 1HP vs 3HP, 6H, and no treatment.
(2) Analyze, in each of the health units in the study, the time needed to provide comprehensive care to patients.

Hypothesis: We hypothesize that 1HP will be cost saving vs 3HP, v modelled costs of 6H and v no TPT.

A separate protocol will be prepared for this component of the study.

9. STUDY DESIGN

This is a multicenter, randomized, controlled, open-label, phase IV clinical trial with two arms, comparing treatment success and safety of 1HP vs 3HP in HIV-uninfected adolescents and adults.

9.1 Study Groups

Arm A (n=250): Experimental arm
Rifapentine and isoniazid once daily for 4 weeks, 28 doses

Arm B (n=250): Control arm
Rifapentine and isoniazid once weekly for 12 weeks, 12 doses

9.2 Dosing Structure

Dosing of rifapentine and INH will be based on the dosages given to individuals >50

kg in the BRIEF TB trial for arm A and current CDC guidelines for arm B. Based on a meta-analysis of the pharmacokinetics of rifapentine in nine clinical trials of preventive therapy conducted by Hibma and colleagues (Hibma, 2020), weight-banded dosing will not be used. This analysis demonstrated that weight-banded dosing results in sub-optimal exposures for lower weight patients, as drug clearance is not proportional to body weight. An ongoing trial of rifapentine for the treatment of active TB is currently underway and is using flat dosing of rifapentine at 1200 mg/day (TBTC Study 31/ACTG A5349) and no signals of toxicity have been reported with this dosage in lower weight individuals. In 2020, the WHO revised its guidelines for treating latent TB and recommended flat dosing of rifapentine for adolescents and adults, regardless of weight. (WHO)

Arm A
Rifapentine: 600 mg once daily for 4 weeks (28 doses)
Isoniazid: 300 mg once daily for 4 weeks (28 doses)

Arm B
Rifapentine: 900 mg once weekly for 12 weeks (12 doses)
Isoniazid: 900 mg once weekly for 12 weeks (12 doses)

9.3 Trial Period

The study will take place over a five-year period. We dedicate the first half-year to regulatory approvals, site preparation, and training, then will initiate participant enrollment in the second half of year 1. Enrollment and follow up continues into the fourth year, followed by data analysis, abstract and manuscript preparation, and the study's final report in year five.

Each participant completes 24 weeks on study.

9.4 Site Descriptions

Rio de Janeiro Municipal Health Department

The Rio de Janeiro Municipal Health Department has an extensive network of primary care units that are responsible for caring for tuberculosis patients and their contacts. Primary care clinics in the AP 2.1 region of Rio de Janeiro, adjacent to a large *favela* with high rates of TB, will serve as feeder clinics for recruitment. The study team has extensive experience working in these clinics and has conducted previous clinical trials of TB interventions. Clinical activities for the trial will take place at the NAPDOT clinic, located in a public building adjacent to the entrance to the *favela.* The NAPDOT facility was initially established as part of the Gates Foundation-funded THRio Study and has served as a research site for subsequent NIH- and CDC- funded studies. It is equipped for active and latent tuberculosis diagnoses, with four exam rooms, a patient education area, waiting room, research offices, and a secure file room for medical and research records.

Fundação de Medicina Tropical (FMT) Dr. Heitor Vieira Dourado and Amazonas State University
Located in Manaus Amazonas State, is a modern research institute addressing the most important public health challenges for the people of the equatorial jungle. Areas of expertise include malaria, dengue, Zika, TB, HIV, and pharmacology. The unit collaborates with the State Health Department and provides specialty care for TB, HIV, severe malaria, leptospirosis, leishmaniasis, and other tropical infections. The campus includes an inpatient unit with 250 beds (including 20 ICU beds) for adults and children with serious infections, outpatient clinics, and clinical laboratories with BSL-3 capability for *M. tuberculosis*¸ and biorepository facilities.

10. STUDY POPULATION

The study will enroll HIV-uninfected individuals who are ≥ 15 years old, weight ≥ 40 kg, and are candidates to receive TB preventive therapy (i.e. LTBI with no evidence of active TB). The primary target population will be household contacts of newly diagnosed TB patients, recruited from primary care clinics.

10.1 Inclusion/Exclusion Criteria

10.1.1 Participant Inclusion Criteria

1) Positive tuberculin skin test or interferon-gamma release assay (IGRA) test <u>and</u>

2) Household contact of an infectious TB case within previous 90 days, defined as sleeping at least once in a residence with a person diagnosed with pulmonary TB, <u>or</u>

3) Documented conversion of TST/IGRA from negative to positive within 2 years

10.1.2 Participant Exclusion Criteria

1) Documented HIV infection

2) Evidence of active tuberculosis on clinical exam or chest x-ray

3) Known intolerance of any study drug

4) Treatment for active or latent TB in the past for more than 14 days

5) Known close contact to someone with INH or rifampin resistant TB

6) Active liver disease or AST/ALT >3 times ULN

7) Neutropenia (ANC <1000)

8) Peripheral neuropathy >Grade 1 by DAIDS Grading Table

9) Pregnant or breastfeeding. Women of childbearing potential must agree to use non-hormonal contraception during study treatment.

10) Weight <40 kg

11) At risk of poor outcome based on clinical judgment and discretion of investigator.

12) Required use of a prohibited medication with a serious drug-drug interaction with rifamycins or isoniazid.

10.2 Recruitment Process

Rio de Janeiro

Participants will be recruited from 3 primary health care (PHC) units located in Rocinha, a community located in the Municipal Planning Area 2.1 in the city of Rio de Janeiro.

Key steps in the recruitment process include:

- Health care workers from the 3 PHC units will evaluate the household contacts from pulmonary TB cases clinic to exclude active TB and evaluate/diagnosis LTBI.
- The PHC unit´s teams will provide initial information about the study and refer those presenting a positive TST or IGRA that express interest in the study to the Research Clinical Centre located in one of the 3 clinics (CF Rinaldo Delamare).
- The research team will receive the potentially eligible participants and evaluate for inclusion/exclusion criteria.
- For those eligible patients informed consent will be obtained by the study nurse.

Manaus

Recruitment will take place at the FMT-HVD ambulatory and hospital unit, where an active search of index cases and their close contacts will be carried out through the review of medical records and microbiological results.

Referrals from public clinics in Manaus will also be recruited following procedures used in Rio de Janeiro.

Key steps in the recruitment process include:

- Household contacts will be evaluated at the FMT-HVD research clinic.
- Samples will be taken soon after local inclusion procedures, as well as chest X-ray.
- All follow-up will be performed at the clinical research clinic.
- The research team will receive the potentially eligible participants and evaluate for inclusion/exclusion criteria.
- For those eligible patients informed consent will be obtained by the

study nurse.

10.3 Participant Retention

Recruiters and study staff will collect detailed locator information on all participants. Staff will test participant mobile and landline phone numbers at the screening visit and confirm contact information at every study visit.

Staff will teach participants when and how to notify study staff with questions or concerns, to report potential medication side effects and TB symptoms. Staff will coordinate unscheduled visits or referral to urgent care to manage signs and symptoms.

Staff will call participants who miss a study visit, or call their nominated contact if the participant is not reachable, to schedule a follow-up visit. The team may conduct a home visit, when necessary and feasible to retain the participant on-study.

Study staff will make reminder calls prior to study visits, notifying participants of the day/time of the visit, what to bring with them, and any other instructions.

11. INTERVENTIONS

11.1 Biomedical Interventions

11.1.1 Regimen

**Treatment Group**	**Regimen**<br>Dose, schedule, route, administration	**Duration**
**ARM A**	Rifapentine: 600 mg po once daily<br>Isoniazid: 300 mg po once daily<br><br>All medications will be self-administered	4 weeks<br>(28 doses)
**ARM B**	Rifapentine: 900 mg po once weekly<br>Isoniazid: 900 mg po once weekly<br><br>All medications will be self-administered	12 weeks<br>(12 doses)

11.1.2 Study Product Formulation and Preparation

Rifapentine

Rifapentine is a semisynthetic rifamycin derivative with a microbiologic profile similar to that of rifampin. Its structure differs from that of rifampin by the presence of a cyclopentyl ring instead of a methyl group at the piperazinyl moiety. It has a longer half-life than rifampin, and, like rifampin, rifapentine inhibits bacterial RNA synthesis by binding to the β-subunit of

DNA-dependent RNA polymerase.

Rifapentine is well absorbed from the gastrointestinal tract, with 70% bioavailability; when taken with food, its C_{max} and AUC increase by 40% - 50% (Priftin package insert 2018). It reaches peak concentrations in the serum 5 to 6 hours after ingestion. Rifapentine and its 25-desacetyl metabolite are highly protein-bound, 97.7% and 93%, respectively, primarily to albumin. Rifapentine is metabolized by an esterase enzyme found in the liver and blood to 25-desacetylrifapentine, a microbiologically active metabolite that contributes about 40% of the drug's overall activity. For *M. tuberculosis*, the MIC of 25-desacetyl rifapentine is 0.25 mcg/mL, while that of rifapentine is 0.05 mcg/ml. The drug and the active metabolite have half-lives of 14-17 and 13 hours, respectively.

The drug is excreted in bile and eliminated in feces. Less than 10% of rifapentine is excreted in the urine as unchanged drug. Rifapentine, like other rifamycins, induces CYP3A4, 2C8, and 2C9, which can lead to more rapid metabolism and clearance of many drugs. Rifamycins are also known to induce the activity of phase II enzymes such as glucuronosyltransferase and sulphotransferase and may reduce levels of drugs metabolized by those pathways.

Rifapentine (Priftin, Sanofi) is available as 150 mg tablets. Rifapentine, like other rifamycins, causes red-orange discoloration of body fluids and can stain contact lenses. In clinical trials in which rifapentine was combined with isoniazid and other antituberculosis drugs and administered once or twice weekly, rates of adverse reactions were similar with rifampin and rifapentine, with increased liver aminotransferase activity in about 5% of patients.

The only adverse effect that has occurred more often with rifapentine than with rifampin has been hyperuricemia when the drug was given twice-weekly; of note, hyperuricemia was attributed to pyrazinamide that was administered concomitantly. Other adverse reactions that occurred in 1-5% of patients included the following: hemoptysis, dizziness, hypertension, headache, gastrointestinal upset, rash, cytopenias, hematuria, pyuria, and proteinuria (Priftin package insert 2018).

Recently, Sanofi has reported the presence of nitrosamine impurities in rifapentine lots it has manufactured. These impurities have also been found in rifampin, another TB drug. Low amounts of nitrosamines are common in water and foods, including cured and grilled meats, dairy products and vegetables. Some types of nitrosamines have been shown to cause cancer in tested animals. Nitrosamine impurities may increase the risk of cancer if people are exposed to them above acceptable levels and over long periods of time (such as many years). The nitrosamine compound found in rifapentine is 1-cyclopentyl-4-nitrosopiperazine (CPNP). Although there are no data available to directly evaluate the carcinogenic potential of CPNP, information available on closely related nitrosamine compounds has been used to calculate lifetime exposure limits for and CPNP. The US Food and Drug Administration (FDA) has established an exposure limit of 0.1 ppm for

rifapentine; daily exposures above this level for prolonged periods of times (decades) could potentially increase the risk of some cancers. Short-term use is not considered to impose an increased risk of cancer.

Sanofi has tested all lots of rifapentine and identified levels of CPNP ranging from <1 ppm to up to 12 ppm. The company has proposed a temporary limit of 20 ppm of CPNP in rifapentine. To mitigate or avoid shortages and to help ensure patients have access to rifamycins, the FDA has agreed that manufacturers may temporarily distribute rifapentine containing CPNP levels <20 ppm until they can reduce or eliminate the impurities. The World Health Organization and the Global Drug Facility of the STOP TB Partnership have endorsed this recommendation. Sanofi will provide rifapentine for the trial, and each batch of medication will be tested prior to shipment. No batch with product containing ≥20 ppm will be used.

Isoniazid

Isoniazid is the hydrazide of isonicotinic acid and is one of the primary drugs for TB therapy. The activity of isoniazid is limited to the mycobacteria of the *M. tuberculosis* complex; it is bactericidal for rapidly dividing organisms and bacteriostatic for "resting" bacilli. The probable mechanism of action is the inhibition of the biosynthesis of mycolic acids, a component of the mycobacterial cell wall.

Isoniazid is generally well absorbed; food and antacids decrease the rate, but not the extent of absorption. Peak blood levels of isoniazid, 3 to 5 mcg/ml, are obtained 30 minutes to 2 hours after ingestion of routine doses (Peloquin 1999). It diffuses into all body fluids and cells and penetrates into the caseous material of a tuberculoma or pulmonary cavity. In the liver, it is acetylated to inactive metabolites, and 75% to 95% of the dose is excreted as inactive metabolites in the urine within 24 hours. Isoniazid clearance rates depend on 2 metabolic NAT2 gene-associated phenotypes, slow and fast acetylation, which are associated with race, but not sex (Ellard 1984). The isoniazid AUC among persons who have fast acetylation is 30% to 50% of that among persons who have slow acetylation.

Because isoniazid is well tolerated over a wide range of therapeutic doses, a single dose per body mass is recommended. Persons who have rapid acetylation achieve effective concentrations, while persons who have slow acetylation do not experience increased toxicity. Half-life ($t_{1/2}$) may vary from 1 hour in fast acetylators ($t_{1/2}$ < 90min) to 3 hours in slow acetylators ($t_{1/2}$ >90min). The usual adult dose of isoniazid is 5 mg/kg given once daily, up to a maximum of 300 mg given once daily.

Isoniazid decreases the clearance of some medications that are metabolized in the liver, particularly carbamazepine, phenytoin, and diazepam (Baciewicz 1985). However in the context of multidrug therapy including rifampin, these potential drug-drug interaction are of little significance because the effect of isoniazid is counteracted by the more potent opposing effect of rifampin (Kay 1985).

The total incidence of all adverse effects from isoniazid is approximately 5%, many of which do not require discontinuation of the drug. Peripheral neurotoxicity is dose dependent and it is uncommon (<0.2%) at conventional doses. The risk of peripheral neuritis increases for persons who are malnourished or predisposed to neuritis by other illnesses. Concomitant administration of pyridoxine (vitamin B_6) is recommended for these persons.

Other nervous system reactions are rare at normal doses, and they include convulsions, encephalopathy, optic neuritis, memory impairment, and psychosis. Gastrointestinal adverse effects include nausea, vomiting, and epigastric distress. Asymptomatic elevation of aminotransferases is common and occurs in 10-20% of persons receiving isoniazid. However, idiosyncratic severe hepatic reactions are uncommon but are more likely in older persons (up to 2.3% hepatitis incidence in persons more than 50 years old), and may be life threatening. Daily consumption of alcohol increases the risk of isoniazid-associated hepatotoxicity by approximately 4-fold.

WHO prequalified, ANVISA-approved isoniazid is available as 300 mg tablets and will be obtained from the Brazilian Ministry of Health.

Safety profile of study regimens

In June 1998, the US Food and Drug Administration (FDA) approved rifapentine (RPT) for the treatment of tuberculosis, the first new drug approved for tuberculosis in more than 25 years. In addition, the 3HP regimen is registered by the US FDA and is recommended for the prevention of TB disease by the US Centers for Disease Control and Prevention (CDC). The World Health Organization recommends 3HP for low burden, middle and high-income countries in the integrated guidelines for the programmatic management of LTBI. Tolerance of HP regimen was comparable to INH in the CPCRA/ACTG trial.

Isoniazid has been used in the treatment and prevention of tuberculosis for over 50 years, and its adverse event profile is well known. Rash, fever, jaundice, and peripheral neuritis are the most common INH-related adverse reactions. Concurrent administration of pyridoxine (vitamin B6) prevents INH-related peripheral neuropathy as well as nearly all other nervous system disorders attributable to INH administration. In this study, if a participant develops symptoms of peripheral neuropathy, pyridoxine may be administered at a dose of 25 mg with each dose of INH. The dose of pyridoxine may be increased to 50 mg with each dose of INH if symptoms of peripheral neuropathy persist.

RPT, like other rifamycins, causes red-orange discoloration of body fluids. In trials where RPT was combined with INH and other antituberculosis drugs, rates of adverse reactions were similar between rifampin and RPT, with increased liver aminotransferase activity in about 5% of patients (Priftin package insert 2018).

3HP. In TBTC 26, 3HP was also well-tolerated among study participants

compared to 9H. A synopsis of relevant adverse events and their frequencies is listed in Table 2.

Table 2. Drug-related adverse events in trials of RPT/INH for LTBI treatment

Study	Population	Treatment completion		Adverse Events		Comment
Schechter 2006	Household contacts in Brazil	3HP	93%	**3HP**	1%	DOPT, Trial terminated early
		2RZ	94%	2RZ	10%	
Martinson 2011	HIV+ not on ART, South Africa	**3HP**	96%	**3HP**	8.7/100 PY	DOPT, Continuous INH more toxic
		6H	84%	6H	15.4/100 PY	
Sterling 2011	Contacts, other high-risk, >2 yo, US, Canada, Brazil, Spain	**3HP**	82.1%	**3HP**	8.2%	DOPT, Toxicity related to study drugs
		9H	69.0%	9H	5.5%	
Belknap 2017	US, S. Africa, Spain	**3HP**-DOT	87.2%	**3HP**-DOT	7.1%	SAT=self-administered, SAT+ text messages
		**3HP**-SAT	74.0%	**3HP**-SAT	8.3%	
		**3HP**-SAT+	76.4%	**3HP**-SAT+	7.9%	
Swindells 2019	HIV+, >13 yo, multiple countries	**1HP**	97%	**1HP**	2.0/100 PY	P value for AE rates is 0.01
		9H	90%	9H	4.6/100 PY	
**Observational Studies**						
Chan 2019	Contacts >12 yo Taiwan	**3HP**	83.9%	**3HP**	12.0%	DOPT used for 3HP
		9H	78.8%	9H	9.4%	
Jo 2019	Health care workers, South Korea	**3HP**	85.0%	**3HP**	15%	DOPT used for 3HP (19% flu-like syndrome with 3HP)
		3HR	81.7%	3HR	10%	

Treatment completion rates and occurrence of adverse events in studies of 3HP and 1HP in various populations. Adverse events are reported differently based on published results. DOPT=directly observed preventive therapy; SAT=self-administered therapy; numeral=number of months; H=INH; P=rifapentine; R=rifampin; Z=pyrazinamide.

11.1.3 Study Product Supply and Accountability

Rifapentine will be donated by Sanofi. The company will provide rifapentine (RPT, Priftin®) in boxes containing 3 blister packs of 8 tablets each (24 tablets of 150 mg each).

Isoniazid will be obtained locally. In the Brazilian national TB program, isoniazid is available in 100 or 300 mg tablets. The study will use 300 mg tabs.

Rifapentine and isoniazid will be stored under dry and light-limited conditions at temperatures between 15-25° C.

Quality assured pyridoxine will be obtained from a supplier in Brazil, and shipped to site along with the isoniazid and rifapentine.

Study medication will be managed and controlled according to the Standard Operating Procedures for Distribution, Storage and Accountability. Study sites are responsible to maintain records of all study products received, dispensed, returned and destroyed (if unused or returned).

11.1.4 Assessment of Participant Adherence with Study Product(s)/Intervention(s)

Prior to treatment initiation, each participant will receive treatment adherence counselling which will include information regarding the treatment, side effects, assistance with methods for remembering to take the medication, and discussion of any anticipated problems with taking medication. At each study visit, participants will be reminded to take all their medication. HP will be taken as self-administered therapy (SAT) in both treatment arms.

In both arms, the study team will use several measures to monitor treatment completion, including:

- Participants will maintain a medication diary for SAT
- Self-reported dosing at each monitoring visit
- Pill counts. Participants will bring their unused study medication with them. Study staff will count the remaining pills to estimate the number of scheduled doses taken
- Pharmacologic monitoring:

  Isoniazid urine testing. The Isoscreen (GFC Diagnostics) point-of-care test, a colorimetric test based on the Arkansas method, (Guerra) will be used according to manufacturer-recommended methods that we have previously validated in Brazil. (Kendall) In the presence of isoniazid metabolites, this test causes urine to change color from yellow to green to indicate low levels detected or to blue-black to indicate high levels detected; urine remains yellow with no visible color change at five minutes if no isoniazid metabolites are detected. The sensitivity of any positive result has been estimated as 95-99% at 24 hours and 85% (95% binomial confidence interval 76-91%) at 48 hours after the last dose of 300mg of isoniazid. The sensitivity of a strongly positive result is estimated to be >99% at 12 hours, 68-82% at 24 hours, and 5% at 48 hours. Results are negative 72 hours after the last dose in 87% (78-92%) of patients. (Kendall)

  Tests will be performed by laboratory technicians at each site but not shared with study staff or patients, as the test is not FDA-approved and cannot be used for clinical management.

  Instructions for specimen collection and processing will be included in the study's Manual of Operations. (http://www.gfcdiagnostics.com/isoscreenprocedures.html)

  Rifapentine concentrations in dried blood spots (DBS). Rifapentine has a half-life of 14-16 hours and its des-acetyl metabolite has a Tmax of 24 hours. (Dooley, 2012) At the dosages proposed for this trial, detectable levels of both rifapentine (RPT) and des-acetyl rifapentine (des-RPT) can be detected in whole blood and plasma for up to 50 hours. Investigators at

the Johns Hopkins Clinical Pharmacology Analytic Laboratory (CPAL) have validated the accuracy and robustness of DBS measurement of RPT and des-RPT. (Parsons) They compared plasma and whole blood-DBS levels from healthy volunteers and found acceptable inter-subject variability. The mean concentration and lower limit of quantification for RPT were 2644 ng/ml and 53.5 ng/ml for DBS and 2475 ng/ml and 56.6 ng/ml for plasma. Results were reliable after 11 weeks of storage at room temperature without light.

We will collect whole blood by venipuncture in EDTA tubes and spot 25ul on Whatman protein saver 903 cards. After air-drying, cards will be stored at ambient temperature in plastic bags away from light. Batches of cards will be shipped to Johns Hopkins quarterly and stored at the CPAL. RPT and des-RPT analyses will be performed in batches. A 6 mm spot will be punched using a Perkin-Elmer DBS puncher and placed in a 96 deep-well plate. An extraction solvent of 90:10 methanol-to-50 mM ammonium formate buffer ratio with 0.5 mg/ml ascorbic acid will be added and the plates will be capped and mixed on a titer plate shaker for 1 h. Aliquots of 400 ul will be withdrawn and analyzed by LC-MS/MS, as previously described. (Parsons)

11.1.5 Concomitant Medications and Procedures

The site investigator will review concomitant medications at screening, the enrollment visit, and all clinic visits, tracking start and stop dates, and dose changes on the clinical evaluation source document. Clinical research team members will track the list on a dedicated concomitant medication CRF.

Whenever a concomitant medication or study product is initiated or the dose changed, investigators should review the concomitant medications' and study products' most recent package inserts to check for the most current information on drug interactions, contraindications, and precautions.

11.1.6 Permitted Medications and Procedures

All medications and procedures are permitted that comport with inclusion and exclusion criteria.

11.1.7 Prohibited Medications and Procedures

A list of prohibited medications will be included in the MOP; patients requiring prohibited medications will not be enrolled. If use of prohibited drugs is required during treatment, study drugs will be stopped and the participant will be considered non-adherent thereafter.

11.1.8 Precautionary Medications and Procedures

Rifapentine is a strong inducer of metabolizing enzymes, so there is a risk of drug-drug interactions.

All concomitant medications, dosages, frequencies, and routes of administration will be recorded and data-entered. Site investigators will consult the list of prohibited and precautionary medications (Appendix IV) prior to starting any new medication to minimize the potential for drug-drug interactions.

11.1.9 Required Medications

Rifapentine and isoniazid as previously described

11.1.10 Rescue Medications

The first dose of rifapentine/isoniazid will be administered in the research clinic by a licensed health care professional. The clinic will be equipped with standard emergency resuscitation equipment, IV fluids, medications, and oxygen.

12. STUDY PROCEDURES/EVALUATIONS

12.1 Clinical Evaluations and Procedures

12.1.1 Screening

Obtain written informed consent or parental permission and assent *before* performing any screening procedures.

1. Verify study entry eligibility criteria are met
2. Record locator information for contacting participant throughout study
3. Medical history
   The medical history must include all prior TB and liver disease. In addition, it must include all pre-existing and new diagnoses within 30 days prior to study entry. Allergies to any medications and their formulations must be documented. Medical history and complete physical examination, including concomitant medication history
4. Medication History
   A medication history including all prescription and nonprescription medications taken within 30 days prior to study entry must be present. Include actual or estimated start and stop dates.
5. Conduct a complete physical exam
   A complete physical examination at entry is to include at a minimum an examination of the skin, head, mouth, and neck; auscultation of the chest; cardiac exam; abdominal exam; examination of the lower extremities for edema.

6. The complete physical exam will also include signs and symptoms, diagnoses, vital signs (temperature, pulse, respiration rate, and blood pressure), height, and weight

7. TB symptom screen
Current cough, fever, night sweats, or unintentional weight loss, lymphadenopathy and/or other abnormalities on lung examination.

8. Chest X-ray (CXR) to exclude active TB disease

9. Persons with symptoms or signs of active TB or an abnormal chest X-ray are ineligible to participate in the study, and should be referred for investigation by health clinic staff according to local guidelines. Do not proceed with enrollment.

10. Collect blood for chemistry panel including liver function tests (LFT), complete blood count (CBC), hepatitis B surface antigen (HBsAg), and interferon gamma release assay (IGRA) or TST if a documented result is not available within 90 days of screening.

11. HIV rapid test to confirm HIV negative status.

12. For individuals of childbearing potential, collect urine for βHCG.

Provide counseling and document the need for contraception and the risk of rifapentine reducing the effectiveness of oral and injectable hormonal contraception. A second barrier method should be used during the 24-week study of the patient's choice with medical advice. For those women who for some reason cannot get pregnant or who do not have sex, the use of contraceptives is not mandatory.

Screening evaluations must occur prior to the participant's starting study medications. Screening evaluations to determine eligibility must be completed within 7 days prior to study enrolment.

12.1.2 HIV Counseling and Testing

Counseling prior to and following HIV testing, and reporting HIV testing results will follow local guidelines and regulations at each site.

For study purposes, HIV-1 infection is defined as a positive result using any licensed rapid HIV test or any licensed HIV enzyme or chemiluminescence immunoassay (E/CIA) test kit. Negative study HIV testing does not require confirmatory testing. Note: the term 'licensed' refers to an FDA-approved kit or, for study sites located in countries other than the United States, a kit that has been certified or licensed by an oversight body within that country.

12.1.3 Enrollment (within 7 days of screening)

1. Verify that participant continues to meet study entry eligibility criteria. Update locator information for contacting participant throughout study, and collect concomitant medication history.

2. Repeat TB symptom screen (current cough, fever, night sweats, or unintentional weight loss). Persons with symptoms or signs suggestive of TB are ineligible to participate in the study, and should be referred for investigation by health clinic staff according to local guidelines.

3. For participants of childbearing potential, collect urine for βHCG, only if a result is not available from within 48 hours of enrolment.

4. Randomize to a treatment arm according to the study's randomization procedure.

Once randomized and enrolled, study staff will teach participants when and how to take their study regimen; including what to do if a dose is late, missed, or vomited. Participants will take their HP at home with a high fat diet to promote absorption.

Study staff will teach examples of common, local, culturally appropriate high fat foods that are available within the participant's socioeconomic status. Study staff will administer each participant's first dose with a standardized high fat meal in clinic as an example.

Participant will record her or his first dose in the medication diary with staff guidance.

5. Patient costing survey

To estimate any additional patient-level and societal costs of 1HP and 3HP (*e.g.,* ancillary healthcare visits, transportation to and from the clinic, use of formal social support programs, and costs associated with adverse events), we will randomly select 50 consenting patients in each arm of the trial to administer a patient cost survey. This patient cost survey will ask participants to record total duration of clinic visits, travel time to the clinic, and all out-of-pocket and indirect costs associated with their preventive TB treatment care/study participation through completion of treatment. Additional questions will be asked to capture information on the amount of time participants spend at home completing intervention-related activities (e.g. taking pills, completing adherence logs/diaries) and any costs associated with managing TPT-associated adverse events. The patient costing questionnaire will be integrated into existing study surveys and instruments as described in the Manual of Operations (MOP).

12.1.4 Follow-up visits

At all Visits for both treatment arms:

Targeted Physical Exam

A targeted physical examination at post-entry visits is to include vital signs (temperature, pulse, respiration rate, and blood pressure) and weight. It should be driven by any previously identified or new signs or symptoms including diagnoses that the participant has experienced since the last visit.

Signs and Symptoms

Record all signs and symptoms and grate according to the DAIDS grading criteria.

Diagnoses

Record all new diagnoses or worsening of a pre-existing condition, and grade according to the DAIDS grading criteria.

Concomitant Medications

All new concomitant medications taken since the last report and change in current concomitant medications since the last report should be recorded, including start and stop dates.

Study Treatment Modifications

Record all study drug modifications, including initial doses, participant-initiated and/or protocol-mandated modifications, inadvertent and deliberate interruptions (i.e., 3 or more missed doses) at each visit.
Record permanent discontinuation of treatment.

Patient Costing Survey

The patient costing questionnaires will be administered to 50 patients who were selected at the time of enrolment to complete this survey at two time points, namely the completion of treatment and the end of the follow-up period.

Arm A: Rifapentine 600 mg daily and isoniazid 300 mg daily for 4 weeks (1HP)

Arm A will have 250 participants.

Weeks 0-4

All daily doses of rifapentine/isoniazid (HP) will be self-administered at home by the participant, except for the initial dose which will be supervised by the study staff in clinic. Participants will record their HP dose taking and dose-related food intake/time on a medication diary card (Appendix II).

The research pharmacy at each site will dispense 15 or 16 rifapentine blister packs, each with 8 tablets (2 doses per pack, 30-32 doses total) and 30 or 32 300 mg INH tablets to cover the visit window period. An extra 2-4 days of medication will be provided to allow for vomited doses and to reduce the likelihood of medication dumping. Participants will be told to bring unused medication back to clinic for the 2 and 4 week visits. At enrolment, 50 participants will be selected to complete a patient costing survey and be asked to complete this survey two additional times during their follow visits (at completion of treatment and at the end of the study participation).

Week 2 (+/- 3 days)
Day 14

The participant will arrive to clinic at least an hour early, so that everything is in place for drug sampling events to begin on time. Check with the participant about yesterday's HP dosing and to be sure that s/he *did not take today's HP* dose. Check to be sure s/he brought today's HP dose to clinic.

Record the exact time the sample blood and urine was collected.
At any time during the events of Day 14 (Week 2 visit) (but before today's dose of HP), conduct the following assessments:

- TB symptom screen
- targeted physical exam
- adverse event assessment
- adherence questionnaire
- pill count of HP, review of medication diary card
- CBC
- chemistry panel including liver function tests
- Dried blood spot and urine collection for drug sampling
- Sampling for NAT2 gene
- urine pregnancy test for participants of childbearing potential

Week4 (+/- 3 days
Day 28

The participant will arrive to clinic at least an hour early, so that everything is in place for drug sampling events to begin on time. Check with the participant about yesterday's HP dosing and to be sure that s/he *did not take today's HP* dose. Check to be sure s/he brought today's HP dose to clinic.

Record the exact time the sample blood and urine was collected.
At any time during the events of Day 28 (Week 4 visit) (but before today's dose of HP), conduct the following assessments:
- TB symptom screen
- targeted physical exam

- adverse event assessment
- adherence questionnaire
- patient costing survey
- pill count of returned HP, review of medication diary card
- CBC
- chemistry panel including liver function tests
- Dried blood spot and urine collection for drug sampling
- urine pregnancy test for participants of childbearing potential

If participant is assessed to be stable on HP, s/he may take today's dose.

This is the final dose for the 1HP regimen.

Week 8 (+/- 3 days)

Study team will conduct the following assessments:
- TB symptom screen
- targeted physical exam
- adverse event assessment
- urine pregnancy test for participants of childbearing potential

Week 12 (+/- 3 days)

The study team will conduct the following assessments:
- TB symptom screen
- targeted physical exam
- adverse event assessment
- urine pregnancy test for participants of childbearing potential

Week 16 (+/- 3 days)

The study team will conduct the following assessments:
- TB symptom screen
- targeted physical exam
- adverse event assessment
- urine pregnancy test for participants of childbearing potential

Week 24 (+/- 3 days)

The study team will conduct the following assessments:
-TB symptom screen
- targeted physical exam
- adverse event assessment
- urine pregnancy test for participants of childbearing potential
- patient costing survey

This is the final study visit

Arm B: Rifapentine 900 mg once-weekly and isoniazid 900 mg once-weekly for 12 weeks (3HP)

Arm B will have 250 participants.

Weeks 0-4
All once-weekly doses of rifapentine/isoniazid (HP) will be self-administered at home by the participant, except for the initial dose which will be supervised by the study staff in clinic. Participants will record their HP dose taking and dose-related food intake/time on a medication diary card (Appendix II).

The research pharmacy at each site will dispense 4 rifapentine blister packs, each with 8 tablets (1.33 doses per pack, 5.33 doses total) and 12 or 15 300 mg INH tablets to cover the visit window period. An extra dose of medication will be provide to allow for vomited doses and to reduce the likelihood of medication dumping. Participants will be told to bring unused medication back to clinic for the 2, 4, 8 and 12 week visits.
At enrolment, 50 participants will be selected to complete the patient costing survey and be asked to complete this same survey two additional times during their follow visits (at completion of treatment and at the end of the study participation)
Week 2 (+/- 3 days)

The study team will conduct the following assessments:
- TB symptom screen
- targeted physical exam
- adverse event assessment
- adherence questionnaire
- Pill count of HP, review of medication diary card
- CBC
- chemistry panel including liver function tests
- Urine pregnancy test for participants of childbearing potential

Week 3 (+/- 3 days for visit)
Day 22 (24 hours after HP dose #4 was taken)

The participant will arrive to clinic at least an hour early, so that everything is in place for drug sampling events to begin on time.

Collect the blood and urine for drug sampling and record the exact time the sample blood and urine was collected.

The study team will conduct the following assessments:
- TB symptom screen
- targeted physical exam
- adverse event assessment
- adherence questionnaire

- pill count of returned HP, review of medication diary card
- CBC
- chemistry panel including liver function tests
- Dried blood spot and urine collection for drug sampling
- Sampling for NAT2 gene
- urine pregnancy test for participants of childbearing potential

Week 8 (+/- 3 days)
Study team will conduct the following assessments:
- TB symptom screen
- targeted physical exam
- adverse event assessment
- adherence questionnaire
- pill count of returned HP, review of medication diary card
- CBC
- chemistry panel including liver function tests
- urine pregnancy test for participants of childbearing potential

Week 12 (+/- 3 days for visit, but not for drug sampling)
Day 85 (24 hours after HP dose #12 was taken)
The participant will arrive to clinic at least an hour early, so that everything is in place for drug sampling events to begin on time.

Collect the blood and urine for drug sampling and record the exact time the sample blood and urine was collected.

The study team will conduct the following assessments:
- TB symptom screen
- targeted physical exam
- adverse event assessment
- adherence questionnaire
- patient costing survey
- pill count of returned HP, review of medication diary card
- CBC
- chemistry panel including liver function tests
- Dried blood spot and urine collection for drug sampling
- urine pregnancy test for participants of childbearing potential

This is the final dose for the 3HP regimen.

Week 16 (+/- 3 days)

The study team will conduct the following assessments:
- TB symptom screen
- targeted physical exam
- adverse event assessment

- urine pregnancy test for participants of childbearing potential

Week 24 (+/- 3 days)

The study team will conduct the following assessments:
- TB symptom screen
- targeted physical exam
- adverse event assessment
- urine pregnancy test for participants of childbearing potential
- patient costing survey

This is the final study visit.

12.1.5 Treatment completion and successful treatment.

1HP
Completion of therapy is defined as having completed 28 daily doses of treatment within 6 weeks. Treatment success is defined as having taken at least 25 doses of medication confirmed by patient report and pill count, and having rifapentine or isoniazid detected in urine or DBS specimens.

3HP
Completion of therapy is defined as having completed at least 12 weekly doses of treatment within 18 weeks. Treatment success is defined as having taken at least 11 doses of medication confirmed by patient report and pill count, and having rifapentine or isoniazid detected in urine and DBS specimens.

Figure 2 shows the algorithm that will be used to classify patients as having treatment success of failure. This hierarchical approach begins with self-reported adherence, then uses pill count, and finally a composite of test results for isoniazid in urine and rifapentine and des-RPT in DBS.

Figure 2

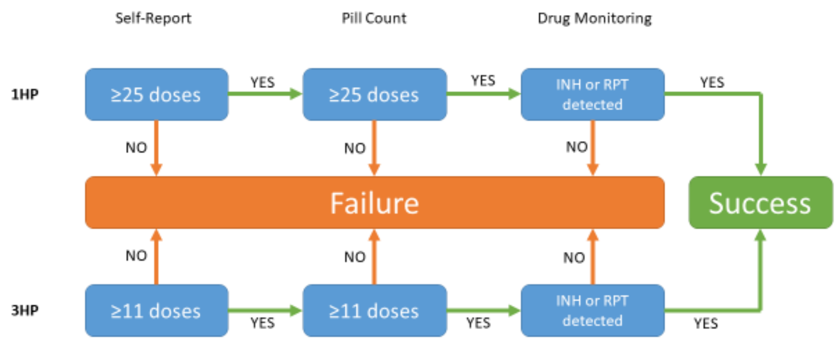

12.2 Laboratory Evaluations

12.2.1 Specimen Preparation, Handling and Shipping

Transmission of HIV and other blood borne pathogens can occur through contact with contaminated needles, blood, and blood products. Respiratory pathogens such as Mycobacterium tuberculosis (MTB) are transmitted by inhalation of droplet nuclei.

Appropriate blood, secretion, and respiratory precautions will be employed by all personnel in the collection of clinical samples and the shipping and handling of all clinical samples and isolates for this study, as currently recommended by the Agência Nacional de Vigilância Sanitaria (ANVISA), the WHO internationally, and the National Institutes of Health. Human biological samples sent to the United States of America will be immediately destroyed after performing the test according to the flow of the health unit.

All protocol specimens will be shipped using packaging that meets requirements specified by the International Air Transport Association (IATA) Dangerous Goods Regulations for UN 3373, Biological Substance, Category B, and Packing Instruction 650. Culture isolates, if obtained in this study, are to be shipped as specified for UN 2814 Category A Infectious Substances.

12.2.2 Total Blood Volumes per study group

Arm A: Rifapentine 600 mg daily and isoniazid 300 mg daily for 4 weeks

Laboratory Evaluations	Screening	Enroll Ment	Week 2	Week 4	Week 8	Week 12	Week 16	Week 24
Hematology	**2**ml EDTA tube		**2** ml EDTA tube	**2** ml EDTA tube				
Chemistry, incl LFTs	**2.5**ml SST tube		**2.5** ml SST tube	**2.5** ml SST tube				
Hepatitis B Surface Antigen (HBsAg)	**3.5**ml SST tube							
HIV-1 antibody test	Rapid test							
TB Diagnostics								
IGRA	**6**ml lithium heparin tube							
Pharmacology (DBS)			0.5ml EDTA tube	0.5ml EDTA tube				

RPT monitoring			DBS	DBS				
NAT2 gene			If DBS					
**TOTAL**	**14 ml**	**0**	**5 ml**	**5 ml**	**0**	**0**	**0**	**0**
**24 ml**								

<u>Arm B</u>: Rifapentine 900 mg weekly and isoniazid 900 mg weekly for 12 weeks

Laboratory Evaluations	Screening	Enrol l Ment	Week 2	Week 4	Week 8	Week 12	Week 16	Week 24
Hematology	**2**ml EDTA tube		**2**ml EDTA tube	**2**ml EDTA tube	**2**ml EDTA tube	**2**ml EDTA tube		
Chemistry, incl LFTs	**2.5**ml SST tube		**2.5**ml SST tube	**2.5**ml SST tube	**2.5**ml SST tube	**2.5**ml SST tube		
Hepatitis B Surface Antigen (HBsAg)	**3.5**ml SST tube							
HIV-1 antibody test	Rapid test							
TB Diagnostics								
IGRA	**6**ml lithium heparin tube							
Pharmacology (DBS)				0.5 EDTA tube		0.5 EDTA tube		
RPT monitoring				DBS		DBS		
NAT2 gene				If DBS				
**TOTAL**	**14 ml**	**0**	**4.5 ml**	**5 ml**	**4.5 ml**	**5 ml**	**0**	**0**
**33 ml**								

12.3 Other Study Procedures and Evaluations

12.3.1 Early Termination Visit

Participants who meet any one or more of the following criteria will be discontinued from study treatment:

- Pregnancy

- Any clinical adverse event, laboratory abnormality, intercurrent illness, other medical condition or situation occurs such that continued administration of study treatment is not in the best interest of the participant

- Participant request for premature discontinuation of study treatment

A participant who is discontinued from study treatment should still remain on study, and continue with follow up visits as described in the schedule of evaluations.

A participant who withdraws consent will not continue to be followed.

12.3.2 Managing a participant who becomes pregnant during the study

A participant who becomes pregnant while receiving study therapy will be discontinued from study treatment and treated according to National Tuberculosis Program or local guidelines. The participant will continue with scheduled study follow-up, classified as being on a non-study regimen, and will not receive study radiographs. Pregnancy outcome will be recorded on study forms.

A participant who becomes pregnant while in study follow-up (not on study treatment) will continue with scheduled study follow-up and will not receive study radiographs. Pregnancy outcome will be recorded on study forms.

12.3.3 Managing participants who are discontinued from study treatment in the setting of an adverse event or the investigator judges that discontinuation of study treatment is in the participant's best interest

The site investigator may discontinue a participant from treatment in the event of a severe or serious adverse event, or at any time if the investigator thinks discontinuation is in the participant's best interest.

For study purposes, such participants should continue to be followed in the study for outcome determination in accordance with the study MOOP, unless the participant withdraws consent. The participant should be referred to appropriate local sources of care for management of medical problems that cannot reasonably be managed by the study team.

12.3.4 Managing a participant who develops clinical signs of TB

Clinical suspicion of TB is defined as:

Objective evidence of TB (cough, fever, night sweats, weight loss, or

hemoptysis) based on history and/or physical examination **PLUS** radiographic findings, and without concurrent illness that would explain the findings.

Sputum Acid Fast Bacilli Smear, Gene Xpert, and Sputum Culture X 2
Whenever active TB is suspected, Gene Xpert, sputum AFB smear and sputum culture X 2 should be performed. Positive cultures should undergo speciation, and drug susceptibility testing if positive for MTB. A regional or central reference laboratory may be used.

12.3.5 Managing a participant who requests premature discontinuation from study treatment

A participant may request premature discontinuation from study treatment. A post early termination visit should be performed in 14 days to assess for any late-onset adverse events.

The participant should continue to be followed in the study per the study schedule, unless the participant withdraws consent. The participant should be referred to appropriate local sources of care for management of tuberculosis.

12.3.6 Managing a participant who is incarcerated after enrollment

This study will not enroll prisoners. However, it is possible that a participant could become incarcerated after enrollment. If an enrolled individual is incarcerated, then study medications will be stopped and the participant will be treated with TPT according to the standards of the institution in which s/he is incarcerated.

While incarcerated, individuals will not be followed in the study. When the individual is no longer incarcerated, study treatment and/or study follow-up may continue at the discretion of the investigator, if timing is within the window of all other protocol criteria.

12.3.7 Post Early Termination Visit (Visit after Early Termination) for Participants Terminating before Completion of their Assigned Study Treatment

The early termination visit should occur 14 days (+/- 3 days) after stopping study drugs, in order to assess for late manifesting adverse events.

The following should be performed:

- Review contact information
- Symptom assessment
- Adverse event assessment

- Concomitant medication assessment

Participants experiencing an adverse event at the time of early termination should be followed until resolution or stabilization of the event.

Participants who were receiving study treatment at the time of early termination will be referred to local sources for tuberculosis care.

For study purposes, such participants should continue to be followed in the study for outcome determination in accordance with the study MOOP, unless the participant withdraws consent.

A participant who withdraws consent will not have follow-up study procedures.

12.3.8 Missed Visit

A visit is classified as missed if it does not occur within the defined visit window of +/- 3 days from the scheduled visit.Register as unscheduled visit, detailed below.

12.3.9 Unscheduled Visit

An unscheduled visit refers to a protocol-required study evaluation that does not occur within the defined visit window.

During an unscheduled visit, all procedures described in the protocol for the particular missed visit should be completed, including a study visit that occur solely because of an adverse event or a possible poor treatment response.

12.3.10 Final Study Visit

The final study visit is at week 24, for TB symptom screen, AE assessment, and targeted physical exam.

12.3.11 Time-and-Motion (TAM) Studies

To assess clinic staff and patient time and resources required for visits specific to 1HP and 3HP initiation and follow-up, research staff will conduct time-and-motion (TAM) studies. TAM studies are designed to capture the amount of time needed to provide each unit of clinical and/or laboratory services to the patients. Sampling for TAM data collection will be stratified to ensure a broad coverage of visits by time of day, day of week, and time of year, plus inclusion of participants who require additional clinical evaluation (*e.g.,* for side effects) so that the data represents a range of clinical operational conditions in our study setting. The TAM data collection requires interaction with clinic staff; however, only minimal clinic staff involvement (shadowing/observation of staff) is needed, and these studies will not involve collection of protected

health information (PHI). The TAM data collection will include a mix of directly observed (by study research staff) and self-reported data. All participating staff will provide informed consent. The TAM data will capture information on the amount of time spent conducting different TB procedures (*e.g.* assessing patient adherence) face-to-face with the patient, and other TB clinical tasks that are conducted without the presence of patients (e.g. administrative tasks such as review of medical charts).

13. SAFETY ASSESSMENT

13.1 Safety Assessment Overview

Responsibilities for ensuring the safety of study participants

National regulatory authorities, the sponsor, participating research institutions, Site Principal Investigator and clinical teams, and the study Principal Investigator share responsibility for ensuring that participants in this trial are exposed to the least possible risk of adverse events that may result from participation in this protocol.

The site principal investigator has a personal responsibility to closely monitor study participants and an inherent authority to take whatever measures necessary to ensure their safety. The principal investigator has the authority to terminate, suspend or require changes to the study for safety concerns. Site principal investigators determine severity and causality for each adverse event.

The protocol is approved by all local, national, and Sponsor-affiliated ethics committees. A referral network has been established for referring persons with adverse events to the hospital or local clinic.

13.2 Adverse Event Procedures and Reporting Requirements

13.2.1 Adverse Events

An adverse event is any untoward medical occurrence in a patient or clinical investigation subject administered a study agent and which does not necessarily have a causal relationship with the treatment received.

All Grade 2 or higher clinical or laboratory adverse events will be captured.

13.2.2 Study defined AEs

The study will capture study-defined adverse events including hypersensitivity syndrome, rash, peripheral neuropathy, hepatotoxicity, nausea and vomiting, and drug-related fever.

Participants will be trained to call the study team if they develop symptoms of hepatitis or rifamycin hypersensitivity syndrome, and the participant will have an unscheduled visit to evaluate as appropriate.

13.3.3 Serious Adverse Events

A serious adverse event (SAE) is defined as any untoward medical occurrence that

- results in death,
- is life-threatening,
- requires inpatient hospitalization or prolongation of existing hospitalization,
- results in persistent or significant disability/incapacity, or
- is a congenital anomaly/birth defect
- Other medically important conditions. This includes important medical events that may not be immediately life- threatening or result in death or hospitalization but may jeopardize the patient or may require intervention to prevent one of the outcomes listed in the definition above.

13.3.4 Adverse event (AE) reporting

Adverse events will be reported according to local and national regulatory requirements.

The purpose of AE reporting in this study is to ensure the safety of participants, and not to identify new AEs related to isoniazid or rifapentine. The 3HP regimen is approved by the US Food and Drug Administration (US FDA) and is recommended by WHO as TB preventive therapy. In this study, the use of 1HP in HIV-uninfected individuals is considered experimental. Investigators will monitor for adverse events in the study.

13.3.5 AEs requiring expedited reporting

The study will report all SAEs, including death from any cause, for any participant who had at least one dose of HP, as required by local and national regulatory agencies.

Any Grade 3 or higher hepatitis or hypersensitivity reaction; flu-like reaction,

or seizure will also be reported to the Principal Investigator, and Sponsor, and Johns Hopkins PI and study coordinator within 72 hours (three days) of site's knowledge.

Reporting a pregnancy will follow similar expedited reporting within 72 hours (three days of site's knowledge) to the Principal Investigator, and Sponsor, and Johns Hopkins PI and study coordinator.

13.3.6 AE Reporting period

The reporting period for AEs is from time of participant enrollment to the end of trial follow up for that participant. AEs occurring prior to exposure to the study agents in this trial do not require reporting.

13.3.7 Recording updated information on SAEs

Sites must follow each SAE until the SAE is resolved or stable if expected to remain chronic. Follow up should be according to clinical need, but not more than 45 days from the last visit.

Extra, annotated information and/or copies of test results may be provided separately.

13.3.8 Recurrent SAEs

If an SAE fully resolves but then re-occurs, the SAE is considered to be a new event.

13.3.9 Timeframe for SAE reporting

Clinical research sites must report all events that meet the regulatory agency definition of a SAE within 24 hours of awareness of the event. The event will be entered into the clinical database, and in addition, recorded on a SAE form and sent to the Johns Hopkins PI and central coordinator, and DMID via CROMS PVG.

SAEs will be submitted to all applicable ethical and regulatory agencies according to local and national requirements, or within 72 hours of the site becoming aware of the event.

13.3.10 Site investigator assessment and signature

A site physician investigator or sub-investigator must review and verify the completed SAE Form for accuracy and completeness and then sign the report. In the rare event that such physician(s) are not available for signature, sites may submit the SAE requiring reporting without the signature to meet reporting timeframe requirements. However, the completed SAE Form with

signature and any necessary corrections or additions must be submitted within 72 hours.

The site Principal Investigator (or designee) is responsible for designating at least one other physician at the site who can complete the form and provide signature to provide uninterrupted coverage of monitoring of SAEs.

Procedures

1. The site PI or designee evaluates the AE and determine whether it fulfils the criteria for seriousness.
2. If the SAE criteria is met, the SAE Form should be completed and signed by the responsible investigator.
3. The completed and signed SAE form should be submitted to regulatory authorities, and emailed to the Johns Hopkins PI and study coordinator.

14. CLINICAL MANAGEMENT

14.1 Clinical Management of Adverse Events

14.1.1 Toxicity grading tables

This study will grade toxicity and adverse events according to the *Division of AIDS (DAIDS) Table for Grading Adult and Pediatric Adverse Events, Corrected Version 2.1* (dated July 2017).

https://rsc.niaid.nih.gov/sites/default/files/daidsgradingcorrectedv21.pdf
See Appendix V.

14.2 Toxicity management

14.2.1 Gastrointestinal

For nausea/vomiting and/or diarrhea ≥Grade 3 or for Grade 2 toxicity if symptoms were not present at the previous visit, all study drugs should be withheld until the symptoms have resolved. Reintroduce study drugs with caution. Antiemetic and antidiarrheal medication may be used at the site investigator's discretion. Labs should be checked as clinically indicated.

Every effort should be made to reintroduce the study drugs after symptoms improve or resolve.

14.2.2 Cutaneous

For Grade 2 or Grade 3 cutaneous events that occur after the first dose of HP, all study drugs should be withheld until the toxicity resolves. Study drugs should be reintroduced with caution.

Grade 4 cutaneous or mucocutaneous rash is a serious toxicity, and all study drugs should be permanently discontinued.

Study drugs may be discontinued at the discretion of the site investigator. Similarly, study drugs that are temporarily interrupted may be reintroduced at the discretion of the site investigator.

14.2.3 Rifamycin hypersensitivity syndrome (RHS)

Signs and symptoms of RHS may include hypotension, urticaria, angioedema, acute bronchospasm, conjunctivitis, thrombocytopenia, neutropenia or flu-like syndrome (weakness, fatigue, muscle pain, nausea, vomiting, headache, fever, chills, aches, rash, itching, sweats, dizziness, shortness of breath, chest pain, cough, syncope, palpitations). There have been reports of anaphylaxis

This syndrome has been associated with intermittent rifampin administration, and was reported in a rifapentine and isoniazid regimen study (Langdon 2004). In Study 26, flu-like and systemic drug reactions were reported by 3.5% of 3HP recipients compared to 0.4% of 9h recipients. More recently, among healthy volunteers receiving HP and dolutegravir, 2 of 4 volunteers experienced rifamycins hypersensitivity syndrome fever, hypotension, elevated transaminases), halting the study. (Brooks 2018) No hypersensitivity reactions were found with daily HP in the BRIEF TB study. We will carefully monitor participants for signs and symptoms of hypersensitivity reactions.

<u>For participants who develop signs or symptoms of RHS</u>:

- Hold study drug regimen
- Assess for RHS through clinical evaluation and laboratory testing, including comprehensive metabolic panel, CBC with differential, and other tests that are necessary to exclude likely alternative diagnoses. (e.g., should symptoms suggest influenza, a nasopharyngeal aspirate for viral testing could be sent).
- If Grade 3 or higher AE meets the definition for RHS (and symptoms are not clearly attributable to an alternative diagnosis), permanently discontinue the study drug regimen and notify the Core Team.

See Appendix III for a rifamycin hypersensitivity syndrome worksheet.

14.2.4 Drug-Associated Fever

If ≥Grade 3, all study drugs should be held until the participant is afebrile. Study drugs should be reintroduced with caution. If fever reoccurs on reintroduction, permanently discontinue study drugs.

14.2.5. Liver Toxicity

All study drugs will be stopped permanently if any of the following liver chemistry criteria are met:

- ALT ≥3 × ULN **and** bilirubin ≥2 × ULN (>35% direct bilirubin; bilirubin fractionation required)
    - NOTE: Serum bilirubin fractionation should be performed if testing is available. If testing is unavailable, record presence of detectable urinary bilirubin on dipstick, indicating direct bilirubin elevations and suggesting liver injury. If testing is unavailable and a subject meets the criterion of total bilirubin ≥2 × ULN, then the event meets liver stopping criteria;
- ALT ≥3 × ULN with symptoms or worsening of acute hepatitis or hypersensitivity such as fatigue, nausea, vomiting, right upper quadrant pain or tenderness, fever, rash, or eosinophilia, **or**
- ALT ≥5 × ULN; regardless of symptoms

In the event of liver toxicity, appropriate evaluations for alternative causes may be pursued (viral hepatitis, alcohol exposure, etc.).

14.2.6 Peripheral Neuropathy

Peripheral neuropathy associated with INH is less common in HIV-uninfected individuals. Individuals who are at risk for developing peripheral neuropathy may be started on pyridoxine (vitamin B6) at the Investigator's discretion.

Participants with baseline peripheral neuropathy <Grade 2 according to the DAIDS Grading Table may be entered into the study, but should be monitored carefully for progression of the neuropathy.

For Grade 1 or 2, continue the study drugs and follow the participant more frequently for progression of peripheral neuropathy. Consider starting or increasing vitamin B6 dose.

For Grade 3 or 4, discontinue all study medication until toxicity resolves to Grade ≤2. If peripheral neuropathy does not resolve despite discontinuation of study drugs, study drugs may be reintroduced at the site investigator's discretion.

14.2.7 Other Toxicities

For toxicities that do not fall into one of the scenarios above, management will be as follows:

Grade 1 and 2: Participants may continue HP at the discretion of the site investigator with careful follow-up.

Grade 3 and 4: Study drugs should be held until symptoms have resolved (or until ≤Grade 2 or within normal limits). Study drugs may be permanently discontinued at the discretion of site investigator.

Site investigators are strongly encouraged to discuss discontinuations and reintroductions of study treatment with the core investigators to ensure consistency across sites.

If study drugs are suspended for any reason, participants in Arm A will have 8 weeks from enrollment to complete 28 doses of HP. Participants in Arm B will have 16 weeks from enrollment to complete at least 11 doses of HP. If the break in treatment is longer than 4 weeks, the participant will need to be rescreened for TB before recommencing HP.

14.3 Pregnancy

Pregnancy is an exclusion criteria for enrollment. An individual who becomes pregnant during the study drug treatment phase will be managed according to the plan described in Section 12.

14.4 Criteria for Discontinuation of Study Medications

14.4.1 Criteria for Permanent Discontinuation

- Discontinuation for an Individual Participant
- Drug-related toxicity requiring tx discontinuation
- Pregnancy or breastfeeding. HP will be discontinued and the participant will be referred to routine care.
- Diagnosed with active TB disease
- Completion of study events
- Request by participant to terminate treatment
- Clinical reasons believed life threatening by the physician, even if not addressed in the toxicity section of the protocol
- Acquires HIV infection while on study

14.4.2 Premature study discontinuation

- Request by the participant to withdraw
- Pregnancy or breastfeeding
- Request of the primary care provider if s/he thinks the study is no longer in the best interest of the participant
- Participant judged by the investigator to be at significant risk of failing to comply with the provisions of the protocol as to cause harm to self or seriously interfere with the validity of the study results

All participants who discontinue therapy will be followed up according to their assigned schedule until the end of the study.

15. STATISTICAL CONSIDERATIONS

15.1 Overview and General Design Issues

While the 3HP regimen is proven safe and effective in HIV-positive and –negative people, 1HP has only been shown to be safe and efficacious in HIV-positive people. An efficacy study in HIV-uninfected individuals would be useful for purposes of replication of the single study demonstrating the non-inferiority of 1HP, but such a trial would be extremely costly and time -consuming. The ACTG BRIEF TB trial took 6 years at a cost of >$25 million with its sample size of 3000.

It is likely that a non-inferiority efficacy trial in HIV negatives would require a sample size of at least 4500. In the history of TB preventive therapy, a regimen that is efficacious in one population has always been efficacious in others. For 3HP, efficacy was found to be quite similar in HIV+ people and HIV-negative, as well as in children. What can differ, however, is toxicity and tolerability. As shown with the 2-month rifampin and pyrazinamide (2RZ) regimen, safety in people with HIV does not ensure safety in HIV-negative people. Thus, a safety and adherence study is both necessary and preferred.

1HP is now recommended by the WHO as an alternate first-line regimen for treatment of latent TB infection. The USPHS OI Task Force and the CDC have indicated that they will likely add language recommending 1HP for HIV seropositive adults and adolescents in the near future.

15.2 Study Outcomes

15.2.1 Primary Outcomes

(1) Successful completion of TBT with ≥90% adherence documented by self-report, pill count, and pharmacologic monitoring.

Definitions:
For 1HP, successful completion of TPT is defined as having taken at least 25 doses of self-administered daily medication within 8 weeks of enrollment confirmed by patient report and pill count, and having rifapentine or isoniazid detected in urine and DBS specimens.

For 3HP, successful completion of TPT is defined as having taken at least 11 doses of self-administered weekly medication within 16 weeks as confirmed by patient report and pill count and having rifapentine or isoniazid detected in urine and DBS specimens.

(2) Safety of treatment regimens, defined as occurrence of Grade 2 or higher targeted safety events and discontinuation of study medications because of side effects.

Targeted safety events are hypersensitivity syndrome, rash, peripheral neuropathy, hepatotoxicity, nausea and vomiting, and drug-related fever.

15.2.2 Secondary Outcome

(1) To compare the cost-effectiveness of 1HP and 3HP using a societal approach, modeling the incremental cost-effectiveness of 1HP vs 3HP, 6H, and no treatment. This will operate under a separate sub-study protocol.
(2) Analyze, in each of the health units in the study, the time needed to provide comprehensive care to patients.

15.3 Study Hypotheses

(1) Successful completion of TBT with ≥90% adherence documented by self-report, pill count, and pharmacologic monitoring.

Hypothesis: Successful treatment with 1HP will be superior to 3HP, with 90% of individuals randomized to 1HP taking >90% of prescribed doses vs 80% of those on 3HP.

(2) Safety of treatment regimens, defined as occurrence of Grade 2 or higher targeted safety events and discontinuation of study medications because of side effects. Targeted safety events are hypersensitivity syndrome, rash, peripheral neuropathy, hepatotoxicity, nausea and vomiting, and drug-related fever.

Hypothesis: We hypothesize that the safety of 1HP will be superior to 3HP, with a rate of targeted adverse events or medication discontinuation of 6% vs. 13% for 3HP.

15.4 Sample Size Considerations

The sample size target is 500 evaluable adolescents and adults. Having each participant complete all of the scheduled drug sampling evaluations will provide approximately 1000 rifapentine and isoniazid pharmacological data points.

Assumptions, Outcome 1:

Successful treatment with 3HP = 80%
In clinical trials, reported completion rates of 3HP have ranged from 84-92% with directly observed therapy. In the CDC-sponsored iAdhere Trial, completion of self-administered 3HP was only 74-76%. In this study, which will use self-administered treatment, we estimate a completion rate of 80%. (Belknap 2017)

Successful treatment with 1HP = 90%
Completion of self-administered 1HP was 97% in the BRIEF TB Study, which was undertaken at research clinics in the ACTG and IMPAACT networks. We estimate that in a more real-world setting adherence will be slightly lower, but should still be at least 90%. We believe that demonstrating ≥90% completion for 1HP, significantly greater than with 3HP, will provide compelling evidence for clinical practice and treatment guidelines to change.

Based on these assumptions, with an average cluster (household contacts of the index case) size of nearly 2, and an outcome coefficient of variation (CV) of 0.15, near the mean bound using the maximum entropy distribution (Chatfield 2020), a sample size of 250 individuals per arm, 500 total, would provide 80% power for detecting a significant difference at a two-sided 0.05 significance level. The sample size is not inflated for losses to follow up, as those who fail to complete the protocol will have reached a study endpoint.

Assumptions, Outcome 2:

Proportion of participants experiencing Grade 2 or higher targeted safety events with 3HP = 13%.

In TBTC Study 26 and the iAdhere Study, rates of drug-related adverse events with 3HP were 7-8%. In clinical cohort studies, rates of discontinuation for adverse events have been higher. An analysis of patients taking 3HP in Taiwan found that treatment discontinuation due to adverse events occurred in 12%. (Chan, ERJ 2018) A study of health care workers in South Korea reported that 19% of those receiving 3HP had hypersensitivity reactions and 15% discontinued 3HP due to drug reactions. (Jo, in press) We conservatively assume the rate will be 13%.

Proportion of participants experiencing Grade 2 or higher targeted safety events with 1HP = 6%.

In the BRIEF TB trial the rate of targeted safety events was 3%. We would want to demonstrate a reduction from 13% to at least as low as 6% in HIV-negative patients. With a CV of 0.1 (again, in reference to the maximum entropy distribution for the corresponding proportions, which are lower here than for the previous outcome), and the sample size of 250 individuals (about 125 households) in each study arm, we will have about 80% power for detecting a significant difference at a two-sided 0.05 significance level. We would also have 80% power to distinguish between risks of 12% and 5% in the two study arms. Although for convenience we have phrased this in terms of an hypothesis test, our goal is to estimate the difference in safety risks. With this sample size, and risks of 13% and 6% with 3HP and 1HP, respectively, the 95% CI for the risk difference will have a half-width of 3 percentage points. Reporting this CI will enable readers to make their own conclusions regarding non-inferiority of 1HP with respect to 3HP, in addition to superiority. (Dunn 2018)

15.5 Enrollment/Stratification/Randomization

This is a randomized trial. Trial randomization will be computer-generated by the study's central data manager at Johns Hopkins University School of Medicine. Eligible patients who meet all of the inclusion criteria and none of the exclusion criteria will be randomized in a 1:1 ratio to the study arms. Contacts of the same index case will be considered a cluster and assigned to the same treatment arm according to the randomization of the first enrolled contact. Cluster (contacts of an index patient) size is limited to 2 participants per index case due to the diminishing returns in terms of power per enrollee within a household.

Randomization will be stratified by study site. After household contacts have signed informed consent forms, they will be randomized as a cluster to the same study arm, i.e. all receiving the same regimen. Other household members subsequently identified will not be eligible for the study.

The study is unblinded for practical reasons owing to differing pill counts between the arms and adherence being essential to the primary outcome.

15.6 Maintaining Trial Treatment Randomization Codes

The Johns Hopkins data manager will create a randomization sequence using STATA.

To prevent selection bias in the assignment to treatment the allocation sequence will be concealed from those assigning participants to intervention groups, until the moment of assignment.

15.7 Participant Enrollment and Follow-up

See Section 12.1.3

15.8 Data and Safety Monitoring

A Data and Safety Monitoring Board (DSMB) will review the study protocol and oversee progress of the trial at least annually. DSMB representatives will be determined by JHU who will manage the DSMB, but will include an independent statistician and at least one clinician who is experienced in the field of TB care.

The DSMB will meet at the following time points: Organizational Meeting, scheduled review meeting for interim analysis, annually, ad hoc meeting to address a specific safety concern, and a final meeting.

15.8.1 Planned Interim Analysis and Stopping Guidelines

After 25 participants in Arm A have completed 4 weeks treatment (28th HP dose), and 25 participants from ARM B have completed 4 weeks treatment (their 4th HP dose), we will conduct an interim safety and adherence assessment to ensure that 1HP is safe and well tolerated by HIV-negative participants, as compared with 3HP.

No early stopping rules will be formally adopted for this Phase IV trial.

Enrollment will not pause during the interim analysis. However, when participants from 80 households have completed 16 weeks on-study, a masked assessment of the within-household correlation will be made to verify sample size assumptions.

15.8.2 Interim Efficacy Review

No interim analysis for efficacy is planned.

15.8.3 Analysis Plan

Analyses will be intention-to-treat for both primary outcomes of successful completion and safety. For the successful completion outcome, losses to follow up (at 4 weeks for Arm 1HP and 12 weeks for Arm 3HP) will be considered as unsuccessful; a sensitivity analysis will be conducted among those not lost in both arms as of 12 weeks. For the safety outcome as of 24 weeks, losses will be considered as failures (safety events).

For the primary outcomes, if fewer than 5% of failures occur in households with 2 or more failures, p-values will be calculated using Fisher’s exact test, and 95% confidence intervals (CI) for risk differences will be calculated using the standard asymptotic formula.
Otherwise, within-household correlation will be accounted for using

generalized estimating equations with logit link (for p-value) or identity link (for risk difference CI) and exchangeable correlation structure. If there are convergence problems, a log link function will be used, with delta-method-constructed confidence intervals.

Incidence of active tuberculosis will only be assessed during six months following enrollment but will be evaluated as an exploratory outcome. Incidence will be compared using Kaplan-Meier estimates at 24 weeks and Greenwood's variance formula. This will be a sufficiently rare event that within-household correlation will be ignorable. Censoring will be at last study visit or death. As a sensitivity analysis, TB-free survival will also be analyzed.

TAM studies for the Ultra Curto study are designed to capture the amount of time needed to provide each unit of clinical and/or laboratory services to the patients. Sampling for TAM data collection will be stratified to ensure a broad coverage of visits by time of day, day of week, and time of year, plus inclusion of participants who require additional clinical evaluation (e.g., for side effects) so that the data represents a range of clinical operational conditions in our study setting. The data from TAM study will be analyzed and reported as unit time per 1) each discrete activity specified (clinical, laboratory, and treatment management) in Table 1 and 2) assessed as total health care staff person-time required (reported by types of personnel) for one patient throughout his/her LTBI care cascade (from initial diagnosis and clinical evaluations to treatment completion, inclusive of management of side effects). These time estimates will include both the direct (e.g. patient's face-to-face interaction with health care staff) and indirect (e.g. administrative efforts such as chart reviews and data reporting) efforts relevant for programmatic component of the patient event schedules of the Ultra Curto trial."

15.8.4 Economic Analysis Plan

The economic analysis plan will be submitted as a separate companion study to this main trial.

16. DATA HANDLING AND RECORDKEEPING

16.1 Data Management Responsibilities

Data handling and record keeping will be performed by the site, under the supervision of the site PI in accordance with procedures that are documented in the site's detailed Data Management Operations Manual.

Each participant will be assigned a unique study ID number. This number will be recorded on each data collection form and clinical specimen. Personally

identifiable information including names will not be used on data collection forms. All study records will be stored in locked files in a secure area and access will be limited to study personnel and designated regulatory personnel. All forms will be reviewed prior to data entry for accuracy, consistency, and completeness by designated study staff.

Statistical analysis will be done as a joint effort between JHU Investigators and the site PI, using JHU statisticians.

16.2 Essential/Source Documents and Access to Source Data/Documents

Appropriate records will be maintained for this trial, in compliance with ICH E6 GCP, and regulatory and institutional requirements for the protection of confidentiality of participants. Study records (source documents, signed informed consent forms, IRB/IEC correspondence and approval letters, and screening logs) will be kept in a secure location accessible only to authorized study staff. The following will have access to study records: members of the study team; IRBs that review the study, the Office of Human Research Protections, the US FDA, EMEA, the NIAID, and other local, US, and international regulatory entities. Authorized representatives of the Sponsor and regulatory agencies will be permitted to examine study records for QA reviews, audits, and evaluation of the study safety and progress.

Secure archives are available on-site for preliminary storage after study closure, before moving them to an off-site, secure storage facility. Study records will be maintained by the investigator following study discontinuation in accordance with all applicable NIH and local regulatory requirements in Brazil.

16.3 Quality Control and Quality Assurance

Sites will submit a detailed quality management plan prior to study-start. QC and QA will be conducted according to site-specific SOPs.

Data will be validated on entry, using range and consistency checks. Quality control procedures will include review of CRFs for completion and correctness and source data verification. The study will be monitored by internal and external monitors. The study site will provide direct access to all trial-related facilities, source documents, and reports for the purpose of monitoring and auditing by the Sponsor, and inspection by regulatory authorities. Following written procedures, study monitors will verify that the clinical trial is conducted and data are generated, documented (recorded), and reported in compliance with the protocol and the applicable regulatory requirements.

17. CLINICAL SITE MONITORING

Site Monitoring will be conducted to ensure that human subject protection, study procedures, laboratory procedures, study intervention administration, and data collection processes are of high quality and meet Sponsor, JHU, Health Department (Rio de Janeiro)/FMT(Manaus) and regulatory guidelines, and that the study is conducted in accordance with the protocol and site SOPs. Site monitoring will be performed according to details in a written monitoring plan.

The study will be monitored internally at regular intervals according to site specific SOPs. Westat will conduct periodic external monitoring.

The following will be monitored by internal and external reviewers:

100% Source Data Verification for all enrolled participants.
For these participants, the following will be conducted:

- Informed Consent Form (ICF) review for 100% of participants.
- On-site source data verification (SDV) of source documents: paper source versus electronic Case Report Form (eCRF)
- Eligibility criteria (full eligibility will be assessed for 100% of enrolled participants)
- Review of randomization documentation and verification of correct treatment arm allocation
- Monitoring of Investigational Product (IP) accountability and pharmacy documentation for all enrolled participants.
- Monitoring of safety laboratory results, requisition forms and shipping documentation
- Verifying that all biological samples have been collected and stored (e.g. urine, blood, and dried blood spot samples) as per protocol
- Sites' pharmacies to review product storage and management

18. HUMAN SUBJECTS PROTECTIONS

18.1 Institutional Review Board/Ethics Committee

This protocol, the informed consent document (Appendix VI), and any subsequent modifications must be reviewed and approved by the IRB or Ethics Committee (s) (EC) responsible for overseeing the study.

18.2 Vulnerable Participants

18.2.1 Pregnant participants and fetuses

This trial excludes pregnant participants

18.2.2 Prisoners

This trial excludes prisoners and individuals with known impending incarceration.

18.2.3 Children

For potential participants under 18 years of age, the assent of the child as well as the written informed consent of the child's legal guardian will be required for enrollment in this study. The child will receive, in language appropriate to the age and maturity of the child, an explanation of the research procedures; a description of the risks, discomforts, or inconveniences that the child might experience; and assurance that the child can withdraw from the study at any time.

The assent process will be conducted by a study staff member who is experienced in consent and assent procedures, and in accordance with IRB/IEC requirements. All study participants age < 18 years will provide oral or written assent and written consent by the participant's legal guardian, in accordance with national and/or local IRB/IEC requirements.

Participants who reach age 18 will be consented on the adult ICF at their next visit.

18.2.4 Illiterate participants

Illiterate participants will be consented by a trained research team member in the presence of an independent adult third party witness who is chosen by the participant, and is not a member of the research team.

The participant will make her or his mark or thumbprint as signature, and the witness will sign in the informed consent document, noting date and time of signature.

18.3 Informed Consent

Informed written consent and assent, where applicable, using IRB-approved consent forms, will be obtained by trained study personnel prior to performing study-specific procedures. Potential participant's and/or their parent/guardian will receive information about risks and possible benefits of study participation, study objectives and procedures, potential toxicities, and the informed consent process.

Informed consent requires the signature or mark of the participant and/or parent/ guardian if the participant is under age 18 years. A copy of the signed and dated informed consent document will be offered to participants for their records.

The rights and welfare of the subjects will be protected by emphasizing that the quality of the participant's medical care will not be adversely affected if they decline to participate in this study, and that they may withdraw consent at any time. If the choice is made not to participate in the study, the patient will be referred to local sources of TB care.

18.3.1 Informed Consent Process

This study will be conducted according to the ethical principles set forth in the Declaration of Helsinki, ICH-GCP, and local regulatory requirements as applicable.

Only individuals who provide written informed consent will be enrolled in this study. Written informed consent is required before any study-specific procedures are performed.

In an area of the facility that preserves confidentiality, trained study staff will explain the conditions of the study including potential harms and benefits, the nature and timing of study procedures, alternatives to study participation, that study participation is voluntary, that a decision to not participate in the study will not affect the quality of their future medical care, and that they may withdraw from participation at any time. The information in the Informed Consent document will be translated into relevant local languages.

Literate individuals will be provided with a language-appropriate document to read; illiterate individuals (i.e. individuals who speak and understand, but do not read and write, the language in which the consent discussion is conducted) will have the contents of the document explained to them by a trained study staff member; such individuals can be enrolled by 'making their mark' or thumbprint on the consent document.

Potential participants will have the opportunity to ask questions of the site investigator or delegate, and to discuss participation with their family and/or friends or think about the study prior to deciding whether to participate. A copy of the signed informed consent document will be given to the participant for his/her records.

18.3.2 Assent Process

For potential participants under 18 years of age, the assent of the child as well as the written informed consent of the child's legal guardian will be required for enrollment in this study. The child will receive, in language appropriate to the age and maturity of the child, an explanation of the research procedures; a description of the risks, discomforts, or inconveniences that the child might experience; and assurance that the child can withdraw from the study at any time.

The assent process will be conducted by a study staff member who is experienced in consent and assent procedures, and in accordance with IRB/IEC requirements. All study participants age < 18 years will provide oral or written assent and written consent by the participant's legal guardian, in accordance with national and/or local IRB/IEC requirements.

A participant who reaches age 18 during the study will be consented on the adult ICF at the next study visit.

18.3.3 Documenting Informed Consent

The informed consent and assent process will be documented in a narrative source note as described in the MOP.

Original, signed informed consent documents will be stored in an informed consent master binder, with a copy filed in each participant's source document binder. A third copy will be given to the participant or held separately in a secured area for the participant.

18.3.4 Stored samples

This study does not create a biobank or contribute to an existing biobank. Dried blood spots for qualitative drug levels and urine specimens will be stored on site temporarily for batched runs in Brazil and the USA.

18.4 Risks and SARS-CoV-2 Precautions

The 3HP regimen has been approved by the US FDA and is recommended by WHO for TB preventive therapy. The potential benefits of taking 3HP is a reduced risk of developing TB disease. The 3HP regimen has been found to be safe in other studies. In a recent DTG-3HP PK study, 2 of 3 participants developed a hypersensitivity syndrome. The potential harm to participants may include developing drug induced adverse reaction, particularly hypersensitivity reactions due to RPT.

1HP has demonstrated safety and tolerability during a trial in PLWH. This is the first trial of 1HP in HIV un-infected individuals.

Risks associated with study participation include the possibilities that an investigational regimen has efficacy that is inferior to the standard regimen and/or an investigational regimen is more toxic than the standard regimen, and confidentiality risks.

The following measures will minimize risk to participants: individual participants will be closely monitored clinically, and a Data and Safety Monitoring Board will review trial progress at scheduled and *ad hoc* intervals.

The investigational regimens could prove to have greater toxicity than the standard regimen. This is unlikely given that toxicity data collected in the

BRIEF TB/A5279 clinical trial showed that 1HP administered to PLWH at a once daily weight-stratified dose of rifapentine plus isoniazid for four weeks was well tolerated. However, this is the first 1HP trial to include HIV-uninfected participants.

Confidentiality risks will be minimized through the measures described in Section 18.

SARS-CoV-2 Precautions

As of July 2020, Brazil is experiencing widespread community transmission of SARS-CoV-2, the cause of COVID-19 disease. The study team will take steps necessary to protect study participants and staff from the risk of becoming infected with SARS-CoV-2 because of participation in this protocol. The study will provide personal protective equipment to the study staff, if needed.

I. Opening and closing of the protocol

a. The trial will be opened to accrual when specific permission is obtained from local IRBs in Rio de Janeiro and Manaus, and when local and national health guidelines allow clinical research to be undertaken.

b. The study team will suspend recruitment into the trial when advised by local and national health authorities, or when the study team determines that recruitment of participants could jeopardize the health of study staff and participants. Reopening of the trial would occur when the conditions in part I.a., above, have been met.

II. Management of enrolled participants should a shutdown be necessary.

a. Participants receiving study medications would be advised to continue treatment until complete. Medication refills would be delivered to participants either at the study clinic or by mail.

b. Safety monitoring would be conducted through telephone, WhatsApp, or other remote methods. Safety labs would be

collected, if possible, either by allowing participants to visit study clinics or by home visits by phlebotomists. In both circumstances strict infection control guidelines would be followed, including use of personal protective equipment by both staff and participants. If safety and monitoring labs could not be carried out, participants could continue medication with remote monitoring only, given that both study regimens are WHO-approved and known to be generally safe.

c. In the absence of SARS-CoV-2 testing it is conceivable that AEs that are due to COVID-19 could be wrongly attributed to study interventions (especially fever, nausea, and increased LFTs). In the event that a study participant developed such symptoms, study staff would facilitate access to SARS-CoV-2 testing as part of evaluating the possible AE.

18.5 Social Impact Events

Individuals enrolled in this study may experience personal problems resulting from the study participation. Such problems are termed social impact events. Although study sites will make every effort to protect participant privacy and confidentiality, it is possible that participants' involvement in the study could become known to others, and that participants may experience stigmatization or discrimination as a result of being perceived as being HIV- infected or at risk for HIV infection. For example, participants could be treated unfairly, or could have problems being accepted by their families and/or communities. Problems may also occur in circumstances in which study participation is not disclosed, such as impact on employment related to time taken for study visits.

In the event that a participant reports a social impact event, every effort will be made by study staff to provide appropriate assistance, and/or referrals to appropriate resources. Social impact events are documented and reviewed on a scheduled basis by the protocol team leadership with the goal of reducing their incidence and enhancing the ability of study staff to mitigate them when possible.

Social impact events that are judged by the IoR/designee to be serious, unexpected, or more severe or frequent than anticipated, will be reported to the responsible site's EC/IRB promptly, or otherwise in accordance with the EC/IRB's requirements.

18.6 Benefits

Both treatment regimens are offered with therapeutic warrant, meaning that it is reasonable to expect that these TPT treatments will be safe and tolerated. The study is not powered for efficacy of preventing tuberculosis.

Study participants will benefit indirectly as it is well established that outcomes for participants in LTBI trials are better than those for patients receiving routine care. This study will benefit society by contributing to the understanding of optimal strategies for preventing tuberculosis.

18.7 Compensation

Participants in the study, regardless of treatment arm, will receive compensation for time and travel expenses required to attend scheduled study visits. Compensation will be in compliance with the local regulation.

18.8 Participant Privacy and Confidentiality

All participant-related information including case report forms, laboratory specimens, evaluation forms, reports, etc., will be kept strictly confidential. All records will be kept in a secure, double locked location and only research staff will have access to the records.

Participants will be identified only by means of a coded number specific to each participant. All computerized databases will identify participants by numeric codes only, and will be password-protected. Upon request, participant records will be made available to the study sponsor, the sponsor's monitoring representative, representatives of a participating pharmaceutical sponsor and applicable regulatory entities.

18.9 Certificates of Confidentiality

Certificates of Confidentiality protect the privacy interests of research participants and are limited to information that is sent to the U.S. The NIH issues Certificates of Confidentiality are part of NIH funded grant awards, such as this trial.

18.10 Critical Event Reporting

Critical event reporting is described in Section 13.

18.11 Communicable Disease Reporting

Any findings involving a communicable disease will be reported to the appropriate health authorities per Brazil's local and national requirements.

18.12 New Findings

New findings will be reported in the DSMB report, if applicable, and/or in subsequent reports, abstracts, and publications. Participants will be informed of any new findings that could affect their willingness to join the study or continue participating.

18.13 Study Discontinuation

The study may be discontinued at any time by the sponsor, investigators, DSMB, or by any of the relevant local, national, and international regulatory authorities.

18.14 Post-Trial Access

Post trial access is not applicable, because complete TPT regimens are provided in the study period.

18.15 Community Advisory Board and Other Relevant Stakeholders

The protocol will be reviewed and approved by the local institutions Research Ethics Committee (CEP) in Rio de Janeiro and Manaus and the National Health Research Commission (CONEP) and the Johns Hopkins School of Medicine (IRB).

19. ADMINISTRATIVE PROCEDURES

19.1 Protocol Registration

The study will be registered with the DMID Protocol Registration Office, if applicable.

19.2 Regulatory Oversight

The study will not operate under an IND.

19.3 Study Implementation

19.3.1 Investigator team

The investigator team of collaborators will meet at least semi-monthly by teleconference to discuss implementation progress. The meetings will be chaired by the PI.

19.3.2 Project and site implementation teams

The core project management team will oversee project implementation. The team will include the PI, Project Manager, Site Investigators, Study Coordinator, and Data Manager. The project management team will meet every 2 weeks by teleconference or face to face.

The Site implementation team will be comprised of the site investigators, clinic coordinators, and recruiters responsible for the implementation of the project at trial sites. The site implementation team will oversee the day-to-day operational aspects of the study at the trial site.

19.4 ClinicalTrials.gov

This protocol is subject to the Food and Drug Administration Amendments Act of 2007 (FDAAA) and will be registered in ClinicalTrials.gov.

20. PUBLICATION POLICY

Publication of the results of this trial will occur during the project, if appropriate, or at the end of the project, consistent with normal scientific practices. The results of the study will be submitted for publication in peer-reviewed scientific journals. Publication will occur in accordance with International Committee of Medical Journal Editors and Indian Council of Medical Research guidance, among others.

21. REFERENCES

Abu-Raddad LJ, Sabatelli L, Achterberg JT, Sugimoto JD, Longini IM Jr., Dye C, Halloran ME. Epidemiological benefits of more-effective tuberculosis vaccines, drugs, and diagnostics. Proc Natl Acad Sci USA 2009;106:13980-5.

Baciewicz AM, Self TH. Isoniazid interactions. South Med J. Jun 1985;78(6):714-718.

Belknap R, Holland D, Feng PJ, et al., TB Trials Consortium iAdhere Study Team. Self-administered Versus Directly Observed Once-Weekly Isoniazid and Rifapentine Treatment of Latent Tuberculosis Infection: A Randomized Trial. Ann Intern Med. 2017;167:689-697.

Bemer-Melchior P, Bryskier A, Drugeon HB. Comparison of the in vitro activities of rifapentine and rifampicin against Mycobacterium tuberculosis complex. J Antimicrob Chemother. 2000;46:571-6.

Blakemore R, Nabeta P, Davidow AL, Vadwai V, Tahirli R, Munsamy V, Nicol M, Jones M, Persing DH, Hillemann D, Ruesch-Gerdes S, Leisegang F, Samudio C, Rodrigues C, Boehme CC, Perkins MD, Alland D. A multisite assessment of the quantitative capabilities of the Xpert MTB/RIF assay. Am J Respir Crit Care Med. 2011;184:1076-84.

Brooks KM, George JM, Pau AK, Rupert A, et al. Cytokine-mediated systemic adverse reactions In a drug-drug interaction study of dolutegravir with once-weekly isoniazid and rifapentine. Clin Infect Dis. 2018; 67(2): 193-201.

Burman WJ, Cotton MF, Gibb DM, Walker AS, Vernon AA, Donald PR. Ensuring the involvement of children in the evaluation of new tuberculosis treatment regimens. PLoS Med 2008; 5(8):e176:1168-1172.

Centers for Disease Control and Prevention (CDC). Update: Fatal and severe liver injuries associated with rifampin and pyrazinamide for latent tuberculosis infection, and revisions in American Thoracic Society/CDC recommendations— United States,

2001. MMWR Morb Mortal Wkly Rep. 2001 Aug 31;50(34):733-5.

Centers for Disease Control and Prevention. Recommendations for use of an isoniazid-rifapentine regimen with direct observation to treat latent Mycobacterium tuberculosis infection. MMWR Morb Mortal Wkly Rep. 2011;60(48):1650-1653.

Chatfield MD, Farewell DM. Understanding between-cluster variation in prevalence, and limits for how much variation is plausible. Stat Meth Med Res. 2020 (in press).

Corbett EL, Watt CJ, Walker N, Maher D, Williams BG, Raviglione MC, Dye C. The growing burden of tuberculosis: global trends and interactions with the HIV epidemic. Arch Intern Med. 2003;163:1009-21.

Dooley KE, Bliven-Sizemore EE, Weiner M, Lu Y, Nuermberger EL, Hubbard WC, Fuchs EJ, Melia MT, Burman WJ, Dorman SE. Safety and pharmacokinetics of escalating daily doses of the antituberculosis drug rifapentine in healthy volunteers. Clin Pharmacol Ther. 2012;91:881-8.

Dorman SE and the Tuberculosis Trials Consortium. Antimicrobial activity and safety of high-dose rifapentine-containing regimens for treatment of pulmonary TB: Study 29X of the CDC Tuberculosis Trials Consortium. International Conference of the American Thoracic Society, Philadelphia, PA, 17-22 May 2013.

Dunn DT, Copas AJ, Brocklehurst P. Superiority and non-inferiority: two sides of the same coin? Trials 2018; 19:499.

Durovni B, Cavalcante SC, Saraceni V, Vellozo V, Israel G, King BS, et al. The implementation of isoniazid preventive therapy in HIV clinics: the experience from the TB/HIV in Rio (THRio) study. AIDS 2010 Nov;24 Suppl 5:S49-56.

Ellard GA. The potential clinical significance of the isoniazid acetylator phenotype in the treatment of pulmonary tuberculosis. Tubercle. Sep 1984;65(3):211-227.

Gillespie SH, Crook AM, McHugh TD, Mendel CM, Meredith SK, Murray SR, Pappas F, Phillips PP, Hirsch-Moverman Y, Daftary A, Franks J, Colson PW. Adherence to treatment for latent tuberculosis infection: systematic review of studies in the US and Canada. Int J Tuberc Lung Dis 2008 Nov;12(11):1235-1254.

Gordin F, Chaisson RE, Matts, JP, Miller C, de Lourdes Garcia M, Hafner R, et al. Rifampin and Pyrazinamide vs isoniazid for prevention of tuberculosis in HIV-infected persons. JAMA 2000;283:1445-50.

Guerra RL, Conde MB, Efron A, et al. Point-of-care Arkansas method for measuring adherence to treatment with isoniazid. Respiratory medicine. 2010;104(5):754-7.10.1016/j.rmed.2010.02.001

Halsey NA, Coberly JS, Desormeaux J, Losikoff P, Atkinson J, Moulton LH, et al. Randomised trial of isoniazid versus rifampin and pyrazinamide for prevention of tuberculosis in HIV-1 infection. Lancet 1998;351:786-92.

Hibma JE, Radtke KK, Dorman SE, et al. Rifapentine Population Pharmacokinetics and Dosing Recommendations for Latent Tuberculosis Infection [published online ahead of print, 2020 May 15]. *Am J Respir Crit Care Med*. 2020;10.1164/rccm.201912-2489OC. doi:10.1164/rccm.201912-2489OC

Husereau D, Drummond M, Petrou S, Carswell C, Moher D, Greenberg D, Augustovski F, Briggs AH, Mauskopf J, Loder E. Consolidated health economic evaluation reporting standards (CHEERS) statement. Cost Effectiveness and Resource Allocation. 2013 Dec;11(1):6.

Kay L, Kampmann JP, Svendsen TL, et al. Influence of rifampicin and isoniazid on the kinetics of phenytoin. Br J Clin Pharmacol. Oct 1985;20(4):323-326.

Kendall EA, Durovni B, Martinson NA, et al. Adherence to tuberculosis preventive therapy measured by urine metabolite testing among people with HIV. AIDS, in press.

Langdon G, Wilkins JJ, Smith PJ, McIlleron H. Consecutive-dose pharmacokinetics of rifapentine in patients diagnosed with pulmonary tuberculosis. Int J Tuberc Lung Dis 2004 Jul;8(7):862-867.

Maroni M. Integrative Assessment to Predict Juvenile Toxicity Risk for Rifapentine. Personal communication, November 12, 2014

Marshall JD, Abdel-Rahman S, Johnson K, Kauffman RE, Kearns GL. Rifapentine pharmacokinetics in adolescents. Pediatr Infect Dis J. 1999;18: Marshall JD, Abdel-Rahman S, Johnson K, Kauffman RE, Kearns GL. Rifapentine pharmacokinetics in adolescents. Pediatr Infect Dis J. 1999;18:882-8.882-8.

Martinson NA, Barnes GL, Moulton LH, Msandiwa R, Hausler H, Ram M, et al. New regimens to prevent tuberculosis in adults with HIV infection. N Engl J Med. 2011;365(1):11-20.

McClintock AH, Eastment M, McKinney CM, Pitney CL, Narita M, Park DR, et al. Treatment completion for latent tuberculosis infection: a retrospective cohort study comparing 9 months of isoniazid, 4 months of rifampin and 3 months of isoniazid and rifapentine. BMC Infect Dis 2017 Feb 14;17(1):146-017-2245-8.

McKenna L, Barnabas N, Seaworth B, Theunissen M, Clayden P, Nachman S, Furin J, Becerra M. The missing cohort: adolescents in tuberculosis research. Oral presentation, 45th Union World Conference on Lung Health, Barcelona, Spain, October, 2014.

Ormerod LP, Horsfield N. Frequency and type of reactions to antituberculosis drugs: observations in routine treatment. Tuber Lung Dis. Feb 1996;77(1):37-42.

Pantoja A, Fitzpatrick C, Vassall A, Weyer K, Floyd K. Xpert MTB/RIF for diagnosis of tuberculosis and drug-resistant tuberculosis: a cost and affordability analysis. European Respiratory Journal. 2013 Sep 1;42(3):708-20.

Parsons TL, Marzinke MA, Hoang T, et al. Quantification of Rifapentine, A Potent Antituberculosis Drug, from Dried Blood Spot Samples Uusing Lliquid Chromatographic-Tandem Mass Spectrometric Aanalysis. Antimicrob Agents Chemother. 2014;58(11):6747-57.10.1128/aac.03607-14

Patel AM, McKeon J. Avoidance and management of adverse reactions to antituberculosis drugs. Drug Saf. Jan 1995;12(1):1-25.

Peloquin CA, Namdar R, Dodge AA, Nix DE. Pharmacokinetics of isoniazid under fasting conditions, with food, and with antacids. Int J Tuberc Lung Dis. Aug 1999;3(8):703-710.

Peloquin CA, Namdar R, Singleton MD, Nix DE. Pharmacokinetics of rifampin under fasting conditions, with food, and with antacids. Chest 1999;115:12-18.

Priftin (rifapentine) package insert. Sanofi Aventis, 2018.

Salomon JA, Haagsma JA, Davis A, de Noordhout CM, Polinder S, Havelaar AH, Cassini A, Devleesschauwer B, Kretzschmar M, Speybroeck N, Murray CJ, Vos T. Disability weights for the Global Burden of Disease 2013 study. Lancet Glob Health. 2015 Nov;3(11):e712-23.

Sanders GD, Neumann PJ, Basu A, Brock DW, Feeny D, Krahn M, Kuntz KM, Meltzer DO, Owens DK, Prosser LA, Salomon JA. Recommendations for conduct, methodological practices, and reporting of cost-effectiveness analyses: second panel on cost-effectiveness in health and medicine. Jama. 2016 Sep 13;316(10):1093-103.

Savic RM, Weiner M, MacKenzie WM, et al., Defining the Optimal Dose of Rifapentine for Pulmonary Tuberculosis: Exposure–Response Relations From Two Phase II Clinical Trials. Clin Pharmacol Ther 2017;102:321-31.

Schechter M, Zajdenverg R, Falco G, Barnes GL, Faulhaber JC, Coberly JS, et al. Weekly rifapentine/isoniazid or daily rifampin/pyrazinamide for latent tuberculosis in household contacts. Am J Respir Crit Care Med 2006 Apr 15;173(8):922-926.

Snider DE Jr. Pyridoxine supplementation during isoniazid therapy. Tubercle. 1980;61:191-6.

Sterling TR, Villarino, ME, Borisov AS, et al. Three months of once weekly rifapentine and isoniazid for M. tuberculosis infection. N Engl J Med 2011;365:2155-66.

Sterling TR, Scott NA, Miro JM, Calvet G, La Rosa A, Infante R, et al. Three months of weekly rifapentine and isoniazid for treatment of Mycobacterium tuberculosis infection in HIV-coinfected persons. AIDS 2016 Jun 19;30(10):1607-1615.

Sullivan SD, Mauskopf JA, Augustovski F, Caro JJ, Lee KM, Minchin M, Orlewska E, Penna P, Barrios JM, Shau WY. Budget impact analysis—principles of good practice: report of the ISPOR 2012 Budget Impact Analysis Good Practice II Task Force. Value in health. 2014 Jan 1;17(1):5-14.

Swindells S, Ramchandani R, Gupta A, et al. One Month of Rifapentine and Isoniazid to Prevent HIV-related Tuberculosis. N Engl J Med 2019; 380:1001-1011

Venkatesan K. Pharmacokinetic drug interactions with rifampicin. Clin Pharmacokinet. 1992;22:47-65.

Villarino ME, Scott NA, Weis SE, Weiner M, Conde MB, Jones B, Nachman S, Oliveira R, Moro RN, Shang N, Goldberg SV, Sterling TR. Treatment for preventing tuberculosis in children and adolescents: a randomized clinical trial of a 3-month, 12 dose regimen of rifapentine and isoniazid. JAMA Pediatr. 2015 Mar;169(3):247- 55

Wayne LG, Hayes LG. An in vitro model for sequential study of shiftdown of Mycobacterium tuberculosis through two stages of nonreplicating persistence. Infect Immun 1996;64:2062-9.

Wayne LG, Sohaskey CD. Nonreplicating persistence of Mycobacterium tuberculosis. Annu Rev Microbiol 2001;55:139-63.

WHO. Latent Tuberculosis Infection: Updated and Consolidated Guidelines for Programmatic Management. Geneva, 2018. License: CC BY-NC-SA 3.0 IGO

World Health Organization. WHO operational handbook on tuberculosis. Module 1: prevention - tuberculosis preventive treatment. Geneva: World Health Organization; 2020. Licence: CC BY-NC-SA 3.0 IGO.

Yee D, Valiquette C, Pelletier M, Parisien I, Rocher I, Menzies D. Incidence of serious side effects from first-line antituberculosis drugs among patients treated for active tuberculosis. Am J Respir Crit Care Med. Jun 1 2003;167(11):1472-1477.

Zent C and Smith P. Study of the effect of concomitant food on the bioavailability of rifampicin, isoniazid, and pyrazinamide. Tubercle and Lung Dis. 1995;76:109- 113.

Zierski M, Bek E. Side-effects of drug regimens used in short-course chemotherapy for pulmonary tuberculosis. A controlled clinical study. Tubercle. Mar 1980;61(1):41-49.

Zvada SP, Van Der Walt JS, Smith PJ, et al. Effect of four different meal types on the population pharmacokinetics of single dose rifapentine in healthy male volunteers. Antimicrob Agents Chemother. 2010;54:3390-4.

22. APPENDICES

I Schedule of events and study duration
II Sample study medication & food diary
III Rifapentine hypersensitivity reaction worksheet
IV List of Precautionary Medications
V *Division of AIDS (DAIDS) Table for Grading Adult and Pediatric Adverse Events, Corrected Version 2.1* (dated July 2017)

APPENDIX I
Table 1A: Schedule of evaluations (SOE) for Arm A

1HP	Screening	Enrolment	On-study period*					
Study week	***	0	2	4	8	12	16	24
CLINICAL EVALUATIONS								
Informed Consent	X							
History	X	X						
Concomitant med list	X	X	X	X	X	X	X	X
TB symptom screen	X	X	X	X	X	X	X	X
Physical exam (complete)	X							
Physical exam (targeted)		X	X	X	X	X	X	X
Adverse event assessment			X	X	X	X	X	X
Adherence Questionnaire			X	X				
Pill count			X	X				
LABORATORY EVALUATIONS								
Hematology	X		X	X				
Chemistry, including LFTs	X		X	X				
Hepatitis B surface antigen (HBsAg)	X							
Pregnancy test	X	X**	X	X	X	X	X	X
HIV-1 antibody test (Documented)	X							
TB DIAGNOSTICS								
Chest x-ray	X							
TST or Interferon gamma release assay (IGRA)	X							
PHARMACOLOGY								
Sampling for rifapentine (dried blood spot)			X	X				
Sampling for isoniazid (urine)			X	X				
Sampling for NAT2 gene			X					
STUDY DRUG DOSING								
Isoniazid/rifapentine (once **daily** for **4** weeks)*		X	X	X				
COSTING EVALUATIONS								
Patient Costing Survey (50 patients)		X				X		X
Clinic Staff Time-and-Motion****		X	X	X	X	X	X	X

*All study visits will have a visit window of +/-3 days **for individuals of childbearing potential (repeat at enrollment if screening pregnancy test was more than 48 hours prior to enrolment) *** Up to 7 days prior to enrolment **** All visits are eligible, but time-and-motion (TAM) assessments will be conducted at a randomly selected sample of study visits. The TAM assessment involves clinic staff, not study participants. † Within 90 days of screening

Table 1B: Schedule of evaluations (SOE) for Arm B

3HP	Screening	Enrolment	On-study period*					
Study week dose (dose 1 given at week 0)_	***	1	2	3	8	11	16	24
CLINICAL EVALUATIONS								
Informed Consent	X							
History	X	X						
Concomitant med list	X	X	X	X	X	X	X	X
TB symptom screen	X	X	X	X	X	X	X	X
Physical exam (complete)	X							
Physical exam (targeted)		X	X	X	X	X	X	X
Adverse event assessment			X	X	X	X	X	X
Adherence Questionnaire			X	X	X	X		
Pill count			X	X	X	X		
LABORATORY EVALUATIONS								
Hematology	X		X	X	X	X		
Chemistry, including LFTs	X		X	X	X	X		
Hepatitis B surface antigen (HBsAg)	X							
Pregnancy test	X	X**	X	X	X	X	X	X
HIV-1 antibody test (Documented)	X							
TB DIAGNOSTICS								
Chest x-ray	X							
TST or Interferon gamma release assay (IGRA)	X							
PHARMACOLOGY								
Sampling for rifapentine (dried blood spot)				X		X		
Sampling for isoniazid (urine)				X		X		
NAT2 gene				X				
STUDY DRUG DOSING								
Isoniazid/rifapentine (once **weekly** for **12** weeks)*		X	X	X	X	X		
COSTING EVALUATIONS								
Patient Costing Survey (50 patients)		X				X		X
Clinic Staff Time-and-Motion****		X	X	X	X	X	X	X

*All study visits will have a visit window of +/-3 days **for individuals of childbearing potential (repeat at enrollment if screening pregnancy test was more than 48 hours prior to enrolment) *** Up to 7 days prior to enrolment **** All visits are eligible, but time-and-motion (TAM) assessments will only be conducted at a randomly selected sample of study visits. The TAM assessment involves clinic staff, <u>not</u> study participants. † Within 90 days of screening

STUDY DURATION

	Year 1				Year 2				Year 3				Year 4				Year 5			
IRB aproval																				
Study site preparation																				
Trainning																				
Enrollment																				
Follow up																				
Health, Economics and Social Data Collection																				
Data analysis																				
Manuscript preparation																				
Final report																				

APPENDIX II

1HP Medication Diary
ARM A

PID ____(sticker)________ Month___________________

ose #	Date of dose	Time of dose	Food and drink taken with HP	Initials
**example**	1 Jan 2020	7:30 am	Rice, beans, 1 egg, coffee with milk	[illegible]
**1**				
**2**				
**3**				
**4**				
**5**				
**6**				
**7**				
**8**				
**9**				
**10**				
**11**				
**12**				
**13**				
**14**				
**15**				
**16**				
**17**				
**18**				
**19**				
**20**				
**21**				
**22**				
**23**				
**24**				
**25**				
**26**				
**27**				
**28**				

Notes:

3HP Medication Diary
ARM B

PID ____(sticker)________

**Dose #**	**Date of dose**	**Time of dose**	**Food and drink taken with HP**	**Initials**
**example**	1 Jan 2020	7:30 am	Rice, beans, 1 egg, coffee with milk	[illegible]
**1**				
**2**				
**3**				
**4**				
**5**				
**6**				
**7**				
**8**				
**9**				
**10**				
**11**				
**12**				
Notes:				

Participants will need a dosing source document for their home TPT doses.

Print medication diary on heavy card stock.
Fold, staple, or tape a blank cover on top to keep the record private.

Distribute the card with the first TPT dose.
Ask/remind participants to bring the card to every visit.

Have the participant fill the card during the first TPT DOT dose in clinic as practice for when s/he will fill the card for home doses.

APPENDIX III

Hypersensitivity Symptom Checklist

Date HP Given: _____/____/_____ Dose #: ________ Time: ____h_____

1. Any symptom/ sign to be followed by a comment and reported to the Investigator on duty who will complete a Hypersensitivity Checklist form

2. Any event recorded on this form MUST also be recorded on the Adverse Event Log

Date	Time	Medical event	Tick if Present	Initial and Comment
		**Hypotension**		
		**Urticaria (Hives)**		
		**Angioedema** ***(swelling around lips/ eyes)***		
		**Acute bronchospasm**		
		**Conjunctivitis** ***(Red, itchy eyes)***		
		**Thrombocytopenia**		
		**Neutropenia**		
		**Anaphylaxis**		
		**Flu-like syndrome:**		
		**Weakness**		
		**Fatigue**		
		**Muscle Pain**		
		**Nausea**		
		**Vomiting**		
		**Headache**		
		**Fever/ Chills**		
		**Body Aches**		
		**Rash**		
		**Itching**		
		**Sweats**		
		**Dizziness**		
		**Shortness of breath**		
		**Chest pain**		
		**Cough**		
		**Syncope**		
		**Palpitations**		

APPENDIX IV

List of Precautionary Medications

Precautionary Agents with INH

Carbamazepine
Chlorzoxazone
Disulfiram
Ketoconazole
Phenytoin
Warfarin
Theophylline
Selective serotonin re-uptake inhibitor antidepressants (e.g. citalopram, fluoxetine, paroxetine, sertraline)

Precautionary Agents with RPT

Medication Class	Precautionary Medications
Antiarrhythmics	Disopyramide
	Mexiletine
	Quinidine
	Tocainide
Antibiotics	Chloramphenicol
	Clarithromycin
	Dapsone
	Doxycycline
	Fluoroquinolones
Anticoagulants	Warfarin
Anticonvulsants	Phenytoin
Antimalarials	Quinine
Antipsychotics	Haloperidol
Azole Antifungals	Fluconazole
	Itraconazole
	Ketoconazole

Medication Class	Precautionary Medications
Barbiturates	Phenobarbital
Benzodiazepines	Diazepam
Beta-Blockers	Propranolol
Calcium Channel Blockers	Diltiazem
	Nifedipine
	Verapamil
Cardiac Glycoside Preparations	Digoxin
Corticosteroids	Prednisone
Fibrates	Clofibrate
Oral hypoglycemic agents	Sulfonylureas
Hormonal Contraceptives/ Progestins	Ethinyl estradiol
	Levonorgestrel
Immunosuppressants	Cyclosporine
	Tacrolimus
Methylxanthines	Theophylline
Narcotic analgesics	Methadone
Phosphodiesterase-5 (PDE-5) Inhibitors	Sildenafil
Thyroid preparations	Levothyroxine
Tricyclic Antidepressants	Amitriptyline
	Nortriptyline

APPENDIX V
DAIDS Grading Table v 2.1

https://rsc.niaid.nih.gov/sites/default/files/daidsgradingcorrectedv21.pdf
