# Supplementary material for: Acceptability and safety of one versus three months of rifapentine and isoniazid to prevent tuberculosis in people exposed in the household or workplace in Brazil: The Ultra-Curto randomized controlled trial
Source: PLoS Med. 2026 Feb 10;23(2):e1004758. doi: 10.1371/journal.pmed.1004758 (PMC12890141; doi:10.1371/journal.pmed.1004758)
Supplement: S2 Text — (PDF) [file pmed.1004758.s005.pdf]

STATISTICAL ANALYSIS PLAN

Full Title:
Treatment Success and Safety of 4 Weeks of Daily Rifapentine and Isoniazid (1HP) vs. 12 weeks of Weekly Rifapentine and Isoniazid (3HP) for Prevention of Tuberculosis in HIV-uninfected Individuals (1v3HP for TPT in HIV-uninfected individuals)

Short Title:
Ultra Curto TPT

Clinical Trial Phase:
Phase IV

ClinicalTrials.gov Identifier: NCT04703075

Version 1. 20 February 2023 L. Moulton & R. Chaisson & R. Thompson

---

Grant number U01A152961 TB study in Brazil (DMID 20-0020)

Pharmaceutical and other Study Product Support Provided by:
Sanofi (Paris, France)

Study Chair and Principal Investigator:
Richard E. Chaisson, MD

Site Principal Investigators:
Betina Durovni, MD, MPH, PhD
Marcelo Cordeiro dos Santos, MD, MSc, PhD
Solange Cavalcante, MD, MSc, PhD

Study Biostatisticians:
Lawrence Moulton, PhD
Silvia Cohn, MS

1. INTRODUCTION

This document contains the statistical analysis plan for the Ultra Curto TPT study. The goal is to avoid data-driven analyses during and at the end of the study to the extent possible. Much of the content of this Statistical Analysis Plan (SAP) is taken directly from the Ultra Curto TPT Study Protocol, version 3.1 of 24 November 2021.

2. BACKGROUND

2.1. Study Overview

This multi-center study compares the treatment success and safety of 3 months of weekly rifapentine and isoniazid (3HP) to one month of daily rifapentine and isoniazid (1HP) in HIV-uninfected adolescents and adults.

3. OBJECTIVES and HYPOTHESES

3.1 Primary Objectives

To compare treatment success of 1HP with 3HP in HIV-uninfected adults and adolescents at increased risk of TB.

To compare the safety of 1HP with 3HP in HIV-uninfected adults and adolescents at increased risk of TB.

3.2 Primary Outcomes and Hypotheses

(1) Treatment success of TBT with ≥90% adherence documented by self-report, pill count, and pharmacologic monitoring.

Hypothesis: Successful treatment with 1HP will be superior to 3HP, with 90% of individuals randomized to 1HP taking >90% of prescribed doses vs 80% of those on 3HP.

(2) Safety of treatment regimens, defined as occurrence of Grade 2 or higher targeted safety events and discontinuation of study medications because of side effects. Targeted safety events are hypersensitivity syndrome, rash, peripheral neuropathy, hepatotoxicity, nausea and vomiting, and drug-related fever.
Hypothesis: We hypothesize that the safety of 1HP will be superior to 3HP, with a rate of targeted adverse events or medication discontinuation of 6% vs. 13% for 3HP.

3.3 Secondary Outcome and Hypothesis

(1) Incremental cost-effectiveness of 1HP and 3HP (compared to each other, 6H, and no treatment) using a societal perspective.

(2) Analyze, in each of the health units in the study, the time needed to provide comprehensive care to patients.

Hypothesis: We hypothesize that 1HP will be cost saving vs 3HP, vs modelled costs of 6H and vs no TPT.

3.4 Exploratory Aims

Incidence of active tuberculosis will be assessed during the six months following enrollment.

4. STUDY DESIGN

This is a multicenter, randomized, controlled, open-label, phase IV clinical trial with two arms, comparing treatment success and safety of 1HP vs 3HP in HIV-uninfected adolescents and adults.

4.1 Study Groups

TB preventive treatment:
Arm A (n=250): Experimental arm. Rifapentine 600 mg daily and isoniazid 300 mg daily for 4 weeks.

Arm B (n=250): Control arm. Rifapentine 900 mg weekly and isoniazid 900 mg weekly for 12 weeks.

Patient Population:

The study will enroll HIV-uninfected individuals who are $\geq$ 15 years old, and are candidates to receive TB preventive therapy (i.e. LTBI with no evidence of active TB). Key exclusion criteria include HIV infection, abnormal liver enzymes, neutropenia, previous treatment of TB or latent TB infection, or a history of study drug intolerance.

Study sites:

- Health units from Rio de Janeiro Health Secretariat and NAPDOT study clinic, Rio de Janeiro, Brazil
- Fundação de Medicina Tropical (FMT) Dr. Heitor Vieira Dourado; Manaus, Amazonas State, Brazil

Figure 1. Study Schema

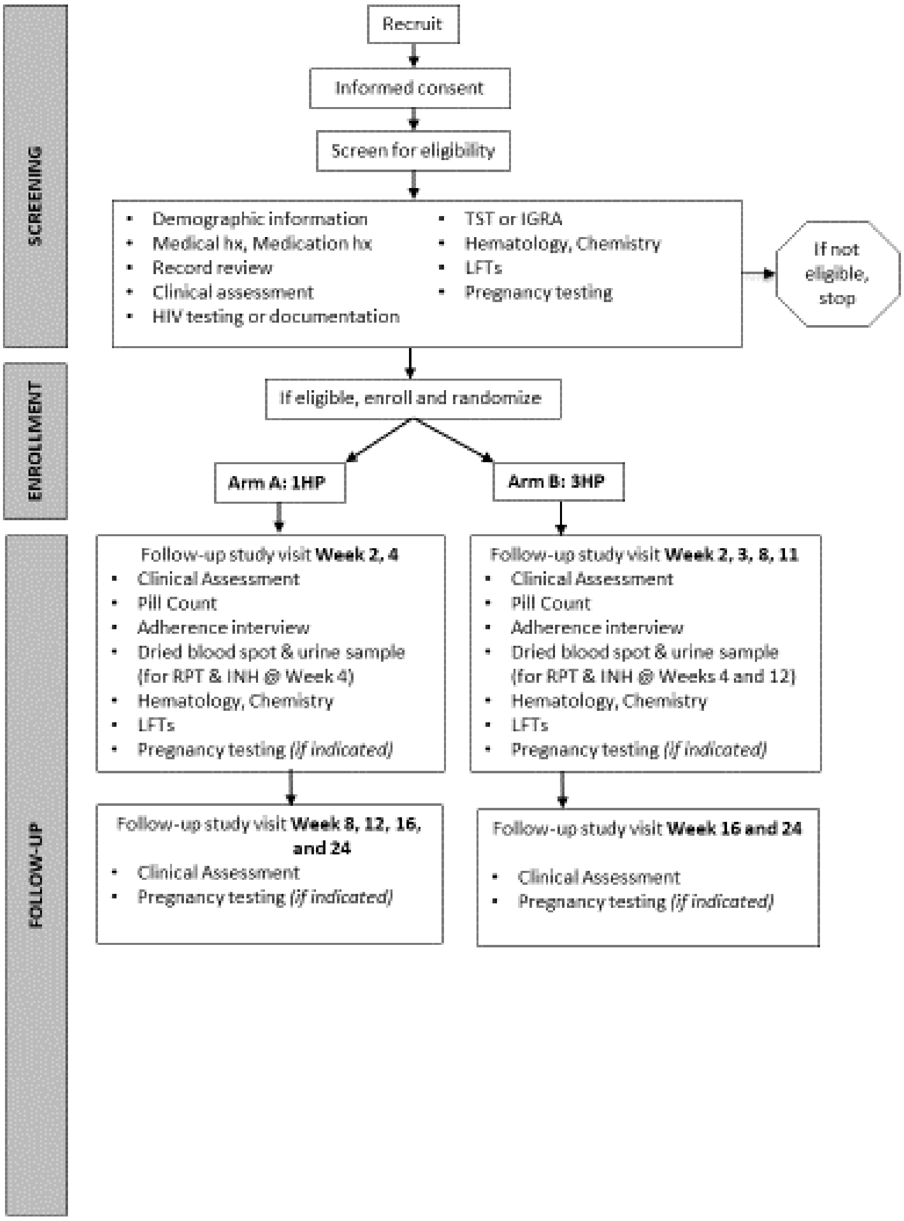

5. RANDOMIZATION

The Stata randomization code was run and saved in a file by S. Cohn, stratified by the two sites, with randomly permuted blocks of several random block sizes (details not given here for reasons of masking security). Eligible patients who meet all of the inclusion criteria and none of the exclusion criteria will be randomized in a 1:1 ratio to the study arms. Contacts of the

same index case will be considered a cluster and assigned to the same treatment arm according to the randomization of the first enrolled contact. Cluster (contacts of an index patient) size is limited to 2 participants per index case due to the diminishing returns in terms of power per enrollee within a household.

Randomization will be stratified by study site. After household contacts have signed informed consent forms, they will be randomized as a cluster to the same study arm, i.e. all receiving the same regimen. Other household members subsequently identified will not be eligible for the study.

The study is unblinded for practical reasons owing to differing pill counts between the arms and adherence being essential to the primary outcome.

6. SAMPLE SIZE CONSIDERATIONS

The sample size target is 500 evaluable adolescents and adults. Having each participant complete all of the scheduled drug sampling evaluations will provide approximately 1000 rifapentine and isoniazid pharmacological data points.

Assumptions, Outcome 1:

Successful treatment with 3HP = 80%
In clinical trials, reported completion rates of 3HP have ranged from 84-92% with directly observed therapy. In the CDC-sponsored iAdhere Trial, completion of self-administered 3HP was only 74-76%. In this study, which will use self-administered treatment, we estimate a completion rate of 80%. (Belknap 2017)

Successful treatment with 1HP = 90%
Completion of self-administered 1HP was 97% in the BRIEF TB Study, which was undertaken at research clinics in the ACTG and IMPAACT networks. We estimate that in a more real-world setting adherence will be slightly lower, but should still be at least 90%. We believe that demonstrating ≥90% completion for 1HP, significantly greater than with 3HP, will provide compelling evidence for clinical practice and treatment guidelines to change.

Based on these assumptions, with an average cluster (household contacts of the index case) size of nearly 2, and an outcome coefficient of variation (CV) of 0.15, near the mean bound using the maximum entropy distribution (Chatfield 2020), a sample size of 250 individuals per arm, 500 total, would provide 80% power for detecting a significant difference at a two-sided 0.05 significance level. The sample size is not inflated for losses to follow up, as those who fail to complete the protocol will have reached a study endpoint.

Assumptions, Outcome 2:

Proportion of participants experiencing Grade 2 or higher targeted safety events with 3HP = 13%.

In TBTC Study 26 and the iAdhere Study, rates of drug-related adverse events with 3HP were 7-8%. In clinical cohort studies, rates of discontinuation for adverse events have been higher. An analysis of patients taking 3HP in

Taiwan found that treatment discontinuation due to adverse events occurred in 12%. (Chan, ERJ 2018) A study of health care workers in South Korea reported that 19% of those receiving 3HP had hypersensitivity reactions and 15% discontinued 3HP due to drug reactions. (Jo 2019) We conservatively assume the rate will be 13%.

Proportion of participants experiencing Grade 2 or higher targeted safety events with 1HP = 6%.

In the BRIEF TB trial the rate of targeted safety events was 3%. We would want to demonstrate a reduction from 13% to at least as low as 6% in HIV-negative patients. With a CV of 0.1 (again, in reference to the maximum entropy distribution for the corresponding proportions, which are lower here than for the previous outcome), and the sample size of 250 individuals (about 125 households) in each study arm, we will have about 80% power for detecting a significant difference at a two-sided 0.05 significance level. We would also have 80% power to distinguish between risks of 12% and 5% in the two study arms. Although for convenience we have phrased this in terms of an hypothesis test, our goal is to estimate the difference in safety risks. With this sample size, and risks of 13% and 6% with 3HP and 1HP, respectively, the 95% CI for the risk difference will have a half-width of 3 percentage points. Reporting this CI will enable readers to make their own conclusions regarding non-inferiority of 1HP with respect to 3HP, in addition to superiority. (Dunn 2018)

7. STUDY POPULATION

The study will enroll HIV-uninfected individuals who are ≥ 15 years old, weight ≥ 40 kg, and are candidates to receive TB preventive therapy (i.e. LTBI with no evidence of active TB). The primary target population will be household contacts of newly diagnosed TB patients, recruited from primary care clinics. Analyses of the primary adherence and safety outcomes will be made using data from all enrollees collected through 24 weeks on study.

7.1 Inclusion/Exclusion Criteria

7.1.1 Participant Inclusion Criteria

1) Positive tuberculin skin test or interferon-gamma release assay (IGRA) test <u>and</u>

2) Household contact of an infectious TB case within previous 90 days, defined as sleeping at least once in a residence with a person diagnosed with pulmonary TB; or

3) Documented conversion of TST/IGRA from negative to positive within 2 years

7.1.2 Participant Exclusion Criteria

1) Documented HIV infection
2) Evidence of active tuberculosis on clinical exam or chest x-ray
3) Known intolerance of any study drug
4) Treatment for active or latent TB in the past for more than 14 days
5) Known close contact to someone with INH or rifampin resistant TB
6) Active liver disease or AST/ALT >3 times ULN
7) Neutropenia (ANC <1000)
8) Peripheral neuropathy >Grade 1 by DAIDS Grading Table
9) Pregnant or breastfeeding. Women of childbearing potential must agree to use non-hormonal contraception during study treatment.
10) Weight <40 kg
11) At risk of poor outcome based on clinical judgment and discretion of investigator.
12) Required use of a prohibited medication with a serious drug-drug interaction with rifamycins or isoniazid.

8. ANALYSIS POPULATION

Analyses will be intention-to-treat for both primary outcomes of successful completion and safety, slightly modified in that outcomes will begin being counted from the time of the initial study article dose, which will be supervised by the study staff in clinic.

9. FORMAL INTERIM ANALYSIS

No formal interim analysis for treatment success or safety is planned.

10. STATISTICAL METHODS

10.1 General Considerations

Stata 16.1, R 4.0, and SAS 9.4 or later versions will be used for conducting the main study analyses.

10.2 Outcome Definitions

10.2.1 Treatment completion and successful treatment

1HP

Completion of therapy is defined as having completed 28 daily doses of treatment within 6 weeks. Treatment success is defined as having taken at least 25 doses of medication confirmed by patient report and pill count, and having rifapentine or isoniazid detected at least once in urine or DBS specimens.

3HP

Completion of therapy is defined as having completed at least 12 weekly doses of treatment within 18 weeks. Treatment success is defined as having taken at least 11 doses of medication confirmed by patient report and pill count, and having rifapentine or isoniazid detected at least once in urine or DBS specimens.

Figure 2 shows the algorithm that will be used to classify patients as having treatment success of failure. This hierarchical approach begins with self-reported adherence, then uses pill count, and finally a composite of test results for isoniazid in urine and rifapentine and des-RPT in DBS.

Figure 2 Treatment Success

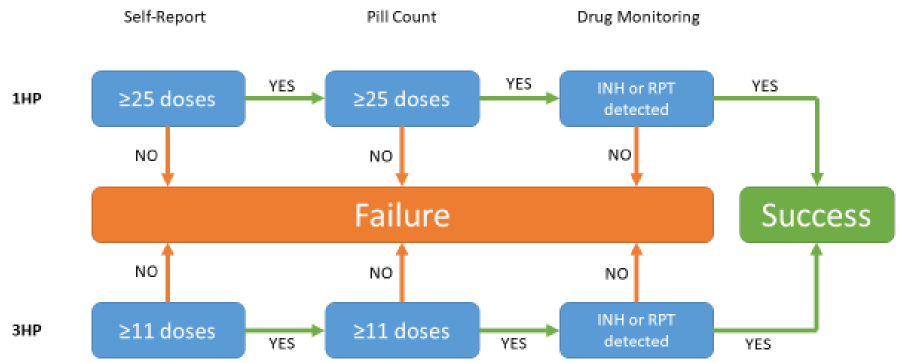

For the treatment success outcome, note that when loss to follow up precludes being able to demonstrate the requisite number of doses having been taken, the outcome is classified as “unsuccessful.” A sensitivity analysis will be conducted among those who complete their last scheduled visit after 23 weeks on study.

10.2.2 Safety: Numbers of participants with at least one occurrence of Grade 2 or higher targeted safety events and/or discontinuation of study medications because of side effects attributed to study medications.

Targeted safety events are hypersensitivity syndrome, rash, peripheral neuropathy, hepatotoxicity, nausea and vomiting, and drug-related fever.

This study will grade toxicity and adverse events according to the *Division of AIDS (DAIDS) Table for Grading Adult and Pediatric Adverse Events, Corrected Version 2.1* (dated July 2017).

https://rsc.niaid.nih.gov/sites/default/files/daidsgradingcorrectedv21.pdf
See Appendix V of the study protocol.

For the primary safety outcome as of 24 weeks, those who do not complete their last scheduled visit after 23 weeks on study will be considered as failures (safety events).

10.3 Aim-specific analyses

Tests of statistical significance will not be undertaken for baseline characteristics; rather, the clinical importance of any imbalance will be noted.

10.3.1 Treatment Success and Safety

A superiority framework will be used for these analyses, with statistical significance defined as p<0.05 for each of the two primary outcomes. No multiplicity correction will be employed.

For each of the primary outcomes, if fewer than 5% of failures (unsuccessful treatment; specified Grade 2 event) occur in households with 2 or more failures, p-values will be calculated using Fisher's exact test, and 95% confidence intervals (CI) for risk differences will be calculated using the standard asymptotic formula.

Otherwise, within-household correlation will be accounted for using generalized estimating equations with logit link (for p-value) or identity link (for risk difference CI) and exchangeable correlation structure. If there are convergence problems with the risk difference estimation, a log link function will be used, with delta-method-constructed confidence intervals for risk differences.

Subgroup analyses will be performed by:

Site
Sex
Age (dichotomized at overall median)
Weight (dichotomized at overall median)

Interaction tests will assess differential effects.

10.3.2 Cost-Effectiveness

The primary outcome for the cost-effectiveness analysis will be the incremental cost-effective ratio (ICER) per disability-adjusted life-year (DALY) averted by 1HP versus 3HP. The incremental cost-effectiveness ratio is defined as (total cost 1HP – total cost 3HP) / (total DALYs averted 1HP – total DALYs averted 3HP). Similar ICER estimates will be calculated to compare incremental DALYs averted by 1HP and 3HP versus 6H and no treatment. Additionally, the cost per treatment completed and cost per adverse event averted will be calculated for 1HP and 3HP, alongside estimates of the ICER values for each of these secondary cost-effectiveness outcomes comparing all treatment strategies.

Cost-effectiveness will be estimated using a Markov model to simulate a population of individuals eligible for TPT under each treatment strategy. Cost and effectiveness parameters (e.g. treatment costs, adherence, treatment completion) for the model will be taken from empiric values observed at each health unit participating in the Curto study. One-way and multi-way sensitivity analysis will be used to evaluate cost-effectiveness on specific model parameters, including but not limited to: discount rate, currency conversion rate, cadre of staff providing services, price of rifapentine, 1HP and 3HP completion rates, and 1HP and 3HP efficacy. A probabilistic sensitivity analysis using Monte Carlo simulation will be used to simulate the Markov model across ranges of all input parameters and build estimates of uncertainty around each cost and cost-effectiveness outcome. For each iteration, the Monte Carlo simulation will sample with replacement from a beta distribution for all model parameters empirically observed across Curto health units, with the mode at the median value observed. Uncertainty will be presented as 95% uncertainty intervals, defined as the observed value at the 2.5th and 97.5th percentiles for each outcome across the Monte Carlo iterations.

Using the results of the Monte Carlo simulation, we will construct incremental cost-effectiveness ratio planes and cost-effectiveness acceptability curves (CEACs) to demonstrate the probability of each strategy being cost-effective at different willingness-to-pay (WTP) thresholds. While we will consider a range of WTP thresholds in the primary analysis, we will use as our primary WTP threshold the most recent objective data on Brazil's country-specific WTP threshold. We will further report the probability of being cost-effective at WTP thresholds in $500 increments ($500, $1000, etc). The ICER per DALY averted comparing 1HP to 3HP will be used for the primary ICER plane and CEAC.

Costs will be evaluated from the societal perspective, incorporating both costs to patients and to the healthcare system. Research costs will be excluded. Estimates of DALYs will be derived by combining literature values (TPT long-term effectiveness (Sterling, 2011), disability weights (Salomon, 2015)) with trial-based observations. We will use an analytic horizon of 5 years for tuberculosis events, with a lifetime horizon for TB-related sequelae, including death. The primary analysis will annuitize costs linearly using a 3% annual discount rate based on estimated life-years. In the analysis, costs will be presented in the latest year in which costs are measured during the trial, with costs collected in earlier years inflated using the World Bank's gross domestic product deflator for Brazil (https://data.worldbank.org/indicator/NY.GDP.DEFL.KD.ZG?locations=BR). All costs will then be converted to United States Dollars using the median World Bank conversion rate for that year (https://data.worldbank.org/indicator/PA.NUS.FCRF?locations=BR).

Time needed for care
As a part of economic data collection, time-and-motion (TAM) studies will be used to evaluate the time needed to provide comprehensive care for patients at each health unit. Sampling for TAM data collection will be stratified to ensure a broad coverage of visits by time of day, day of week, and time of year, plus inclusion of participants who require additional clinical evaluation (e.g., for side effects) so that the data represents a range of clinical operational conditions in our study setting. The TAM data will be analyzed and reported as median unit time with inter-quartile ranges (IQRs) per 1) discrete activity and 2) total health care staff person-time required (reported by types of personnel) for one patient throughout his/her LTBI care cascade (from initial diagnosis and clinical evaluations to treatment completion, inclusive of management of side effects). These time estimates will include both the direct (e.g. patient's face-to-face interaction with health care staff) and indirect (e.g. administrative efforts such as chart reviews and data reporting) efforts relevant for programmatic component of the patient event schedules of the Ultra Curto trial.

Time estimates will be presented separately for each health unit participating in the Ultra Curto trial. Differences in time requirements and cadre of staff completing activities across sites will be noted descriptively, but no formal statistical analysis will be performed to evaluate differences in time requirements across sites.

10.3.3 Tuberculosis

Incidence of active tuberculosis will be evaluated as an exploratory outcome. Incidence will be compared using Kaplan-Meier estimates at 24 weeks after enrolment and Greenwood's variance formula. This will be a sufficiently rare event that within-household correlation will be ignorable. Censoring will be at earliest of last study visit or death. As a sensitivity analysis, TB-free survival will also be analyzed.

REFERENCES

Belknap R, Holland D, Feng PJ, et al., TB Trials Consortium iAdhere Study Team. Self-administered Versus Directly Observed Once-Weekly Isoniazid and Rifapentine Treatment of Latent Tuberculosis Infection: A Randomized Trial. Ann Intern Med. 2017;167:689-697.

Chan PC, Lee PH, Lu MJ, Huang YC, Feng TY, Chen WW, Lee CC, Huang YF, Chiang CY. Tolerability of rifapentine-based regimens in latent tuberculosis infection treatment in the elderly. Eur Respir J. 2019 Mar 18;53(3):1802396.

Chatfield MD, Farewell DM. Understanding between-cluster variation in prevalence, and limits for how much variation is plausible. Stat Meth Med Res. 2021;30(1):286-298.

Dunn DT, Copas AJ, Brocklehurst P. Superiority and non-inferiority: two sides of the same coin? Trials 2018; 19:499.

Kyung-Wook Jo 1, Ju Sang Kim 2, Hyouk-Soo Kwon 3, Yea Eun Park 1, Ja Young Kim 4, Min Jee Hong 4, Tae Sun Shim Adverse event and treatment completion rates of a 12-dose weekly isoniazid and rifapentine course for South Korean healthcare workers Respir Med 2019 Oct-Nov;158:42-48.

Salomon JA, Haagsma JA, Davis A, et al. Disability Weights for the Global Burden of Disease 2013 study. Lancet Glob Health. 2015;3(11):e712-23.10.1016/s2214-09x(15)00069-8

Sterling TR, Villarino ME, Borisov AS, et a l. Three Months of Rifapentine and Isoniazid for Latent Tuberculosis Infection. The New England journal of medicine. 2011;365(23):2155-2166.

Sun HY, Huang YW, Huang WC, Chang LY, Chan PC, Chuang YC, Ruan SY, Wang JY, Wang JT. Twelve-dose weekly rifapentine plus isoniazid for latent tuberculosis infection: A multicentre randomised controlled trial in Taiwan. Tuberculosis (Edinb). 2018 Jul;111:121-126.
