# Supplementary material for: Acceptability and safety of one versus three months of rifapentine and isoniazid to prevent tuberculosis in people exposed in the household or workplace in Brazil: The Ultra-Curto randomized controlled trial
Source: PLoS Med. 2026 Feb 10;23(2):e1004758. doi: 10.1371/journal.pmed.1004758 (PMC12890141; doi:10.1371/journal.pmed.1004758)
Supplement: S3 Text — (DOCX) [file pmed.1004758.s006.docx]

Protocol 3.0 _ April 01.2021

Protocol vr. 3.0 to vr 3.1

**Amendment 01**

- There was a change in the central coordination of the study, which will be carried by Beatriz kohler
- At the request of the NIH – National Institute of Health) its committee of clinical research experts, the DSMB (Data and Safety Monitoring Board) we, standardize and adjust the maximum treatment time with the study regimens at 50%increment. Therefore, for 1HP (arm A), the maximum time for completion of treatment will be up to 6 weeks and, for 3HP (arm B), up to 18 weeks.
- Due to the writing error, they had included the application of the informed consent in the index cases of tuberculosis, however, our study population is only the intra-household contacts of confirmed cases of TB. In this way, we corrected in the protocol that we will only apply ICF to intrahousehold contacts.

Protocol vr.3.1- Nov 24,2021

Protocol vr_ 3.1 to vr. 4.0

**Amendment #2**

- 6.2.1Laboratory tests: the “bicarbonate” test was excluded from the study participants’ assessment so as not to delay the start of preventive therapy. Bicarbonate is a test that assesses acid-base balance, useful in monitoring critically ill patients, especially in intensive care settings.

This test requires a longer processing time (up to five business days), which has

delayed the start of preventive therapy for tuberculosis in participants. Considering the low risk of acid-base disorders in participants, the other tests provided for in the protocol (creatinine, sodium, and potassium) are sufficient for clinical evaluation.

- 12.3.8 Missed visit: completion of the record**.** In the description of what a missed visit is, in its current form, it is not clear what should be considered for recording this type of visit.

Protocol Vr. 4.0 _ Oct 14, 2022

Protocol vr. 4.0 to vr. 5.0

**Amendment# 03**

This version of the protocol has been amended to clarify the definition of the primary safety endpoint of the trial, and to clarify dates for collection of pharmacological monitoring samples from participants in Arm B. We have also change the manner in which participants for the costing survey will be selected – it will be a convenience sample, not by random selection. Several other minor grammatical and spelling errors have been corrected, and the list of investigators has been updated.

Pages 15 and 19 – The primary safety outcome definition has reworded to: “Safety of treatment regimens, defined as occurrence of Grade 2 or higher targeted safety events while taking study medications or within 14 days of stopping, or discontinuation of study medications because of side effects.”

This limits targeted safety events to those which occur on study medications or within 2 weeks of discontinuing. It also removes the work “and” from the final clause, clarifying that discontinuation of study medications because of side effects is by itself a safety outcome.

Pages 14 and 37 – Timing of blood and urine collection for Arm B. The time of the two study visits at which blood and urine are collected to test for drug metabolites has been corrected to reflect the actual day on which the 4^th^ and 12^th^ weekly doses of 3HP are taken. Because the first week’s dose is given on Day 1, the actual date of the 4^th^ weekly dose is Day 21, not Day 28. Similarly, the 12^th^ weekly dose is taken at Day 77, not Day 84, and so the date of the study visit has been clarified. Participants in this arm of the study have been having their samples collected on the correct day (Day 22), but the wording of the protocol was imprecise and confusing.

Pages 34, 35, and 37 – Selection of participants for costing survey. The protocol has been amended to specify that participants for the costing survey will be selected based on convenience, rather than randomly chosen. The process of random selection has been deemed to be too complex, based on experience with a similar study carried out by our Brazilian collaborators, and the study team does not believe random selection is necessary to obtain valid data.

Protocol Vr. 5.0 _ July 04,2023
